# Mouse promoters are characterised by low occupancy and high turnover of RNA polymerase II

**DOI:** 10.1038/s44320-025-00094-5

**Published:** 2025-03-31

**Authors:** Kasit Chatsirisupachai, Christina J I Moene, Rozemarijn Kleinendorst, Elisa Kreibich, Nacho Molina, Arnaud Krebs

**Affiliations:** 1 Genome Biology Unit, EMBL Meyerhofstaße 1, 69117 Heidelberg, Germany; 2 Institut de Génétique et de Biologie Moléculaire et Cellulaire (IGBMC), Université de Strasbourg; Centre National de la Recherche Scientifique (CNRS) UMR 7104; Institut National de la Santé et de la Recherche Médicale (INSERM) UMR-S 1258, 1 Rue Laurent Fries, 67404 Illkirch, France; 3 Present Address: Division of Gene Regulation, Netherlands Cancer Institute, 1066 CX Amsterdam, The Netherlands; 4 Present Address: Oncode Institute, Utrecht, The Netherlands; 5 Present Address: ETH Zürich, Department for Biosystems Science and Engineering (D-BSSE), Basel, Switzerland

**Keywords:** Eukaryotic Transcription, Kinetic Modelling, RNA Pol II, Pol II Pausing, Single-molecule Genomics, Chromatin, Transcription & Genomics

## Abstract

The general transcription machinery and its occupancy at promoters are highly conserved across metazoans. This contrasts with the kinetics of mRNA production that considerably differ between model species such as *Drosophila* and mouse. The molecular basis for these kinetic differences is currently unknown. Here, we used Single-Molecule Footprinting to measure RNA Polymerase II (Pol II) occupancy, the fraction of DNA molecules bound, at promoters in mouse and *Drosophila* cell lines. Single-molecule data reveals that Pol II occupancy is on average 3–5 times more frequent at transcriptionally active *Drosophila* promoters than active mouse promoters. Kinetic modelling of the occupancy states suggests that these differences in Pol II occupancy are determined by the ratio between the transcription initiation and Pol II turnover rates. We used chemical perturbation of transcription initiation to determine Pol II turnover rate in both species. Integration of these data into the model shows that infrequent Pol II occupancy in mouse is explained by the combination of high Pol II turnover and low transcription initiation rates.

## Synopsis

Single-Molecule Footprinting reveals that RNA polymerase II (Pol II) occupancy is infrequent at mouse promoters. This results from a combination of higher Pol II turnover and lower transcription initiation at mouse compared to *Drosophila* promoters.

- Low Pol II occupancy at highly active mouse promoters, compared to *Drosophila* promoters, is observed through the analysis of Single-Molecule Footprinting data.
- A mathematical model is employed to describe Pol II occupancy as a ratio between Pol II turnover and transcription initiation rates.
- A higher Pol II turnover rate at mouse promoters is detected through the inhibition of transcription initiation.
- The differences in Pol II turnover rates are insufficient to fully explain the lower Pol II occupancy at mouse promoters; the remaining discrepancy is attributed to differences in transcription initiation.

## Abstract (web summary)

Single-Molecule Footprinting reveals that RNA polymerase II (Pol II) occupancy is infrequent at mouse promoters. This results from a combination of higher Pol II turnover and lower transcription initiation at mouse compared to *Drosophila* promoters.

## Graphical abstract

**Figure:**
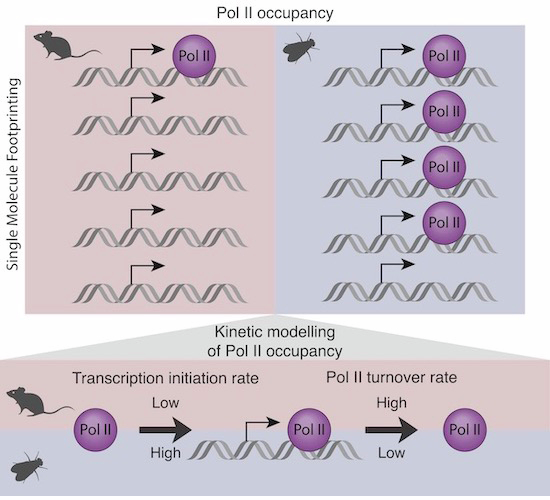

**Figure:**
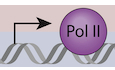

## Introduction

Precise control of the levels and the timing of gene expression is required for the successful development and homoeostasis of organisms. The production of a transcript by RNA Polymerase II (Pol II) is a sequential process started by the opening of the chromatin at promoters and the binding of general transcription factors (GTFs), which form the pre-initiation complex (PIC) and recruit Pol II onto the DNA. Upon initiation, Pol II transcribes for a few nucleotides and pauses to enable mRNA capping. Pol II is then either released into elongation to produce a full-length transcript or terminated by the integrator complex (Bentley, 2025; Cramer, 2019). Transcription is a discontinuous process that occurs in bursts. Active genes typically switch between an active state where transcription occurs, and inactive states during which the gene is silent (Tunnacliffe and Chubb, 2020). Each gene has a characteristic bursting pattern with a given frequency and intensity that, together with the mRNA decay rate, defines the steady-state levels of mRNA in cells (Larsson et al, 2019; Ochiai et al, 2020; Ramsköld et al, 2024). Although gene bursting is conserved across eukaryotes, it happens on strikingly distinct timescales in different organisms. For instance, in *Drosophila* embryos, the duration of the active and inactive states are in the same order of magnitude at the minute scale (Garcia et al, 2013; Hamamoto et al, 2023; Lagha et al, 2013; Pimmett et al, 2021), while mammalian genes typically have long inactive states, ranging from tens of minutes to hours, interspersed with short active states (Rodriguez et al, 2019; Stavreva et al, 2019; Suter et al, 2011; Wan et al, 2021). The molecular basis for these wide differences in transcription kinetics is yet unknown (Lammers et al, 2020; Meeussen and Lenstra, 2024).

A possible explanation could be the divergence in the molecular mechanisms underlying transcription. Yet, biochemical and structural data argue for a high degree of conservation of the protein complexes regulating the various steps of transcription across metazoans. For instance, the protein complexes controlling transcription initiation, namely the PIC and mediator complex, are highly conserved from yeast to human (Abdella et al, 2021; Aibara et al, 2021; Hantsche and Cramer, 2017; He et al, 2013; Mühlbacher et al, 2014; Nogales et al, 2017). In addition, the factors that control entry into elongation through polymerase pausing, such as the DRB sensitivity-inducing factor (DSIF), the negative elongation factor (NELF), and the positive transcription elongation factor b (P-TEFb), as well as the Integrator complex that mediates early transcription termination, are conserved across metazoans (Chivu et al, 2024; Stein et al, 2022; Vos et al, 2016; Welsh and Gardini, 2023).

An alternative hypothesis is that the same set of molecular complexes are used, yet with different kinetics. The long periods of transcriptional inactivity characterising mammalian genes suggest that, at any given time, promoters will be active in only a small fraction of cells within a population. This could be explained by the fact that promoters are experiencing initiation less frequently and consequently have lower occupancy by GTFs and Pol II. Another explanation could be that genes have similar rates of initiation, but that they are more frequently subject to non-productive transcription through increased rates of Pol II pausing or premature transcription termination. Pol II was found to accumulate shortly downstream of the active genes transcription start site (TSS) in both *Drosophila* and mice (Chivu et al, 2024; Henriques et al, 2013; Williams et al, 2015), suggesting no striking differences in the distribution of Pol II at sites of initiation, pausing or entry into elongation. Thus, there is an unresolved paradox between the large difference in the kinetics of transcription and the similarity of the molecular mechanisms supporting this process. It illustrates the challenges in connecting data from dynamic measurements of transcription kinetics with the static view of the genome occupancy of transcription regulators obtained by genomics.

**Figure 1:**
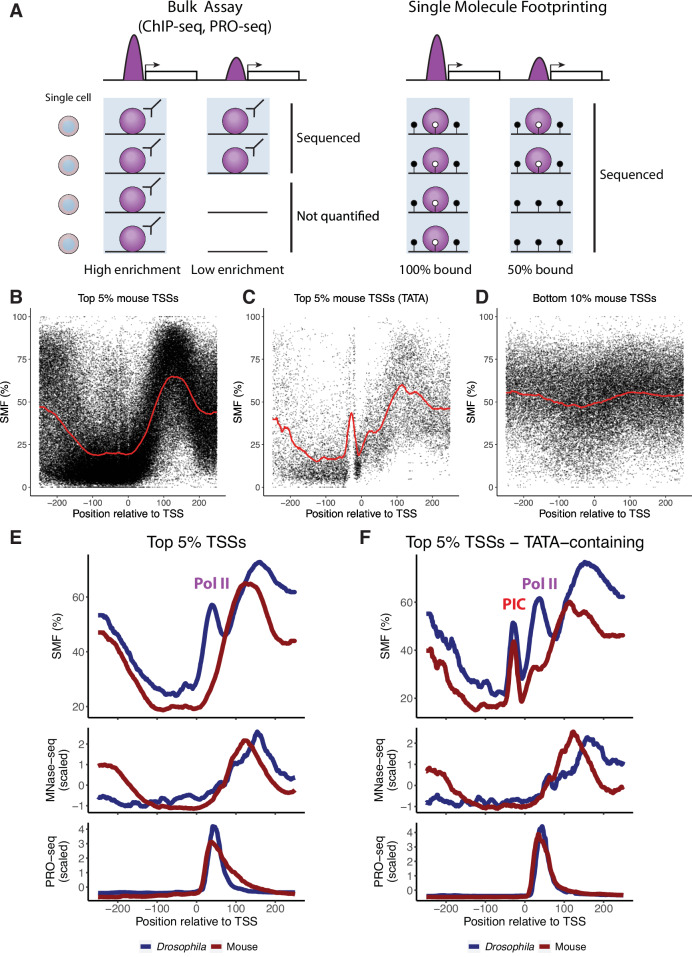
SMF reveals the difference in average Pol II occupancy at mouse TKO mESC and *Drosophila* S2 cell promoters. (**A**) Difference in data modality between bulk genomic assays and the single-molecule assay. While methods such as ChIP-seq or PRO-seq report an enrichment, SMF allows absolute quantification of binding frequencies in the cell population. The light blue box indicates the DNA fraction that is sequenced with an assay. (**B–D**) Composite profile of SMF signal (1 - methylation [%]) at the (**B**) active mouse TSSs (top 5% Pol II ChIP-seq, *n* = 1316 promoters), (**C**) active TATA-box containing mouse TSSs (top 5% Pol II ChIP-seq with a TATA-box, *n *= 151 promoters), and (**D**) inactive mouse TSSs (bottom 10% Pol II ChIP-seq, *n* = 2420 promoters). Shown is the footprinting frequency (1 - methylation [%]) of individual cytosines (black dots). The red line indicates the smoothed average signal over 20 bp. (**E**,** F**) Comparison of the average SMF, MNase-seq, and PRO-seq levels between mouse (red lines) and *Drosophila* (blue lines) of (**E**) top 5% TSSs (*n* = 1316 promoters for mouse, *n* = 931 promoters for *Drosophila*) and (**F**) top 5% TATA-containing TSSs (*n* = 151 promoters for mouse, *n* = 152 promoters for *Drosophila*). For MNase-seq and PRO-seq, reads at each position relative to the TSS [-250:250] were normalised to reads per million (RPM). The RPM at each position relative to the TSS were averaged across top 5% and top 5% TATA-containing TSSs. The average RPMs were then normalised to the Z-score to enable a comparison between mouse and *Drosophila* in the same plots.

Genome-scale studies of GTF and Pol II occupancy have been mostly based on bulk genomics assays such as chromatin immunoprecipitation sequencing (ChIP-seq) or precision run-on sequencing (PRO-seq). These assays can reveal the relative occupancy of factors across the genome at nearly base pair resolution (He et al, 2015; Rhee and Pugh, 2011; Rossi et al, 2021; Shao and Zeitlinger, 2017), and they have been widely used to compare the relative abundance of Pol II at different promoters or between different experimental conditions. However, a limitation is that these assays are based on the sequencing of DNA that is experimentally enriched, representing an average occupancy over millions of cells. In addition, the enrichment of a protein such as Pol II at a promoter cannot be translated to the proportion of cells in which this binding occurs (Fig. 1A, left). This makes it challenging to use these methods to compare the absolute promoter-proximal protein binding levels between species with distinct genomes, such as invertebrates and mammals.

Single-Molecule Footprinting (SMF) combines the use of cytosine methyltransferases and bisulfite sequencing to quantify protein-DNA contacts at single-molecule resolution across the genome (Kleinendorst et al, 2021; Krebs et al, 2017). Approaches with similar principles, such as Fiber-seq (Stergachis et al, 2020), SMAC-seq (Shipony et al, 2020), and SAMOSA (Abdulhay et al, 2020), have been developed to study chromatin accessibility, nucleosome, and protein occupancy across various cell types from yeast to mammals. The single-molecule resolution of SMF and these approaches enables the direct quantification of the frequency of promoter occupancy by various DNA binding proteins, including transcription factors (TFs), GTFs, and Pol II (Fig. 1A, right) (Krebs et al, 2017; Sönmezer et al, 2021). We showed that Pol II binding frequency measured by SMF scales with enrichments determined using orthogonal measures, such as ChIP-seq or PRO-seq (Krebs et al, 2017). More recently, Tullius et al successfully employed Fiber-seq, a single-molecule, long-read genomic footprinting using adenine methyltransferase, to study the interplay between Pol II and nucleosome footprints across long DNA molecules in *Drosophila* S2 cell line (Tullius et al, 2024). While the measurement from these single-molecule genomics approaches is not time-resolved, the frequency of each state in the cell population is a function of how often and how long each transcriptional intermediate occurs over time. Thus, quantification of the frequency of Pol II occupancy across species could enable us to identify the molecular steps that cause the difference in transcription kinetics between species.

Here, we used SMF to quantify PIC and Pol II occupancy at the promoters in mouse and *Drosophila* cell lines, two species with divergent transcription kinetic rates. While not observed in bulk assays, SMF data revealed that transcriptionally active mouse promoters are characterised by 3–5 times lower Pol II occupancy levels at the pausing site, than those in *Drosophila*. We applied mathematical modelling to infer Pol II kinetics at promoters from Pol II occupancy. To determine the relative contribution of each step of the process, we measured the changes in promoter-proximal Pol II levels upon inhibition of transcription initiation. We found that the turnover rate of Pol II at mouse promoters is higher than in *Drosophila*, but that these differences are insufficient to entirely explain the low occupancy levels observed in mouse cells. In turn, the integration of these Pol II turnover rates into our model suggests that differences in the transcription initiation rate between species also significantly contribute to the differences in Pol II occupancy.

## Results

### Low amplitude Pol II footprints at mouse promoters

We have previously shown that SMF can be used to quantify the occupancy of nucleosomes, PIC, and Pol II at *Drosophila* promoters, and their dynamics across cell types (Krebs et al, 2017). We now used a similar strategy to analyse a previously generated bait-capture SMF dataset in mouse embryonic stem cells (mESCs), that covers most of the annotated mouse promoters at high coverage (median of 100 molecules) (Sönmezer et al, 2021; Data ref: Sönmezer et al, 2021). To mitigate potential bias in the assay resulting from the difference in genome size between species, the number of cells used in the SMF protocol was adjusted to maintain a constant substrate DNA in the reaction (Kleinendorst et al, 2021; Krebs et al, 2017; Sönmezer et al, 2021). Additionally, we performed the experiments under saturating enzyme conditions where sequence-specific preferences of the methyltransferase are no longer detectable (Kleinendorst et al, 2021).

We first characterised the spatial distribution of the footprints created by the occupancy of the transcription machinery at core promoters of the mouse genome. We defined the TSS based on Cap Analysis of Gene Expression (CAGE) data (Abugessaisa et al, 2017). In the case of multiple initiation sites (i.e. broad promoters in the mouse genome), we used the strongest CAGE peak (see Methods for details). We contrasted the average accessibility patterns at promoters of genes that are either highly active (top 5% Pol II ChIP-seq—Fig. 1B,C) or inactive (bottom 10% Pol II ChIP-seq—Fig. 1D). Chromatin accessibility was low at inactive promoters, while active promoters showed high accessibility upstream and a strongly phased +1 nucleosome downstream of the TSS. Additionally, we subset TATA-containing promoters from the top 5% of highly active promoters. The average profile of these TATA-containing promoters showed a strong footprint upstream of the TSS (Fig. 1C), similar to what has been observed at *Drosophila* promoters (Krebs et al, 2017; Tullius et al, 2024). We previously showed that this upstream footprint is created by PIC, as the knockdown of TATA-binding protein (TBP) attenuated this footprint in *Drosophila* (Krebs et al, 2017). Thus, the occupancy profiles of mouse promoters generally recapitulate those of *Drosophila* promoters.

However, a direct overlay of the profiles from the two species revealed several differences (Fig. 1E,F). First, the position of footprint corresponding to the +1 nucleosome downstream of the TSS is shifted by ~20 bp in mouse promoters, in agreement with previous reports (Mavrich et al, 2008), and nucleosome positions determined by micrococcal nuclease digestion and sequencing (MNase-seq) (Fig. 1E, middle panel). Second, while active *Drosophila* promoters harbour a prominent footprint downstream of the TSS at a position compatible with Pol II pausing (Krebs et al, 2017; Data ref: Krebs et al, 2017), this footprint is absent when averaging signal over mouse promoters with comparable transcriptional activity (Fig. 1E, upper panel). This difference in these downstream footprints is also visible, albeit less pronounced, when focusing on TATA-containing promoters where a low amplitude Pol II footprint is observed at mouse promoters (Fig. 1F, upper panel). The observed difference in SMF footprints at the Pol II pausing site stands in contrast with the consistency in the accumulation of active RNA polymerase measured by bulk assays such as PRO-seq when performed in the same cell lines (Fig. 1E,F, lower panel). This low Pol II footprint at mouse promoters is however consistent with lower pausing index in mouse compared to *Drosophila* (defined as the ratio of active Pol II levels measured by PRO-seq at the promoter over the gene body) (Appendix Fig. S1). We note however that the differences in SMF footprints are specific to Pol II, since the PIC footprint observable at TATA-containing promoters is almost identical between the two species (Fig. 1F, upper panel).

We next wondered if the observed differences may be a specific feature of mESCs that are in a pluripotent state. We compared the average profiles around the TSS of highly active genes in somatic cell lines representing various cell lineage (Kreibich et al, 2023; Data ref: Kreibich et al, 2023). We observed very similar profiles in all the tested cell lines, with strong footprints for the PIC and low Pol II footprints downstream of the TSS (Fig. EV1A,B). In contrast, the prominent Pol II footprint is also found at the promoters of *Drosophila* ovarian somatic cells (OSC) (Krebs et al, 2017; Data ref: Krebs et al, 2017), in addition to the S2 cells (Fig. EV1C,D). These results suggest that a low amplitude Pol II footprint is a general feature of mouse promoters. Together, these observations suggest that the spatial patterns of promoter occupancy are globally conserved from *Drosophila* to mouse, but that Pol II occupancy levels may be reduced at mouse promoters.

**Figure EV1:**

Average promoter footprint across various cell lines. Absence of Pol II footprints at highly active mouse promoters is not only restricted to TKO mESCs but also a property of other mouse cell types. Composite profile of SMF signal at (**A**) the TSSs of active promoters (top 5% Pol II ChIP-seq) and (**B**) the TSSs of active, TATA-box containing promoters (top 5% Pol II ChIP-seq with a TATA-box) in mouse wild-type embryonic stem cells (ES), neural progenitor cells (NP), myoblasts (C2C12), and erythrocytes (MEL). Prominent Pol II footprints at highly active *Drosophila* promoters in the ovarian somatic cell (OSC) line. Composite profile of SMF signal at (**C**) the TSSs of active promoters (top 5% Pol II ChIP-seq) and (**D**) the TSSs of active, TATA-box containing promoters (top 5% Pol II ChIP-seq with a TATA-box) in *Drosophila* OSCs. Shown is the footprinting frequency (1 - methylation [%]) of individual cytosines (black dots). The red line indicates the smoothed average signal over 20 bp.

### Quantification of the frequency of Pol II occupancy at mouse promoters

To quantify the frequency of promoter occupancy by the transcription machinery, we adapted the molecular classifier originally developed to study *Drosophila* promoters (Krebs et al, 2017) to account for the shift in the Pol II and nucleosome positions relative to TSS in mouse (Fig. EV2A; see Methods for details). Based on the accessibility at four bins around the TSS, each DNA molecule is classified into different promoter states according to the presence or absence of a footprint at the positions occupied by the PIC and Pol II or the occupancy of the nucleosome at these positions (Fig. EV2B). With this strategy, we determined for each promoter the frequency of molecules in each of the promoter states (unassigned, nucleosome, unbound, PIC, PIC + Pol II, and Pol II). In total, we were able to quantify promoter state frequencies of 6122 mouse promoters from the bait-capture SMF data (Dataset EV1). The resulting promoter state frequencies were consistent across replicates (Fig. EV2C), demonstrating that we sampled a sufficient number of DNA molecules covering each promoter. We further compared the frequency of the states with orthogonal measurements of Pol II or nucleosome occupancy at promoters. We found that the states were separated in two clusters corresponding to active and inactive promoter states (Fig. EV2D), consistent with our previous observations in *Drosophila* (Krebs et al, 2017). Moreover, we observed a good agreement between the frequency of the states measured by SMF and the enrichment of the respective feature in bulk assays. This demonstrates the accuracy of our quantification of the occupancy by the transcription machinery and nucleosomes at single-molecule resolution at mouse promoters.

**Figure EV2:**

Single-molecule promoter state decomposition in mouse promoters. (**A**) A four-bin strategy to sort single DNA molecule into distinct promoter states. This bin strategy is adapted from Krebs et al, 2017 to include the downstream bin to better distinguish the Pol II footprint from the nucleosome footprint. Methylation status is binarized within each bin, creating a 4-bit vector that leads to 16 possible combinations, each describing the state of each molecule (see Methods for details). (**B**) Schematic representation of the methylation patterns used to define each promoter state (methylated, accessible Cs—light grey; unmethylated, protected Cs—black). The top horizontal annotation bar represents the four bins (upstream, TATA, Pol II, downstream) used for promoter state decomposition. The vertical side bar displays the promoter state corresponding to the occupancy type according to the bins. (**C**) Scatter plots show the correlation between state frequencies determined from each bait-capture SMF replicate (*n* = 6122 promoters). The states PIC + Pol II and Pol II are combined into a single Pol II state. Pearson correlation coefficients are displayed. (**D**) The global relationship between promoter state frequencies and independent bulk measurements of Pol II and nucleosomes. The heatmap shows the Spearman correlation between each dataset, ordered by hierarchical clustering. The states segregate into two groups correlating either with transcription/transcription machinery (RNA-seq, PRO-seq, and Pol II ChIP-seq) or nucleosomes (MNase-seq).

### Mouse promoters are characterised by low frequency of Pol II occupancy

**Figure 2:**
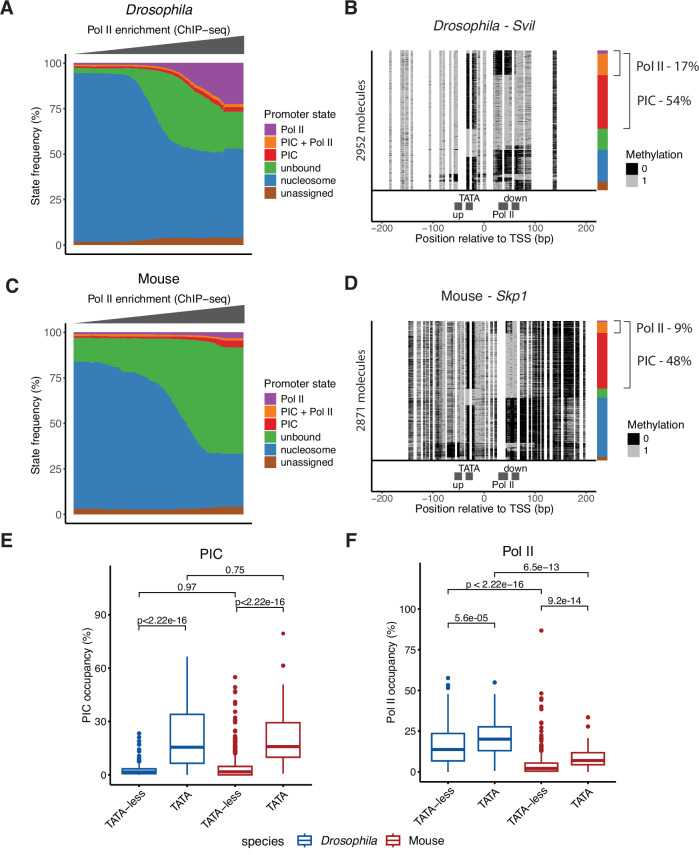
Lower Pol II binding frequency in mouse compared to *Drosophila* promoters. (**A**) Distribution of SMF-derived promoter state frequencies as a function of Pol II ChIP-seq level in *Drosophila* promoters (*n* = 5912 promoters). Promoters were first binned based on their log2 Pol II ChIP-seq signal. Within each bin, the median frequency of each of the promoter state was calculated. Each bar in the plot represents the median state frequencies of the promoters in the bin. Colour code represents each state as follows: purple—Pol II, orange—PIC + Pol II, red—PIC, green—unbound, blue—nucleosome, and brown—unassigned. (**B**) Single-locus example showing single-molecule sorting of a highly active, TATA-box containing *Drosophila* promoter (*Svil*, 94th percentile by Pol II ChIP-seq). Each row in the single-molecule stack denotes a single DNA molecule and the methylation status of each cytosine in that molecule (methylated, accessible—light grey; unmethylated, protected—black). The positions of the four bins (upstream, TATA, Pol II, downstream) used for promoter state decomposition are shown below the single-molecule stack (see Methods for details). The vertical sidebars on the right of the plot depict the frequency of each promoter state determined by single-molecule decomposition. The percentages of molecules harbouring footprints for the engaged Pol II and PIC are indicated on the right side of the plot. (**C**) Distribution of SMF-derived state frequencies as a function of Pol II ChIP-seq level in mouse promoters (*n* = 6122 promoters). Same representation as (**A**). (**D**) Single-locus example of a single-molecule sorting of a highly active, TATA-box containing mouse promoter (*Skp1*, 97th percentile by Pol II ChIP-seq). Same representation as (**B**). (**E**) Comparison of PIC state frequency between mouse (TATA-less *n* = 572 promoters; TATA-containing *n* = 64 promoters) and *Drosophila* (TATA-less *n* = 422 promoters; TATA-containing *n* = 71 promoters) promoters. Boxplots represent the distribution of the frequency of PIC-bound molecules (PIC and PIC + Pol II states) at highly active promoters (top 5% Pol II ChIP-seq level). The middle line of the box represents the median. The box displays the interquartile range (IQR), 25th to 75th percentile. Whiskers represent a distance of 1.5 × IQR. (**F**) Comparison of Pol II state frequency between mouse (TATA-less *n* = 572 promoters; TATA-containing *n* = 64 promoters) and *Drosophila* (TATA-less *n* = 422 promoters; TATA-containing *n* = 71 promoters) promoters. Boxplots represent the distribution of the frequency of Pol II-bound molecules (Pol II and PIC + Pol II states) at highly active promoters (top 5% Pol II ChIP-seq level). The middle line of the box represents the median. The box displays the interquartile range (IQR), 25th to 75th percentile. Whiskers represent a distance of 1.5 × IQR. The analysis is stratified by the presence of a TATA-box at the promoter. The statistical comparisons between groups were performed using the Wilcoxon rank-sum test.

Next, we compared the Pol II occupancies between species while accounting for their relative transcription activity. We ranked all promoters we were able to quantify with our method (mouse—6122 promoters, Dataset EV1; *Drosophila*—5912 promoters, Dataset EV2) by their activity, based on Pol II ChIP-seq signal at the promoters, and compared the distribution of promoter states between *Drosophila* and mouse (Fig. 2A–D). At *Drosophila* promoters, an increase in promoter activity is correlated with a loss in nucleosome occupancy, gain in accessibility, and an increase in Pol II binding frequency (Fig. 2A). Many highly active promoters showed ~20% of the molecules occupied by Pol II, as illustrated for the *Svil* promoter (17% - Fig. 2B). At mouse promoters, we found similar loss in nucleosome occupancy and gain in accessibility as a function of Pol II ChIP-seq. Yet, while detectable, the Pol II state frequency was much reduced, with occupancies below 10% even at the most active TSSs (Fig. 2C). For example, the *Skp1* promoter is among the most active promoters (top 3%) but only 9% of the molecules is occupied by Pol II (Fig. 2D). We additionally noted that upon activation mouse promoters are generally less occupied by nucleosome than *Drosophila* promoters (Fig. 2A,C). This difference correlates with the CpG density of the promoters (Appendix Fig. S2A,B), suggesting that differences in the CpG density at promoters between species (Deaton and Bird, 2011) may contribute to the higher frequency of nucleosome-free molecules at mouse promoters.

To quantitatively estimate the magnitude of the difference in promoter occupancy between mouse and *Drosophila*, we looked at the distribution of PIC and Pol II occupancies across the top 5% highly active promoters that we were able to quantify using SMF (mouse—636 promoters, Dataset EV1; *Drosophila*—493 promoters, Dataset EV2). We stratified this analysis based on the presence or absence of the TATA-box, as in *Drosophila* TATA-containing promoters have a higher PIC and Pol II occupancy than TATA-less promoters (Krebs et al, 2017). Consistent with *Drosophila*, the PIC occupancy is significantly increased at the TATA-containing promoters in mouse (Fig. 2E). There are, however, no significant differences in the distribution of promoter occupancy by the PIC between the two species (Fig. 2E). This is in contrast to the frequency of Pol II occupancy. The median Pol II binding frequencies at highly active *Drosophila* promoters are 14% (TATA-less) and 20% (TATA-containing), while those of mouse promoters are only 2% and 7%, respectively (Fig. 2F). This suggests that Pol II occupancy at mouse promoters is a low-frequency event, occurring in <10% of the cells at any given time even at highly active promoters.

### Low-frequency Pol II footprints are lost upon inhibition of transcription initiation

Low-frequency events such as Pol II occupancy at mouse promoters are harder to quantify by SMF, as it requires to sample more DNA molecules to observe them. To unambiguously confirm that the low-frequency footprints captured downstream of the mouse TSS are created by Pol II, we measured the change in the Pol II occupancy, the frequency of molecules in PIC + Pol II and Pol II states, upon inhibition of transcription initiation. We used triptolide (TRP) at concentrations previously shown to deplete promoter-proximal Pol II in mESCs (Jonkers et al, 2014), and performed targeted SMF against a targeted set of 47 promoters selected to cover a diverse spectrum of Pol II occupancies and promoter structures (35 TATA-containing and 12 TATA-less promoters) (Dataset EV3). This targeted approach generates thousands of single-molecule measures for each locus, that are robust for precise and reproducible quantification of the low Pol II occupancy at mouse promoters. While Pol II occupancy in this set of promoters is generally low, ranging from 0% to 20% with a median of 2.7%, we obtained a consistent estimation across replicates (Fig. EV3A). We further confirmed that the promoter state frequencies obtained from the targeted SMF are in high agreement with other orthogonal approaches (Fig. EV3B).

**Figure EV3:**

Quality control of targeted amplicon SMF sequencing. (**A**) Scatter plots illustrating the correlation between state frequencies determined from each amplicon SMF replicate. The states PIC + Pol II and Pol II are combined into a single Pol II state. Pearson correlation coefficients are displayed. (**B**) The global relationship between promoter state frequencies at selected promoters that were included in amplicon SMF (*n* = 47 promoters) and independent bulk measurements of Pol II and nucleosomes. The heatmap shows Spearman correlation between each dataset and is ordered by hierarchical clustering. States separate into two groups which either correlate with transcription/transcription machinery (RNA-seq, PRO-seq, and Pol II ChIP-seq) or nucleosomes (MNase-seq).

**Figure 3:**
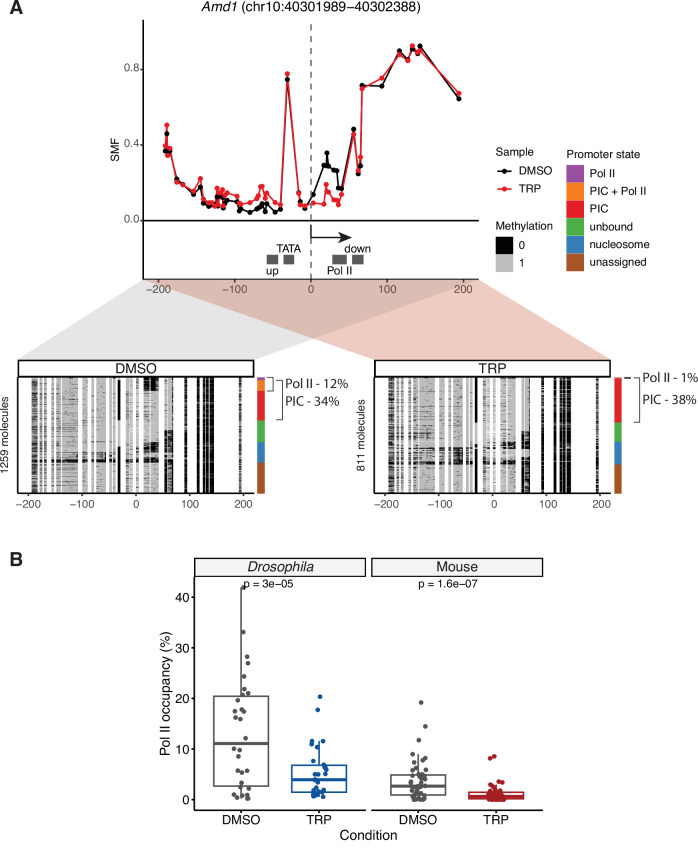
Inhibition of transcription initiation reduces Pol II footprint at mouse promoters. (**A**) Single-site example (*Amd1* promoter) shows a reduction in Pol II footprint upon triptolide (TRP) treatment. The experiment was conducted using targeted amplicon bisulfite sequencing on 47 selected promoters. The upper panel shows the average SMF plot (DMSO—black, TRP—red). The positions of the four bins (upstream, TATA, Pol II, downstream) used for promoter state decomposition are shown (see Methods for details). The *x* axis represents the position relative to the TSS, while the *y* axis shows the SMF signal (1 - methylation). The lower panel displays single-molecule stack plots for DMSO and TRP conditions. Each row denotes a single DNA molecule and the methylation status of each cytosine in that molecule (methylated, accessible—light grey; unmethylated, protected—black). The vertical sidebars display the frequency of each promoter state. The percentages of molecules harbouring footprints for the engaged Pol II are indicated on the right side of the plot. (**B**) Loss of Pol II occupancy upon inhibition of transcription initiation in *Drosophila* (*n* = 30 promoters; Krebs et al, 2017) and mouse promoters (*n* = 47 promoters). Boxplots represent the distribution of the frequency of Pol II-bound molecules (Pol II and PIC + Pol II states). The middle line of the box represents the median. The box displays the interquartile range (IQR), 25th to 75th percentile. Whiskers represent a distance of 1.5 × IQR. Statistical comparisons between groups were performed using the Wilcoxon signed-rank test. Two biological replicates of amplicon SMF were performed. For each treatment condition (control and TRP), promoter state frequencies from two replicates were averaged.

**Figure EV4:**
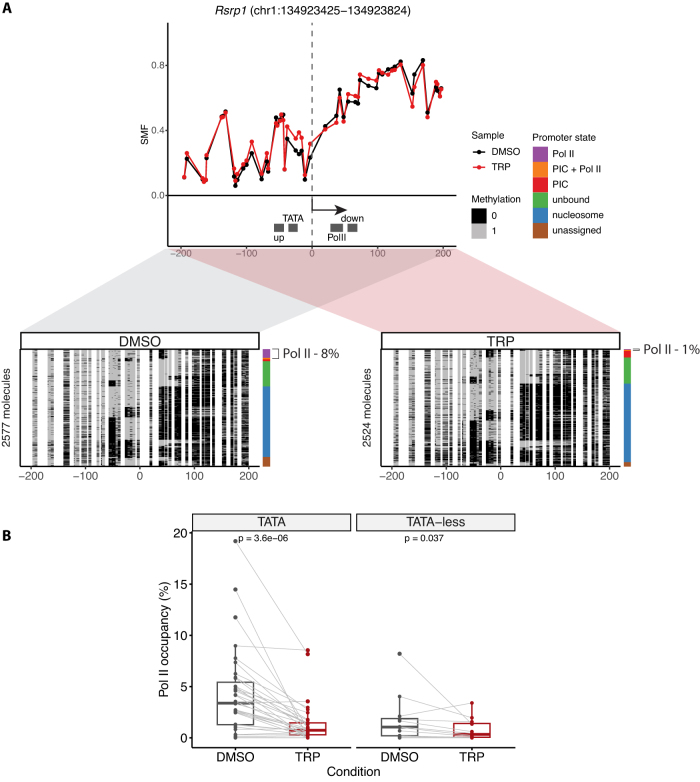
Reduction in Pol II footprint upon transcription initiation inhibition at TATA-less mouse promoters. (**A**) Single-site example of a reduction in Pol II footprint upon TRP treatment at the *Rsrp1* promoter. The upper panel shows the average SMF plot (DMSO—black, TRP—red). The positions of the four bins (upstream, TATA, Pol II, downstream) used for promoter state decomposition are shown (see Methods for details). The *x* axis represents the position relative to the TSS, while the y-axis shows the SMF signal (1 - methylation). The lower panel displays single-molecule stack plots for DMSO and TRP conditions. Each row denotes a single DNA molecule and the methylation status of each cytosine in that molecule (methylated, accessible—light grey; unmethylated, protected—black). The vertical sidebars display the frequency of each promoter state. The percentages of molecules harbouring footprints for the engaged Pol II are indicated on the right side of the plot. (**B**) Loss of Pol II occupancy upon inhibition of transcription initiation occurs in both TATA (*n* = 35) and TATA-less (*n* = 12) mouse promoters. Boxplots represent the distribution of the frequency of Pol II-bound molecules (Pol II and PIC + Pol II states). The middle line of the box represents the median. The box displays interquartile range (IQR), 25th to 75th percentile. Whiskers represent a distance of 1.5 × IQR. Statistical comparisons between groups were performed using the Wilcoxon signed-rank test.

Upon inhibition of transcription using TRP, we observed a loss of the footprint downstream of the TSS of the TATA-containing *Amd1* promoter, consistent with the loss of Pol II at the pausing site (Fig. 3A, upper panel). In contrast, the footprint upstream of the TSS, created by the PIC, remained unaffected. Both of these observations were confirmed by quantifying the PIC and Pol II occupancy at the single-molecule level, showing no significant changes in the footprint PIC state (34% and 38%), while Pol II occupancy decreased from 12% to 1% (Fig. 3A, lower panel). A reduction in the Pol II footprint was also found at the TATA-less *Rsrp1* promoter, decreasing from 8% to 1% following TRP treatment (Fig. EV4A). Extending this analysis to a larger set of 47 mouse promoters (35 TATA-containing and 12 TATA-less promoters), we observed a consistent loss of Pol II occupancy upon blocking transcription initiation by TRP treatment in mouse promoters, similar to what has been observed in *Drosophila* promoters (Krebs et al, 2017; Data ref: Krebs et al, 2017) (Figs. 3B and EV4B; Datasets EV3 and EV4). Taken together, these results confirm that the low-frequency footprints observed at mouse promoters are created by Pol II at the pausing site.

### A quantitative model to link molecular occupancy at core promoters with Pol II kinetics

**Figure 4:**
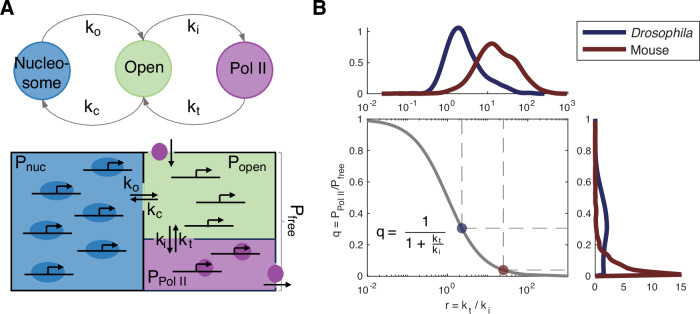
Kinetic modelling links Pol II occupancy with the initiation and Pol II turnover rates at promoters. (**A**) Schematic representation of promoter states derived from SMF experiments: “Nucleosome” indicates the promoter is bound by a nucleosome (blue), “Open” signifies that the promoter is unbound or bound only by PIC but not Pol II (green), and “Pol II” represents the promoter bound by Pol II (purple). Transitions between promoter states are described by four effective rates: nucleosome bound (*k*_*c*_), nucleosome unbound (*k*_*o*_), transcription initiation (*k*_*i*_), and Pol II turnover (*k*_*t*_). The probability of a promoter being in each state is a function of the rates (see Methods for details) and relates to the promoter frequencies observed by SMF. The probabilities of finding promoters in the Nucleosome (nucleosome state from SMF), Open (unbound and PIC states from SMF), and Pol II (PIC + Pol II and Pol II states from SMF) states are denoted as P_nuc_, P_open_, and P_Pol II_, respectively. (**B**) Our kinetic model links the relative Pol II occupancy (q), defined as the ratio of DNA molecules bound by Pol II with respect to DNA molecules free of nucleosome (q = P_Pol II_/P_free_), with the ratio of Pol II turnover rate (*k*_*t*_) to transcription initiation rate (*k*_*i*_) (see equation inside the figure). The relationship derived from the equation is depicted as a solid grey line. The *y* axis displays Pol II occupancies (q) as measured by genome-wide SMF experiments. The plot on the right shows the distribution of q on the data, highlighting a clear difference between *Drosophila* (blue) and mouse (red). The *x* axis presents the predicted ratio (r) of the Pol II turnover rate (*k*_*t*_) and the initiation rate (*k*_*i*_) for each promoter. The distributions of these predicted ratios are presented on the top, illustrating a clear difference between *Drosophila* (blue) and mouse (red). The dots represent median values of q and r for *Drosophila* (blue) and mouse (red).

We next aimed to identify the mechanisms explaining the interspecies differences in Pol II occupancy at promoters. Given the conservation of the general transcription machinery, we hypothesised that these differences might arise from variations in the kinetics of molecular processes, including the transcription initiation rate and Pol II turnover rate. Here, Pol II turnover rate is a combination of the rate at which Pol II undergoes premature termination and the rate at which Pol II progresses to elongation. To determine whether transcription initiation or Pol II turnover is more likely to explain the observed differences in Pol II occupancy, we developed a theoretical framework that relates promoter occupancy, either by nucleosomes or Pol II, to the effective rates of Pol II initiation and turnover. To do this, we implemented a stochastic model of transcription that explicitly considers three promoter states that can be captured by SMF: a closed state where the promoter is occupied by nucleosomes (P_nuc_); an unbound state consisting of accessible DNA without nucleosome or Pol II (P_open_); and a Pol II-bound state where the promoter is occupied by Pol II (P_Pol II_) (Fig. 4A). The transitions between these states are described by effective kinetic rates: nucleosome binding-unbinding (*k*_*c*_ and *k*_*o*_), transcription initiation (*k*_*i*_), and Pol II turnover (*k*_*t*_). Mechanistically the turnover rate (*k*_*t*_) is an effective rate that combines two distinct events that cannot be distinguished by our assay: Pol II entry into productive elongation or early transcription termination. This approach based on modelling SMF-derived states allows for a mechanistic interpretation of the kinetic parameters. This brings an improvement to existing stochastic models of transcription developed from imaging experiments where the kinetic parameters are not formally connected to precise molecular events (Peccoud and Ycart, 1995; Zoller et al, 2015).

Solving our model at a steady-state leads to simple expressions for the expected frequencies of each promoter state as a function of the kinetic rates (see “Methods” for details). In this simple model, the relative Pol II occupancy (q), defined as the ratio of DNA molecules bound by Pol II with respect to DNA molecules free of nucleosome, depends only on the ratio of the Pol II turnover rate (*k*_*t*_) and the initiation rate (*k*_*i*_) through a simple sigmoid function (Fig. 4B). This result can be intuitively understood by acknowledging that at steady-state there is no net flux of probability and detailed balance holds. Therefore, the rates of transitions between promoter states must balance. Specifically, the rate at which Pol II binds to open promoters equals the rate at which Pol II leaves the promoter: *k*_*i*_**·** P_open_ = *k*_*t*_**·** P_Pol II_, leading to the result r = *k*_*t*_/*k*_*i*_ = P_open_/P_Pol II_. Substituting this equation into the definition of q, i.e. q = P_Pol II_ / (P_open_ + P_Pol II_), we obtain the main theoretical result of this study. In other words, the Pol II occupancy is the result of the equilibrium between how fast the Pol II is loaded at the promoter and how fast it leaves (to either elongation or early termination). Importantly, by integrating the promoter state frequencies estimated from SMF data into our model, we can estimate the ratio between rates (*k*_*t*_/*k*_*i*_) for each promoter in the *Drosophila* and mouse genome (Fig. 4B). We observed a 7-fold difference in the median of this ratio between species (Fig. 4B). In summary, we build a theoretical model that allows us to relate changes in Pol II frequency with the kinetic rates of the different steps of the process.

### Faster Pol II turnover and lower initiation at mouse promoters

Our model predicts that the differences in Pol II occupancy between species could arise from a higher transcription initiation rate in *Drosophila* or a higher Pol II turnover rate in mouse; or a combination of both. Our model quantitatively links the occupancy as measured by SMF to the ratio of the initiation rate (*k*_*i*_) and the Pol II turnover rate (*k*_*t*_). Thus, there is an interdependence between these parameters, allowing us to predict one if the other one can be measured. For instance, we would be able to estimate the initiation rate if we can measure the turnover rate of Pol II at promoters.

**Figure 5:**
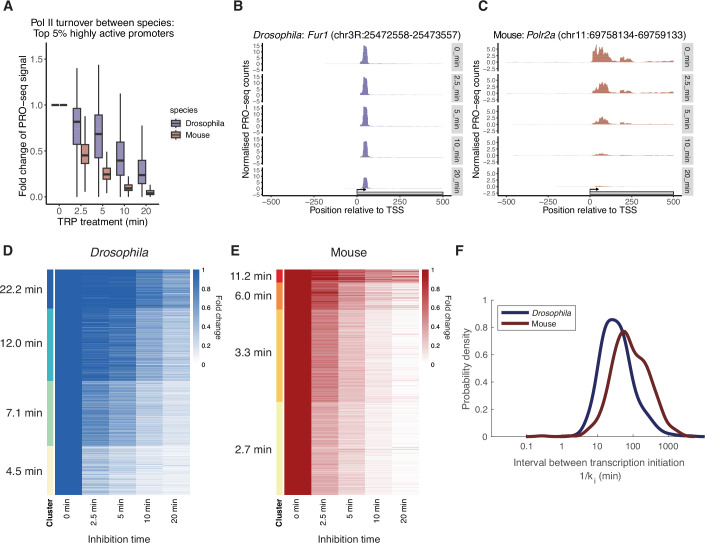
Mouse promoters exhibit a faster Pol II turnover rate and a lower initiation rate compared to Drosophila promoters. (**A**) Comparison of Pol II turnover following inhibition of transcription initiation via time-course triptolide (TRP) treatment between the top 5% highly active *Drosophila* (*n* = 930 promoters) and mouse promoters (*n* = 1254 promoters). Boxplots represent the distribution of PRO-seq signal at highly active promoters. PRO-seq reads were counted around the TSS [−100:200]. PRO-seq reads were then normalised for each sample using Spike-in read counts (see “Methods” for details). Each data point is an average of the two biological replicates, except in the 2.5 min TRP treatment, where only one replicate passed quality control. For each promoter, the fold change of normalised PRO-seq reads was calculated at each time point relative to the DMSO treatment (0 min). The *x* axis indicates the duration of TRP treatment, while the *y* axis represents the fold change of normalised PRO-seq counts compared to the DMSO treatment. The middle line of the box represents the median. The box displays the interquartile range (IQR), 25th to 75th percentile. Whiskers represent a distance of 1.5 × IQR. (**B**,** C**) Single-site example of time-course PRO-seq data following the time-course TRP treatment from (**B**) the mouse *Polr2a* promoter and (**C**) the *Drosophila Fur1* promoter, both with a similar initial Pol II binding frequency (19%). Normalised PRO-seq counts were smoothed over 25 bp. (**D**, **E**) K-means clustering of the fold changes in normalised PRO-seq counts at each time point relative to the DMSO treatment (0 min) for (**D**) the top 5% *Drosophila* promoters and (**E**) the top 5% mouse promoters. The half-life of Pol II for each cluster was determined by fitting an exponential decay model to the normalised PRO-seq counts and shown next to the heatmap. (**F**) Modelling of relative Pol II occupancy (q) and Pol II turnover rate (*k*_*t*_) reveals the difference in transcription initiation rate (*k*_*i*_) between mouse and *Drosophila*. The density plot shown with *x* axis represents interval between transcription initiation, which is the inverse of the initiation rate (1/*ki*).

To estimate Pol II turnover rates in each species, we treated *Drosophila* S2 cells and mESCs with TRP to inhibit transcription initiation and measured the change in promoter-proximal Pol II at multiple time points (0, 2.5, 5, 10, and 20 min) using PRO-seq (Judd et al, 2020) (see Methods for details). The PRO-seq intensity and their changes over time were highly correlated between the two replicates (Appendix Fig. S3). We found that the turnover of promoter Pol II following the inhibition of transcription initiation is faster in mouse than in *Drosophila* (Fig. 5A; Datasets EV5 and EV6). For example, although the mouse *Polr2a* promoter and *Drosophila Fur1* promoter have comparable Pol II binding frequencies measured by SMF (19%), the *Polr2a* promoter showed a faster decay of promoter-proximal Pol II than the *Fur1* promoter (Fig. 5B,C). To further investigate the differences in Pol II turnover between species, we clustered promoters according to the changes in promoter-proximal Pol II measured by PRO-seq over time (Fig. 5D,E; Datasets EV5 and EV6). We fitted an exponential decay model on each cluster to estimate the half-life of Pol II at promoters (Datasets EV7 and EV8). We observed that about 20% of *Drosophila* promoters have a half-life of less than 5 min (Fig. 5D). In contrast, more than 80% of mouse promoters have a half-life of less than 5 min, suggesting that Pol II turnover rate is generally faster at mouse promoters (Fig. 5E).

**Figure EV5:**
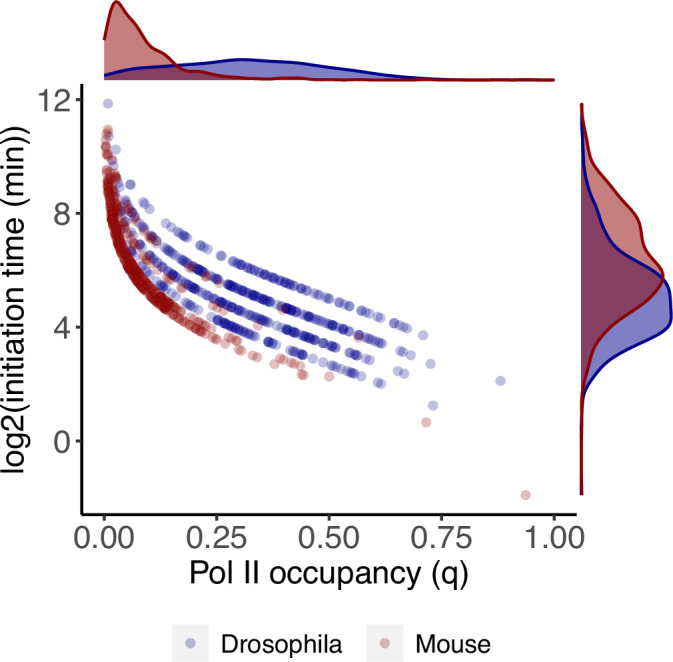
Distribution of Pol II occupancy (q) and initiation time at highly active *Drosophila* and mouse promoters. The x-axis displays Pol II occupancy (q), while y-axis represents log2(initiation time (min)). Each dot represents a promoter. Dot colours correspond to species (blue—*Drosophila*, red—mouse). The density plots on the top and on the right show the distribution of Pol II occupancy (q) and initiation time, respectively.

Having established that Pol II turnover rates differ between species, we then integrated these experimentally determined turnover rates into our model to ask if these are sufficient to explain the differences in Pol II occupancy observed between species. Using the interdependency between the initiation and the turnover rates, we predicted the initiation rates (*k*_*i*_) for each gene as a function of Pol II occupancy (q) and Pol II turnover (*k*_*t*_) (see Methods for details). If the turnover rates were sufficient to explain the differences in occupancy, the distribution of initiation rates should be similar between species. In contradiction with this hypothesis, we observed that the predicted initiation rates are significantly faster for *Drosophila* promoters (median = 0.032 min^−1^) than those of mouse promoters (median = 0.014 min^−1^) (Fig. 5F; Datasets EV7,EV8). This means that a Pol II molecule initiates on average every 32 min at *Drosophila* promoters, while it initiates every 72 min at mouse promoters. These slower initiation rates in mouse are consistent with the general notion of lower burst frequency in mammalian cell lines compared to *Drosophila* from live transcription imaging experiments (Lammers et al, 2020; Meeussen and Lenstra, 2024). Direct comparison of the distribution of these parameters between species shows that these are partially overlapping with a continuum of values between mouse and *Drosophila* (Fig. EV5), consistent with the idea that these represent different rates of the same molecular mechanism. Our results thus suggest that while Pol II turnover contributes to the differences in Pol II occupancy between species, it is not sufficient to entirely explain them. The remaining fraction of the differences could be explained by different rates of transcription initiation.

## Discussion

**Figure 6:**
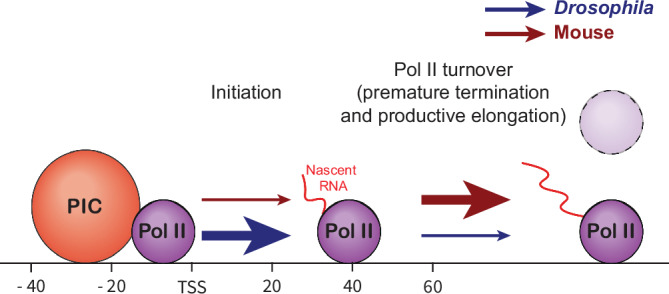
Summary of the differences in Pol II occupancy kinetics between mouse and *Drosophila* promoters. The lower Pol II occupancy at mouse promoters can be attributed to (1) a reduced transcription initiation rate and (2) an increased Pol II turnover, which includes both premature termination and productive elongation, in comparison to *Drosophila* promoters. The colours of the arrows represent mouse (red) and *Drosophila* (blue), with the size of the arrows indicating the rate of activity—smaller arrows represent lower rates, while larger arrows denote higher rates.

Here, we found that promoter-proximal Pol II pausing at mouse promoters is much less frequent than at *Drosophila* promoters. While up to 40% of the cells have Pol II engaged at active genes in *Drosophila*, this drops to <10% at most promoters of active genes in mouse cells. This difference in Pol II occupancy contrasts with the occupancy of other factors, such as the PIC, which shows comparable levels of occupancy at promoters in both species. Mechanistically, we show that these contrasts in occupancy reflect differences in the kinetics of recruitment and turnover of Pol II at the promoter. Mouse cells are characterised by faster turnover of Pol II and a lower initiation rate, which together reduce the number of Pol II molecules engaged in DNA at any given time (Fig. 6).

*Drosophila* and mouse cells are popular experimental models for studying transcription mechanisms. Pol II occupancy has been profiled with various genomics assays such as ChIP-seq, PRO-seq or sequencing of short capped transcripts (Kwak et al, 2013; Nechaev et al, 2010; Shao and Zeitlinger, 2017). It is thus surprising that these differences in Pol II occupancy were not detected earlier. This may arise from fundamental limitations of bulk assays to estimate global changes. Bulk assays are based on the experimental enrichment of the Pol II-associated molecules. In contrast, SMF measures all the DNA molecules of a cell population, regardless of their occupancy by Pol II. This fundamental difference in experimental principles provides a unique opportunity to quantify the frequency of factor occupancy on DNA, which cannot be estimated from bulk genomics data (Krebs, 2021; Krebs et al, 2017). This approach can, in principle, be expanded to any DNA-associated factors for which reference occupancy data exist (Kleinendorst et al, 2021).

Regulation of transcription after initiation through Pol II pausing has been proposed to be critical for the regulation of genes requiring rapid induction, such as those involved in key developmental transitions or stress responses (Core and Adelman, 2019; Fuda et al, 2009; Shao and Zeitlinger, 2017). The observation of the characteristic accumulation of Pol II downstream of the TSS led to the model that the rapid transcription response would be mediated through the release of these preloaded polymerases into the gene body (Adelman and Lis, 2012). Several lines of evidence from genomics and live-cell-imaging approaches argue that Pol II at the pause site rapidly turns over at a large fraction of genes (Darzacq et al, 2007; Erickson et al, 2018; Krebs et al, 2017; Nilson et al, 2017; Price, 2018; Steurer et al, 2018), suggesting an alternative model where paused genes frequently experience early transcription termination. Comprehensive measure of Pol II dynamics recently showed that promoter-proximal termination plays a key role in controlling dynamic transcriptional changes during transdifferentiation of human B-cells (Lysakovskaia et al, 2025), revealing that early termination is a prevalent mechanism in gene expression control. The identification and characterisation of early transcription termination by the Integrator complex provided a mechanism supporting this model (Elrod et al, 2019; Fianu et al, 2024; Razew et al, 2024; Wagner et al, 2023). Our data suggest that in mouse, Pol II is detected at the paused site in a small fraction of cells. Moreover, we found that Pol II turnover is faster at mouse than *Drosophila* promoters, with half-lives of less than 5 min in most highly active mouse promoters. These observed short Pol II half-lives in mouse cells are consistent with values observed human colon cancer cell line HCT116 (2–5 min).

This higher turnover rate in mouse cells could be the result of increased premature termination or faster entry into productive elongation. Our datasets do not distinguish between these two potential fates of Pol II and their relative contribution to the differences in Pol II occupancy. Several lines of existing evidence argue that a majority of the Pol II turnover at the paused site occurs through premature termination rather than productive elongation. For instance, chemical inhibition of elongation did not alter the amount and the rate at which Pol II turns over upon inhibition of initiation in S2 cells (Krebs et al, 2017) or human HCT116 cells (Erickson et al, 2018). In agreement with this notion, recent estimates shows that that ~80% of promoter-proximal Pol II molecules will be lost by premature termination (Zimmer et al, 2021). Thus, given the prevalence of premature termination, it is likely that it would contribute to the faster Pol II turnover we observed in mouse cells. Yet, these two hypotheses are not mutually exclusive, and higher elongation rates may also contribute to the observed differences.

By integrating experimental data into our quantitative model, we found that the Pol II turnover rate alone cannot fully account for the discrepancy in Pol II occupancy between mouse and *Drosophila*. We predict that the remaining variance is due to a lower transcription initiation rate at mouse promoters. This prediction is in agreement with the observation that transcriptional bursts at mammalian genes are interspersed with long periods of transcriptionally inactive states (Lammers et al, 2020; Meeussen and Lenstra, 2024; Stavreva et al, 2019; Suter et al, 2011; Wan et al, 2021). The presence of TATA-box has been associated with longer durations of the bursts and more frequent Pol II initiation (Larsson et al, 2019; Pimmett et al, 2021). Our data are in line with this notion, as Pol II occupancy is higher at TATA-containing promoters compared to TATA-less promoters in both mouse and *Drosophila*. Transcriptional bursting has been described by models such as the two-state telegraph model (Peccoud and Ycart, 1995; Raj et al, 2006; Tunnacliffe and Chubb, 2020), as well as more intricate models that accommodate complex molecular mechanisms underlying transcription, including Pol II pausing (Pimmett et al, 2021; Tantale et al, 2021). A limitation of these models is that they provide only phenomenological descriptions of the process, but the underlying molecular mechanisms are not explicitly identified. Here, we show that single-molecule assays, such as SMF used in this study, can be used to formally link occupancies of factors like nucleosomes, PIC, and Pol II at *cis*-regulatory elements with kinetics of regulatory processes. Similar principles have recently been applied to relate TF binding with promoter state and transcription at synthetic *cis*-regulatory elements (Doughty et al, 2024), demonstrating the potential of the approach to connect the molecular states of regulatory elements with transcriptional kinetics.

Together, our study provides new insights into the kinetics of promoter-proximal Pol II occupancy with implications in our understanding of the transcription regulation mechanism. Moreover, it proposes a framework for quantitative integration of the occupancy patterns of regulatory factors across the cells of a population into kinetic models.

## Methods

**Table:** Reagents and tools table

Reagent/resource	Reference or source	Identifier or catalogue number
**Experimental models**		
159 DNMT TKO (*Mus musculus*)	Domcke et al, 2015	
S2 (*Drosophila melanogaster*)	Wirbelauer et al, 2005	
**Recombinant DNA**		
Not applicable		
**Antibodies**		
Not applicable		
**Oligonucleotides and other sequence-based reagents**		
PCR primers for amplicon	This study	Table EV1
Primers for PRO-seq library preparation	Judd et al, 2020	
**Chemicals, enzymes, and other reagents**		
Dulbecco’s Modified Eagle Medium, high glucose	Gibco	41965039
Schneider’s *Drosophila* medium	Gibco	21720024
FBS	Sigma	F7524
Gelatin	Sigma	G-1890
MEM Non-Essential Amino Acids Solution	Gibco	11140050
Sodium pyruvate	Gibco	11360070
L-glutamine	Gibco	A2916801
2-Mercaptoethanol	Sigma	M6250
LIF	EMBL PEPCORE	
DMSO	Sigma	D8418
Proteinase K	Sigma-Aldrich	124568
Trypsin-EDTA (0.25%)	Gibco	25200056
Trypan blue solution (0.4% (wt/vol))	Gibco	15250061
Nuclease-Free Water (not DEPC-Treated)	Ambion	AM9937
Trizma base	Sigma-Aldrich	T1503
Sodium chloride (NaCl)	Sigma-Aldrich	S7653
Titriplex III (ethylenedinitrilotetraacetic acid disodium salt dihydrate) (EDTA)	Sigma	1.08421
Magnesium chloride (MgCl2)	Sigma	M8266
IGEPAL CA-630	Sigma	I8896
Sodium dodecyl sulfate solution 10% (SDS)	Sigma-Aldrich	71736
GpC Methyltransferase (M.CviPI)	New England Biolabs	M0227L
CpG Methyltransferase (SssI)	New England Biolabs	M0226L
S-adenosyl-methionine (SAM) 32 mM	New England Biolabs	B9003S
RNase A, DNase- and protease-free (10 mg/ml)	Sigma	R6513
Agencourt AMPureXP magnetic beads	Beckman Coulter	A63880
Phenol equilibrated, stabilised: Chloroform: Isoamyl Alcohol 25: 24: 1	PanReacAppliChem	A0889
Chloroform	Sigma	366919
2-propanol	Sigma	I9516
Glycogen from Mytilus edulis (Blue mussel)	Sigma-Aldrich	G1767
Ethanol	Sigma	1.00983
KAPA HiFi HotStart Uracil+ ReadyMix (2X)	Roche	KK2802 07959079001
Agarose	Sigma	A9539
Ethidium bromide solution 1%	Roth	2218.1
GeneRuler 1 kb DNA Ladder, ready-to-use	Thermo	SM0313
GeneRuler 100 bp DNA Ladder, ready-to-use	Thermo	SM0244
ThermoPol Reaction Buffer Pack - 6.0 ml	New England Biolabs	B9004S
TRIzol Reagent	Thermo Fisher	15596026
T4 RNA Ligase 1 (ssRNA Ligase) - 5,000 units	New England Biolabs	M0204L
EGTA	Sigma-Aldrich	E3889
RNA 5’ Pyrophosphohydrolase (RppH) - 200 units	New England Biolabs	M0356S
Superase-In RNase Inhibitor	Thermo Fisher	
Q5 High-Fidelity DNA Polymerase—100 units	New England Biolabs	M0491S
Sarkosyl	Sigma-Aldrich	61739
SYBR Gold Nucleic Acid Gel Stain	Thermo Fisher	S-11494
Sucrose	Sigma-Aldrich	S7903
Sodium hydroxide	Sigma-Aldrich	S8045
Potassium Chloride	Sigma-Aldrich	P9541
Glycerol	Thermo Fisher Scientific	17904
Dynabeads MyOne Streptavidin C1 Invitrogen -	Thermo Fisher	65001
Dithiothreitol (DTT)	Thermo Fisher Scientific	707265 ML
T4 Polynucleotide Kinase	New England Biolabs	M0201S
Triton X-100	Fisher Scientific	BP151-100
Tween-20	Sigma-Aldrich	P9416
ATP	Thermo Fisher	18330019
GTP	Thermo Fisher	18332015
Pierce Protease Inhibitor	Thermo Fisher	A32963
GlycoBlue™ Coprecipitant (15 mg/mL)	Thermo Fisher	AM9515
Maxima H Minus Reverse Transcriptase (200 U/uL)	Thermo Fisher	EP0753
dNTP mix	Thermo Fisher	R1121
Biotin-11-CTP	Perkin Elmer	NEL542001EA
Biotin-11-UTP	Perkin Elmer	NEL543001EA
**Software**		
R (version 4.2.2)	R Core Team, 2022	
SingleMoleculeFootprinting (‘promoter’ branch)	https://github.com/Krebslabrep/SingleMoleculeFootprinting/tree/promoter	
QuasR (version 1.38.0)	https://bioconductor.org/packages/release/bioc/html/QuasR.html	
Trim-Galore! (version 0.6.7)	https://github.com/FelixKrueger/TrimGalore	
Cutadapt (version 3.5)	https://cutadapt.readthedocs.io/en/stable/	
Picard (version 2.15.0)	https://broadinstitute.github.io/picard/	
UMI-tools (version 1.1.2)	https://github.com/CGATOxford/UMI-tools	
Adobe Illustrator	https://www.adobe.com/products/illustrator.html	
**Other**		
Qubit dsDNA HS Assay Kit	Life Technologies	Q32851
NextSeq 2000	Illumina	
Epitect bisulfite conversion kit	Qiagen	59104
NEBNext Ultra II DNA Library Prep Kit for Illumina	NEB	E7645L
NEBNext Multiplex Oligos for Illumina (Index Primers 1-12)	NEB	E7335L
SureSelectXT Methyl-Seq Reagent Kit	Agilent	G9651A
SureSelectXT Mouse Methyl-Seq Capture Library	Agilent	931052
EZ DNA-methylation gold kit	Zymo research	D5005
Total RNA Purification Kit	Norgen Biotek Corp.	37500

### Cell culture and treatment

DNA methyltransferase triple knockout mouse embryonic stem cells (TKO mESCs) (Domcke et al, 2015) were cultured at 37 °C on 10-cm plates coated with 0.2% gelatin. The cells were maintained in Dulbecco’s Modified Eagle Medium (DMEM, Thermo Fisher) supplemented with 15% foetal bovine serum (FBS, Sigma-Aldrich), 1% sodium pyruvate (Sigma-Aldrich), 1 mM l-glutamine, 1× non-essential amino acids (NEAA, Thermo Fisher), 0.2% leukaemia inhibitory factor (LIF, produced in-house), and 0.01% β-mercaptoethanol. Media were regularly replaced, and cells were passaged as needed to maintain optimal growth conditions.

Schneider-S2 cells were grown at 25 °C in Schneider’s *Drosophila* medium (LifeTech: 21720-001) supplemented with 10% FBS.

To inhibit transcription initiation, TKO mESCs were treated with 500 nM triptolide (TRP) (Sigma) for 30 min. Control cells were treated with an equal volume of DMSO. Following treatment, both control and TRP-treated cells were prepared for amplicon Single-Molecule Footprinting (SMF).

For the time-course TRP experiment, TKO mESCs and S2 cells were treated with 10 µM TRP for varying durations (2.5, 5, 10, and 20 min). Control cells were treated with an equivalent volume of DMSO for 20 min. After treatment, cells were harvested for PRO-seq. No blinding was performed in this study. The cells used in this study were not recently authenticated or tested for mycoplasma contamination.

### Single-molecule footprinting

Dual-enzyme SMF was performed following the protocol from (Kleinendorst et al, 2021; Sönmezer et al, 2021). Briefly, cells were collected by trypsinization, washed in ice-cold PBS and counted with a trypan blue based automatic counter (Bio-Rad). Then, per SMF reaction, 250,000 cells were lysed in chilled lysis buffer (10 mM Tris (pH = 7.4), 10 mM NaCl, 3 mM MgCl2, 0.1 mM EDTA, 0.5% NP40) for 10 min on ice. Nuclei were pelleted by centrifuging 5 min at 4 °C and 1000 × *g*, and washed in wash buffer (10 mM Tris (pH = 7.4), 10 mM NaCl, 3 mM MgCl2, 0.1 mM EDTA). Next, nuclei were resuspended in 1x M.CviPI reaction buffer (50 mM Tris (pH 8.5), 50 mM NaCl, 10 mM DTT and 300 mM sucrose) and incubated for 7.5 min at 37 °C with 200 U of M.CviPI (NEB-M0227L) and 0.6 mM SAM (NEB). A second 7.5 min incubation was performed with an additional 100U of M.CviPI and 0.128 mM SAM. Next, 10 mM MgCl_2_, 0.128 mM SAM and 60U of M.SssI (NEB-M0226L) were added, and nuclei were incubated in a third round of 7.5 min at 37 °C. The methylation reaction was stopped with stop buffer (20 mM Tris, 600 mM NaCl, 1% SDS 10 mM EDTA). All samples were digested with Proteinase K (200 mg/ml) overnight at 55 °C, followed by phenol/chloroform purification and isopropanol precipitation of DNA.

### Amplicon SMF

A set of 62 mouse promoters of protein-coding genes were targeted with amplicon SMF. These promoters were selected to cover promoters with and without a TATA-box, covering a variety of expression levels (by RNA-seq) and activity levels (Pol II ChIP at the promoter). Bisulfite-specific primers were designed using a custom R script based on using Primer3 with slight modifications, designing against in silico bisulfite-converted sequences excluding CpG and GpC dinucleotides. For some promoters, two primer-pairs were designed for control purposes (Table EV1). Additionally, the primer plate covered 8 tRNA promoters (not analysed here) and re-used 12 primer-pairs used before for control purposes: 6 covering promoters, 6 covering TF binding sites (Sönmezer et al, 2021).

Amplicon SMF was performed as previously described (Kleinendorst et al, 2021; Sönmezer et al, 2021). Briefly, the isolated footprinted DNA (~ 1 μg per sample) was bisulfite converted with the Epitect bisulfite conversion kit (QIAGEN), according to the manufacturer’s instructions. The converted DNA was distributed over a 96-well plate, and PCR-amplified in a total volume of 16 μL with 1× KAPA HiFi HotStart Uracil+ ReadyMix (Roche) and 625 nM primers (one primer-pair, forward and reverse combined), using the following cycling protocol: 3 min at 95 °C, 35 cycles of (20 s at 98 °C, 30 s at 56 °C, 60 s at 72 °C), 5 min at 72 °C. PCR product was checked by gel electrophoresis, 10 μL per reaction was pooled and purified using 0.8× AMPureXP bead purification (Beckman Coulter). In all, 1 μg of purified DNA per sample was used to prepare sequencing libraries, using the NEBNext Ultra II Kit according to manufacturer’s protocol and using NEBNext Multiplex Oligos to multiplex up to 12 libraries. Libraries were sequenced on a MiSeq instrument to generate 250 bp paired-end reads.

### PRO-seq

Precision run-on sequencing (PRO-seq) was performed as previously described (Judd et al, 2020; Kwak et al, 2013) with slight modifications. For the *Drosophila* S2 cell samples, 10 million *Drosophila* S2 cells were used per sample with a spike-in of 0.1 million (1%) TKO mESCs. For the TKO mESC samples, 5 million TKO mESCs were used per sample with a spike-in of 0.05 million (1%) *Drosophila* S2 cells. Two replicates per TRP treatment time point were generated, with each replicate prepared simultaneously for all cell lines and conditions. Cells were harvested using Trypsin-EDTA on ice and washed twice with 10 mL ice-cold PBS, followed by centrifugation at 1000 × *g* for 4 min at 4 °C. Permeabilisation was performed in 10 mL of permeabilisation buffer (10 mM Tris-HCl pH 8.0, 250 mM sucrose, 10 mM KCl, 5 mM MgCl2, 1 mM EGTA, 0.1% Igepal, 0.05% Tween-20, 0.5 mM DTT, 10% (vol/vol) glycerol, 1 tablet of protease inhibitor per 50 mL, 4 units/ml SUPERaseIN inhibitor) on ice for 10 min. The cell pellets were then washed twice with 10 mL of ice-cold cell wash buffer (10 mM Tris-HCl pH 8.0, 250 mM sucrose, 10 mM KCl, 5 mM MgCl2, 1 mM EGTA, 0.5 mM DTT, 10% (vol/vol) glycerol, 1 tablet of protease inhibitor per 50 mL, 4 units/mL SUPERaseIN inhibitor) with centrifugation at 1000x g for 4 min at 4 °C. Cells were resuspended in 50 μL freeze buffer (50 mM Tris-HCl, pH 8.0, 40% (vol/vol) glycerol, 5 mM MgCl2, 1.1 mM EDTA, 0.5 mM DTT and 4 units/mL SUPERaseIN inhibitor), snap-frozen, and stored at −80 °C until further use. The nuclear run-on reaction was performed as a 2-biotin run-on. For this, 50 μL of the 2× Nuclear-run on master mix (10 mM Tris-Cl pH 8.0, 5 mM MgCl_2_, 1 mM DTT, 300 mM KCl, 40 μM Biotin-11-UTP, 40 μM Biotin-11-CTP, 40 μM ATP, 40 μM GTP, 1% Sarkosyl, 1 μL SUPERaseIN inhibitor) was prepared for each sample. The reaction mix was pre-heated at 37 °C (30 °C for *Drosophila* S2 cell samples), and 50 μL of the pre-calculated number of cells were added to each reaction vial, mixed thoroughly and incubated at 37 °C (30 °C for *Drosophila* S2 cell samples) for 5 min with shaking (750 rpm). To stop the reaction, 350 μL of the RL Buffer from the Norgen RNA extraction kit were added and vortexed. In total, 240 μL of 100% ethanol was added to the mixture and vortexed again. RNA extraction was performed according to the kit’s manual. The final RNA was eluted twice with 50 μL H2O and pooled to a final volume of 100 μL. For the base hydrolysis, the RNA was denatured for 30 s at 65 °C and snap-cooled on ice. In all, 25 μL of ice-cold 1 N NaOH were added and incubated on ice for 10 min. RNA was precipitated by adding 125 μL Tris-HCl (pH 6.8), 5 μL NaCl, 1 μL GlycoBlue and 650 μL 100% EtOH and centrifugation at 20,000 × *g* at 4 °C for 20 min. The RNA pellet was washed with 70% EtOH, air-dried and resuspended in 6 μL H_2_O. For the 3’ RNA adaptor ligation, 1 μL of REV3 3’RNA adaptor dilution (10 μM), heat-denatured at 65 °C for 20 s and placed on ice, was added to the 6 μL resuspended RNA. 13 μL of the RNA ligation mix using T4 RNA ligase I was added and incubated at 25 °C for 1 h. Biotin RNA enrichment was performed by adding 55 μL binding buffer (10 mM Tris-HCL pH 7.4, 300 mM NaCl, 0.1% Tergitol, 1 mM EDTA) to each ligation reaction, followed by 25 μL of pre-washed streptavidin beads. Reactions were incubated on a rotator set to 8 rpm at room temperature for 20 min. Beads were washed once with ice-cold high-salt buffer (50 mM Tris-HCL pH 7.4, 2 M NaCl, 0.5% Tergitol, 1 mM EDTA), transferred to new tubes, and washed once with low-salt buffer (5 mM Tris-HCL pH 7.4, 0.1% Tergitol, 1 mM EDTA). On-bead 5’ hydroxyl repair was performed by resuspending the beads in 19 μL of the PNK mix and incubated at 37 °C for 30 min with soft shaking (350 rpm). For the 5’ cap repair reaction, the beads were resuspended in the enzyme mix containing RppH and ThermoPol Reaction Buffer and incubated at 37 °C for 1 h with soft shaking (350 rpm). On-bead 5’ RNA adaptor ligation was performed by resuspending the beads in 7 μL of REV5 5’RNA adaptor dilution (6 μL of H_2_O + 1 μL of 10 μM REV5), heat-denatured at 65 °C for 30 s and placed on ice. 12 μL of the RNA ligation mix using T4 RNA ligase was added and incubated at 25 °C for 1 h. Beads were washed once with ice-cold high-salt buffer, transferred to new tubes, and washed once with low-salt buffer. The RNA was cleaned-up using Trizol and chloroform. The RNA was reverse transcribed using the Maxima H minus RT enzyme and the RP1 reverse-transcription primer. The correct number of PCR cycles was determined by test PCR and Bioanalyzer analysis. In the end, the final PCR was performed using 13 cycles and the final library was cleaned-up using magnetic SPRI beads at a ratio of 1.8× and further size selected with a SPRI beads ratio of 1× to remove primer dimers. The library was run on a NextSeq 2000 P3 with 50 bp single-end sequencing.

### Sequencing data pre-processing

A list of sequencing data used in this study is shown in the Table EV2. SMF data was processed as previously described (Kleinendorst et al, 2021; Sönmezer et al, 2021). Briefly, raw paired-end sequencing reads were pre-processed using Trim-Galore! (version 0.6.7) (Krueger et al, 2021) to remove unpaired and low-quality reads, trim Illumina adaptor sequences, and trim low-quality bases. The whole-genome *Drosophila* SMF data were pre-processed similarly using Trim-Galore!, trimming one extra base from the start of each read to allow proper alignment of short fragments. The trimmed reads were aligned against a bisulfite index of the *Mus musculus* (mm10) or *Drosophila melanogaster* (dm6) genome using the R package QuasR (version 1.28.0) (Gaidatzis et al, 2015), which uses Bowtie (Langmead et al, 2009) as an aligner, with specific alignment parameters (alignmentParameter = -e 70 -X 1000 -k 2 –best –strata) and keeping only uniquely aligned reads. Duplicated reads were removed for genome-wide and bait-capture, but not for amplicon SMF data, using the tool MarkDuplicates from Picard (version 2.15.0) (Broad Institute, 2019).

For PRO-seq data generated in this study, raw sequencing reads were trimmed using Cutadapt (version 3.5) (Martin, 2011). The UMIs were detected and extracted using UMI-tools (version 1.1.2) (Smith et al, 2017). Reads were aligned to a normal index of the respective genome of the sample and spike-in (mm10 and dm6 for TKO mESC samples and *Drosophila* S2 cell samples, respectively) using QuasR with default alignment parameters. Aligned reads were deduplicated using UMI-tools. Reads aligned to rRNAs were removed.

Other publicly available datasets (ChIP-seq, RNA-seq, MNase-seq, PRO-seq) were trimmed using Trim-Galore and aligned using QuasR against a normal index of the respective genome (mm10 or dm6) with default alignment parameters.

### SMF—single-molecule methylation call

SMF data analysis was performed using the R package SingleMoleculeFootprinting (‘promoter’ branch) (Kleinendorst et al, 2021). Methylation was called on all cytosines in aligned reads with a minimum bisulfite conversion rate of 80%, using the CallContextMethylation function, which was built on the QuasR function qMeth (Gaidatzis et al, 2015). For single-enzyme SMF (Fig. EV1A,B), only Cs in the DGCHN (D = no C, H = no G, *N* = any base) were considered. For dual-enzyme SMF (Figs. 1–3 and EV1C,D), all methylation information from CpG and GpC contexts were used.

### SMF—single-molecule sorting

For single-molecule analysis, the molecular classifier from our previous study (Krebs et al, 2017) was adapted to adequately assess the presence of promoter-proximal footprints at mouse promoters. In brief, four bins of interest were defined relative to the TSS, based on the expected position of promoter elements where transcription machinery binds at the core promoter, including the PIC at the TATA site and Pol II at the Pol II pausing site (Cianfrocco et al, 2013; Lee et al, 2008). We next included the upstream and the downstream bins to better separate PIC and Pol II footprint from a broader and more continuous nucleosome footprints, under the assumption that promoters should be “accessible” or “nucleosome-free” during active transcription period. In total, the four bins include: upstream [-58:-43], TATA-box [-36:-22], Pol II [28:47], and downstream [54:69] (Fig. EV2A). For each TSS, all reads covering at least one relevant C per bin in all four bins were analysed. Thus, the analysis excluded any TSSs where any bin did not contain at least one relevant C. For each read, the average methylation per bin was calculated and rounded to 0 or 1, creating a 4 bits vector classifying the state of every read among 2^4^ = 16 theoretical possibilities (Fig. EV2B). The reads were grouped by the methylation pattern within four bins into six promoter states: Pol II, PIC + Pol II, PIC, unbound, nucleosome, and unassigned (Fig. EV2B). For whole-genome *Drosophila* S2 cells and bait-capture TKO mESCs, the frequencies of promoter states were calculated per replicate for each TSS with at least 20 sorted reads. The promoter state frequencies were then averaged across the replicates. For amplicon SMF, only TSSs with at least 100 sorted reads (47 promoters) were retained for the analysis. The frequencies of promoter states were calculated per replicate and then averaged across the replicates. For the analysis in Figs. 2E,F and 3B, the frequencies of Pol II and PIC + Pol II were combined as a Pol II binding frequency. Similarly, the frequencies of PIC and PIC + Pol II were combined as a PIC binding frequency.

### SMF—Plots

For composite plots (Figs. 1B–D and EV1), the average SMF (1 - % methylation) is displayed per C as a dot, along with the average over a 20 bp window as a line. In Fig. 1E,F, only the average line is displayed.

Functions from the R package SingleMoleculeFootprinting (‘promoter’ branch) (Kleinendorst et al, 2021) were used for generating single locus plots (Figs. 2B,D, 3A and EV4).

### Annotation of TSSs and promoters

TSSs were defined by taking the starting positions of all autosomal RefSeq transcripts (mm10 and dm6). Next, these TSSs were refined by shifting them to the strongest CAGE-seq peak within 50 bp of the TSS on the same strand as the gene (for mouse: CAGE peaks from the FANTOM5 project (The FANTOM Consortium and the RIKEN PMI and CLST (DGT), 2014), lifted over to mm10 and reannotated by (Abugessaisa et al, 2017); for *Drosophila*: CAGE peaks from the modENCODE project (The modENCODE Consortium et al, 2010), lifted over to dm6 by us). For TSSs without a corresponding CAGE-seq peak, the original TSSs were retained. After this step, duplicated start sites were removed. The presence of a TATA motif was determined by searching for the consensus motif (TATAWAWR) in its theoretical position relative to the TSS ± 5 bp [−37:−18]. Promoter activity (e.g. top 5% highly active) was ranked according to Pol II ChIP-seq data (see below).

### Comparison with external datasets

For Pol II ChIP-seq, the start of each read was shifted 75 bp downstream to account for the average fragment size. The amount of Pol II was determined by counting the reads in a window of [-200:100] around each TSS, using the QuasR function qCount (Gaidatzis et al, 2015).

For RNA-seq, RPKM values were calculated per RefSeq transcript and assigned to the corresponding TSS.

Because PRO-seq is strand-specific and the nascent RNA is sequenced from the 3’ end, only reads aligning to the strand opposite the gene were assessed. The amount of Pol II was determined by counting the reads in a window of [−200:100] around each TSS.

For the mouse MNase-seq data, the start of each read was shifted 80 bp downstream to account for the average fragment size. For the paired-end *Drosophila* MNase-seq data, the centre of each mapped fragment was used. The number of mapped reads was counted in a window of [−40:30] around the TSS to include only the nucleosomes overlapping the promoter and to exclude the −1 and +1 nucleosomes.

The positions of nucleosomes and Pol II relative to the TSS (Fig. 1E,F) were determined using the QuasR function qProfile on MNase-seq and PRO-seq data, respectively. In the window of [−250:250] around each TSS, read counts were normalised to reads per million mapped reads (RPM) and smoothed over a 20 bp window. RPM values at each position around the TSS were averaged across the top 5% highly active promoters (Fig. 1E) or the top 5% highly active, TATA-containing promoters (Fig. 1F). To enable a comparison of MNase-seq and PRO-seq data from two different species in the same plots, the average RPM for PRO-seq and MNase-seq at each position around TSS was normalised to the Z-score.

The global relationship between SMF-derived promoter states, Pol II ChIP-seq, RNA-seq, PRO-seq, and MNase-seq were assessed using Spearman correlation (Figs. EV2D and EV3B).

### PRO-seq analysis

PRO-seq data in this study were generated from the time-course TRP treatment experiment (see above for details). Two samples were dropped (one replicate of TKO mESCs at 2.5 min and one replicate of *Drosophila* S2 cells at 2.5 min) and as they did not pass the quality control.

After alignment against the respective genomes (mm10 or dm6), PRO-seq reads were counted in a window around TSSs [-100:200] using the QuasR function qCount (Gaidatzis et al, 2015). Because PRO-seq is strand-specific and the nascent RNA is sequenced from the 3’ end, only reads aligning to the strand opposite the gene were assessed. For the correlation analysis between replicates (Appendix Fig. S3), PRO-seq counts at the TSSs were normalised to RPM and compared. Under the assumption that read counts from spike-ins should be constant across samples, spike-in reads were used to perform inter-sample normalisation and enable quantification of the global effects expected to occur upon inhibition of transcription (Fig. 5). For TKO mESC samples, PRO-seq read counts from each sample were normalised by the total reads mapped against the dm6 genome within the same sample. Similarly, for *Drosophila* S2 samples, PRO-seq read counts from each sample were normalised by the total reads mapped against the mm10 genome within the same sample. Replicates were averaged, and fold changes compared to the DMSO control were calculated (Fig. 5). The analysis was restricted to highly active genes (top 5% Pol II ChIP-seq as defined above).

To determine the Pol II turnover rate (Fig. 5D,E), fold changes of PRO-seq signal at top 5% highly active promoters were clustered using k-means clustering (k = 4). For each cluster, an exponential decay model was fitted to the normalised PRO-seq counts, assuming a Poissonian noise model. Turnover rates (k_*t*_) were estimated by maximising the likelihood, and confidence intervals were approximated by calculating the Hessian at the maximum. Half-lives were then calculated using the following expression τ_1/2_ = log(2)/k_*t*_. This analysis revealed the half-life of promoter-proximal Pol II for each gene cluster.

### Pausing index calculation

Read counts were collected in PRO-seq data without TRP treatment (0 min) around the TSS [−150:150] and in the gene body [+300:600] for each gene. Genes that are shorter than 600 bp were excluded from the analysis. Pausing index was then calculated as the ratio of Pol II at the TSS over Pol II at the gene body. Only promoters with finite pausing index values were included in the analysis.

### Mathematical model linking promoter occupancy state with transcriptional bursting

The simplest stochastic model widely used to describe transcriptional bursting is the so-called telegraph model (Peccoud and Ycart, 1995; Raj et al, 2006; Suter et al, 2011), in which the gene can exist in two states, ‘ON’ and ‘OFF,’ with transcription occurring at a certain initiation rate in the ‘ON’ state. More intricate models have been proposed to accommodate the complex stochastic dynamics observed experimentally, often relying on a predefined number of gene states and a matrix of rate transitions between them (Tantale et al, 2016; Zoller et al, 2015). However, the molecular and mechanistic interpretation of these gene states and their transitions is not always evident. In contrast, the SMF approach allows us to propose a stochastic model that explicitly considers the observed promoter states with specific molecular interpretation. For simplicity, we model only the three most important promoter states identified from SMF data: a nucleosome state, where the promoter is closed and occupied by nucleosomes; an open state, consisting of accessible DNA without detectable nucleosomes and Pol II footprints (Unbound and PIC states from SMF); and a Pol II-bound state, where the promoter is occupied by Pol II (PIC + Pol II and Pol II states from SMF). The rate transitions between these states acquire an immediate mechanistic interpretation: the transition between the closed and open states is determined by the effective rates of nucleosome binding and unbinding (*k*_*c*_ and *k*_*o*_), and the transition between the open and Pol II-bound states is determined by the effective Pol II initiation and turnover rates (*k*_*i*_ and *k*_*t*_). Thus, the probabilities of finding the promoter in a given state follow the master equation below:

$$\frac{d{P}_{{{\mathrm{nuc}}}}}{{dt}} = \,	 {k}_{c}{P}_{{{\mathrm{open}}}}-{k}_{o}{P}_{{{\mathrm{nuc}}}}\\ \frac{d{P}_{{{\mathrm{open}}}}}{{dt}} = \,	 {k}_{o}{P}_{{{\mathrm{nuc}}}}+{k}_{t}{P}_{{{{\mathrm{Pol}}}}\; {{{\mathrm{II}}}}}-({k}_{c}{+{k}_{i}})P_{{{\mathrm{open}}}}\\ \frac{d{P}_{{{{\mathrm{Pol}}}}\; {{{\mathrm{II}}}}}}{{dt}} = \,	 {k}_{i}{P}_{{{\mathrm{open}}}}-{k}_{t}{P}_{{{{\mathrm{Pol}}}}\; {{{\mathrm{II}}}}}$$

At steady-state, the probabilities of finding the promoter in an open state or bound by a Pol II molecule are:

$${P}_{{{\mathrm{open}}}}=\frac{{k}_{t}}{{k}_{t}+{k}_{i}}{P}_{{{\mathrm{free}}}}\quad {P}_{{{{\mathrm{Pol}}}}\; {{{\mathrm{II}}}}}=\frac{{k}_{i}}{{k}_{t}+{k}_{i}}{P}_{{{\mathrm{free}}}}$$

where we define the probability of the promoter being free as ${P}_{{{{\rm{f}}}}{{{\rm{r}}}}{{{\rm{e}}}}{{{\rm{e}}}}}={P}_{{{{\rm{o}}}}{{{\rm{p}}}}{{{\rm{e}}}}{{{\rm{n}}}}}\,+\,{P}_{{{{\rm{P}}}}{{{\rm{o}}}}{{{\rm{l}}}}{{{\rm{I}}}}{{{\rm{I}}}}}=1-{P}_{{{{\rm{n}}}}{{{\rm{u}}}}{{{\rm{c}}}}}.$ By rearranging the second equation above, we arrive at the main theoretical result of this paper:

$$q=\frac{{P}_{{{{\mathrm{Pol}}}}\; {{{\mathrm{II}}}}}}{{P}_{{{\mathrm{free}}}}}=\frac{1}{1+{k}_{t}/{k}_{i}}$$

showing that the fraction of promoter molecules occupied by Pol II over the fraction accessible molecules (q) follows a simple sigmoid function that depends on the ratio of the turnover rate *k*_*t*_ and the initiation rate *k*_*i*_. In other words, the Pol II occupancy level is determined by the balance of incoming Pol II complexes into the promoter and the Pol II turnover speed.

The same result can be obtained in a simpler and more straightforward manner by acknowledging that detailed balance in our system holds at steady-state. Therefore, the rate at which Pol II binds to open promoters equals the rate at which Pol II leaves the promoter:

$${k}_{i}\,{P}_{{\mbox{open}}}={k}_{t}\,{P}_{{{\mbox{Pol}}}\, {{\mbox{II}}}}$$

Rearranging this equation gives:

$$r=\,\frac{{k}_{t}}{{k}_{i}}\,=\frac{{P}_{{\mbox{open}}}}{{P}_{{{\mbox{Pol}}} \, {{\mbox{II}}}}}$$

and substituting this expression into the definition of relative Pol II occupancy *q* (see equation above), it leads to the sigmoid relationship between *r* and *q*:

$$q=\frac{{P}_{{{{\rm{P}}}}{{{\rm{o}}}}{{{\rm{l}}}}\,{{{\rm{I}}}}{{{\rm{I}}}}}}{{P}_{{{{\rm{P}}}}{{{\rm{o}}}}{{{\rm{l}}}}\,{{{\rm{I}}}}{{{\rm{I}}}}}+{P}_{{{{\rm{o}}}}{{{\rm{p}}}}{{{\rm{e}}}}{{{\rm{n}}}}}}=\frac{1}{1+{P}_{{{{\rm{o}}}}{{{\rm{p}}}}{{{\rm{e}}}}{{{\rm{n}}}}}/{P}_{{{{\rm{P}}}}{{{\rm{o}}}}{{{\rm{l}}}}\,{{{\rm{I}}}}{{{\rm{I}}}}}}=\frac{1}{1+{k}_{t}/{k}_{i}}=\frac{1}{1+r}$$

Note that our result is valid only under steady-state conditions. In situations where the system is not in a steady-state, for instance, when transcription is activated or deactivated upon external induction, it may be necessary to solve the master equation to obtain the full time-dependent solution.

### Statistical analysis and visualisation

All statistical analyses were carried out using R software (version 4.2.2) (R Core Team, 2022) and the R package *tidyverse* (version 2.0.0) (Wickham et al, 2019) and are described in the figure legends and “Methods”.

Plots were generated using *ggplot2* (version 3.4.4) (Wickham, 2016), *ggpubr* (version 0.6.0) (Kassambara, 2023), *cowplot* (version 1.1.1) (Wilke, 2020), and *pheatmap* (version 1.0.12) (Kolde, 2018). The upper and lower boundaries of the boxplots represent the 25th and the 75th percentiles. The central line represents the median.

## Supplementary information

Appendix

Table EV1

Table EV2

Peer Review File

Dataset EV1

Dataset EV2

Dataset EV3

Dataset EV4

Dataset EV5

Dataset EV6

Dataset EV7

Dataset EV8

Expanded View Figures

## Data Availability

The datasets and computer code produced in this study are available in the following databases: Amplicon SMF data: ArrayExpress E-MTAB-14461 (https://www.ebi.ac.uk/biostudies/arrayexpress/studies/E-MTAB-14461); PRO-seq data: ArrayExpress E-MTAB-14462 (https://www.ebi.ac.uk/biostudies/arrayexpress/studies/E-MTAB-14462); Custom scripts for data analyses: GitHub (https://github.com/Krebslabrep/Chatsirisupachai_Moene_2024). The source data of this paper are collected in the following database record: biostudies:S-SCDT-10_1038-S44320-025-00094-5.
