# Supplementary material for: Mouse promoters are characterised by low occupancy and high turnover of RNA polymerase II
Source: Mol Syst Biol. 2025 Mar 31;21(5):447–71. doi: 10.1038/s44320-025-00094-5 (PMC12048509; doi:10.1038/s44320-025-00094-5)
Supplement: Supplementary file 1 — Appendix [file 44320_2025_94_MOESM1_ESM.pdf]

Appendix

Mouse promoters are characterised by low occupancy and high turnover of RNA polymerase II

Kasit Chatsirisupachai[1,#], Christina J.I. Moene[1,2,#], Rozemarijn Kleinendorst[1], Elisa Kreibich[1,3], Nacho Molina[4*], Arnaud Krebs[1*]

1. Genome Biology Unit, EMBL Heidelberg, Meyerhofstraße 1, 69117 Heidelberg, Germany.
2. Present address: Division of Gene Regulation, Netherlands Cancer Institute, 1066 CX Amsterdam, The Netherlands and Oncode Institute, the Netherlands
3. Present address: ETH Zürich, Department for Biosystems Science and Engineering (D-BSSE), Basel, Switzerland.
4. Institut de Génétique et de Biologie Moléculaire et Cellulaire (IGBMC); Université de Strasbourg, 1 Rue Laurent Fries, 67404 Illkirch, France.

# These authors contributed equally.

*Correspondence: nacho.molina@igbmc.fr; arnaud.krebs@embl.de

Table of contents

Appendix Figure S1: Pausing index comparison between *Drosophila* and mouse promoters.....2
Appendix Figure S2: Association between CpG density and promoter accessibility..................3
Appendix Figure S3: Quality control of PRO-seq data...4

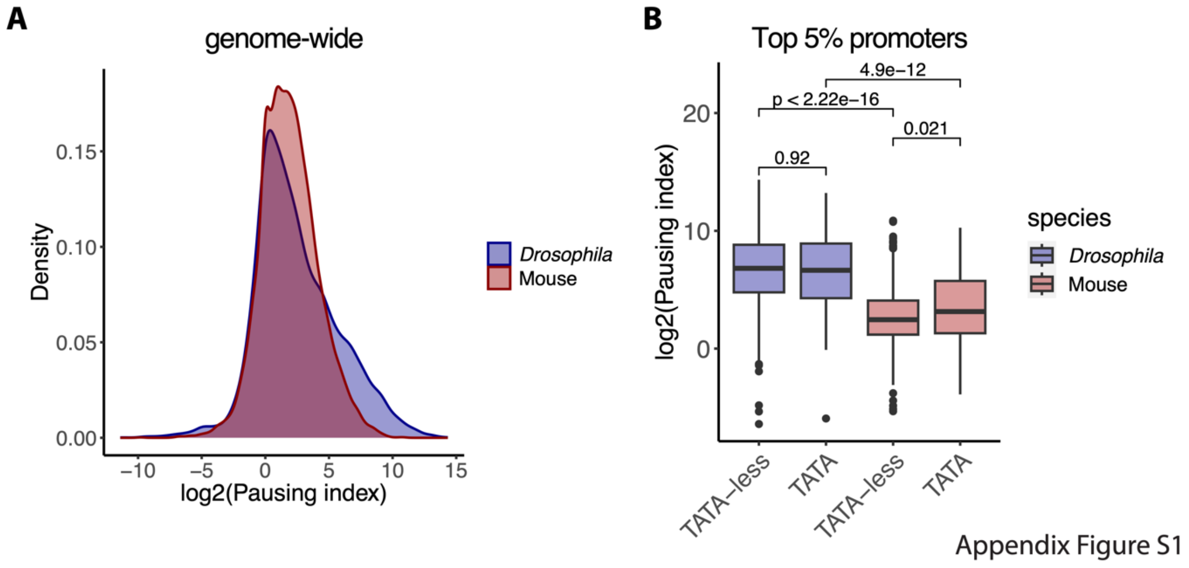

Appendix Fig. S1. Pausing index comparison between *Drosophila* and mouse promoters. (A) The genome-wide distribution of pausing index differs between species. Pausing index is calculated as the ratio of TSS reads [-150: 150] to gene body reads [+300:600]. The density plot is shown with the x-axis represents log2(Pausing index) from 13,459 mouse promoters and 8,466 *Drosophila* promoters. **(B)** Comparison of pausing index from the top 5% highly active mouse (n = 1,126) and *Drosophila* (n = 786) promoters. Boxplots display the distribution of log2(Pausing index). The middle line of the box represents the median. The box displays interquartile range (IQR), 25th to 75th percentile. Whiskers represent a distance of 1.5 × IQR. The analysis is stratified by the presence of a TATA-box at the promoter.

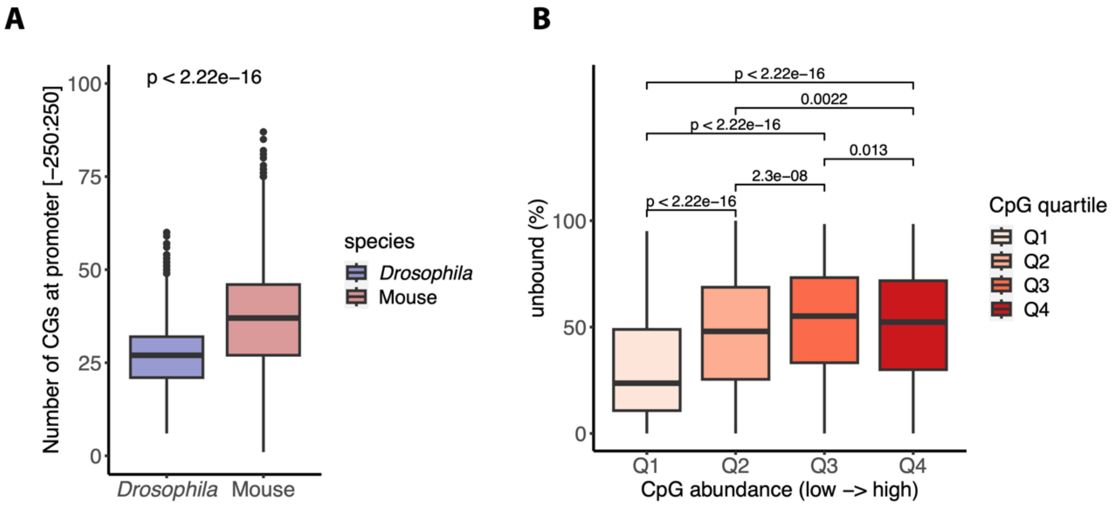

Appendix Figure S2

Appendix Fig. S2. Association between CpG density and promoter accessibility. (A) Comparison of promoter CGs between *Drosophila* and mouse. Number of CGs in the promoter region ([-250:250] bp around the TSS) was counted for each promoter used in the SMF analysis (Drosophila – n = 5,912 promoters; mouse – n = 6,122 promoters). Boxplots represent the distribution of the frequency of the number of CGs at each promoter. The middle line of the box represents the median. The box displays interquartile range (IQR), 25th to 75th percentile. Whiskers represent a distance of 1.5 × IQR. The statistical comparisons between groups were performed using Wilcoxon rank-sum test. **(B)** Mouse promoter accessibility as a function of CpG abundance. Mouse promoters were stratified into four quartiles by number of CGs located in the promoter region (Q1 – n = 1,579; Q2 – n = 1,565; Q3 – n = 1,466; Q4 – n = 1,512). Boxplots represent the distribution of the unbound state for promoters in different quartiles. The middle line of the box represents the median. The box displays interquartile range (IQR), 25th to 75th percentile. Whiskers represent a distance of 1.5 × IQR. Pairwise comparisons were performed using Wilcoxon rank-sum test. Multiple-hypothesis testing correction was done using Benjamini–Hochberg procedure.

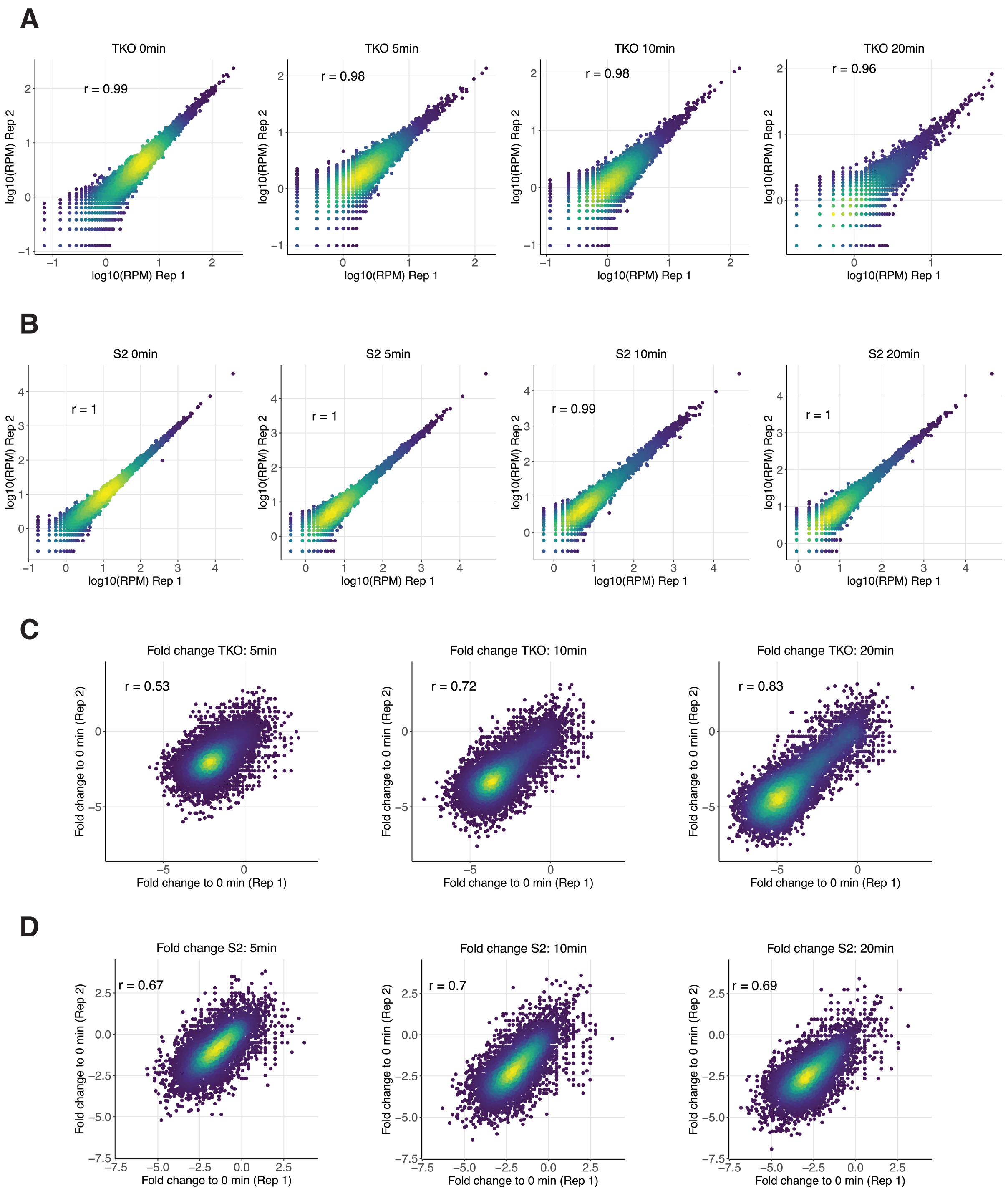

Appendix Figure S3

Appendix Figure S3. Quality control of PRO-seq data. (A) Scatter plots show correlation of the PRO-seq signal (log10(RPM) at the TSS [-100:200]; n = 24,869 promoters) in mouse TKO mESCs from two replicates of each TRP treatment time point, except in the 2.5 min TRP treatment as only one replicate passed the quality control. Pearson correlation coefficients are displayed. **(B)** Scatter plots show correlation of the PRO-seq signal (log10(RPM) at the TSS [-100:200]; n = 16,198 promoters) in *Drosophila* S2 cells from two replicates of each TRP treatment time point, except in the 2.5 min TRP treatment as only one replicate passed the quality control. Pearson correlation coefficients are displayed. **(C)** Scatter plots show correlation of the log2 fold change in PRO-seq signal upon TRP treatment with respect to PRO-seq signal at 0 min in mouse TKO mESCs from two replicates, except in the 2.5 min TRP treatment. Pearson correlation coefficients are displayed. **(D)** Scatter plots show correlation of the log2 fold change in PRO-seq signal upon TRP treatment with respect to PRO-seq signal at 0 min in *Drosophila* S2 cells from two replicates, except in the 2.5 min TRP treatment. Pearson correlation coefficients are displayed.
