# Supplementary material for: Mouse promoters are characterised by low occupancy and high turnover of RNA polymerase II
Source: Mol Syst Biol. 2025 Mar 31;21(5):447–71. doi: 10.1038/s44320-025-00094-5 (PMC12048509; doi:10.1038/s44320-025-00094-5)
Supplement: Supplementary file 13 — Expanded View Figures [file 44320_2025_94_MOESM13_ESM.pdf]

Expanded View Figures

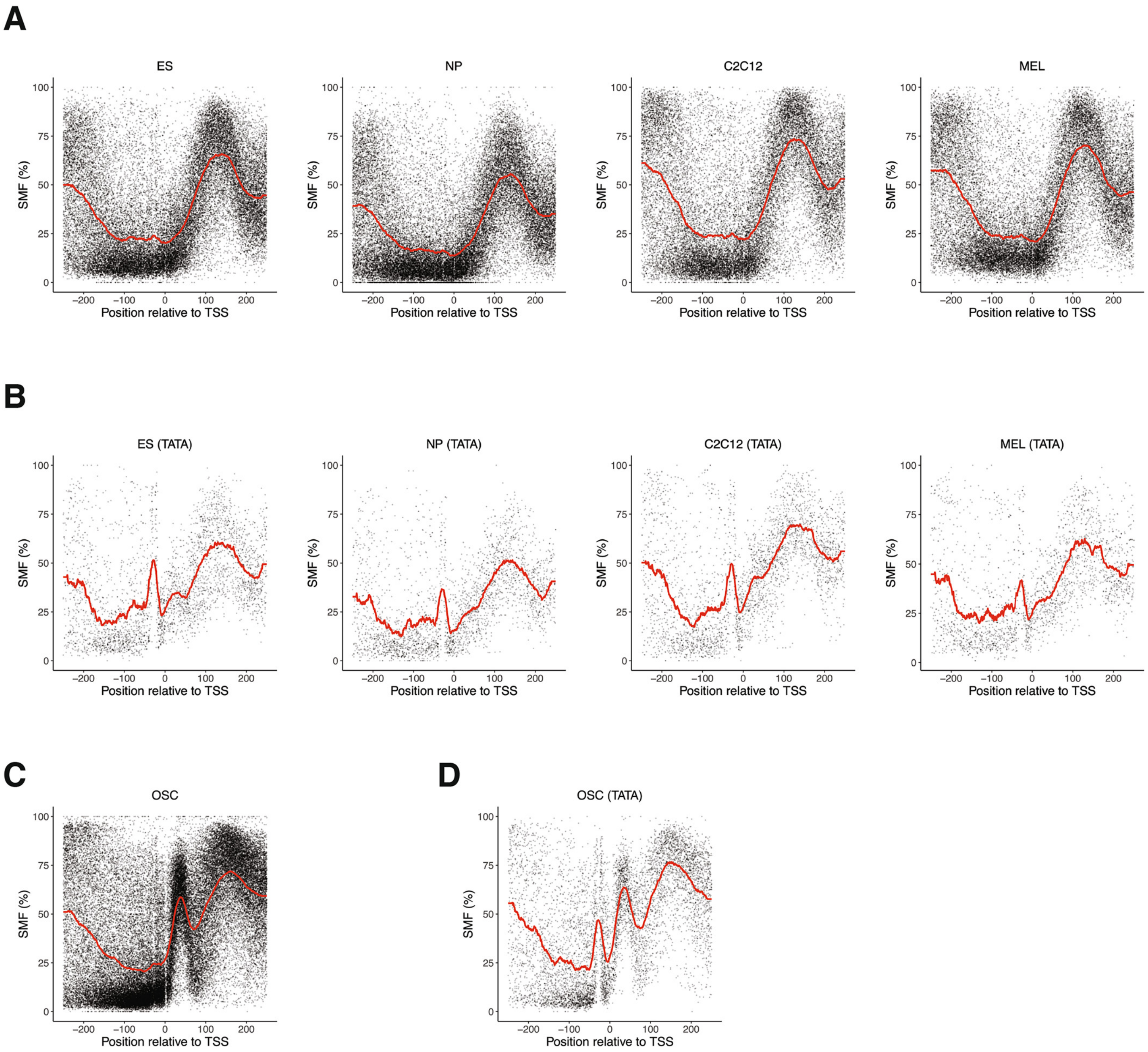

Figure EV1. Average promoter footprint across various cell lines.

Absence of Pol II footprints at highly active mouse promoters is not only restricted to TKO mESCs but also a property of other mouse cell types. Composite profile of SMF signal at (**A**) the TSSs of active promoters (top 5% Pol II ChIP-seq) and (**B**) the TSSs of active, TATA-box containing promoters (top 5% Pol II ChIP-seq with a TATA-box) in mouse wild-type embryonic stem cells (ES), neural progenitor cells (NP), myoblasts (C2C12), and erythrocytes (MEL). Prominent Pol II footprints at highly active *Drosophila* promoters in the ovarian somatic cell (OSC) line. Composite profile of SMF signal at (**C**) the TSSs of active promoters (top 5% Pol II ChIP-seq) and (**D**) the TSSs of active, TATA-box containing promoters (top 5% Pol II ChIP-seq with a TATA-box) in *Drosophila* OSCs. Shown is the footprinting frequency (1 - methylation [%]) of individual cytosines (black dots). The red line indicates the smoothed average signal over 20 bp.

 © The Author(s)

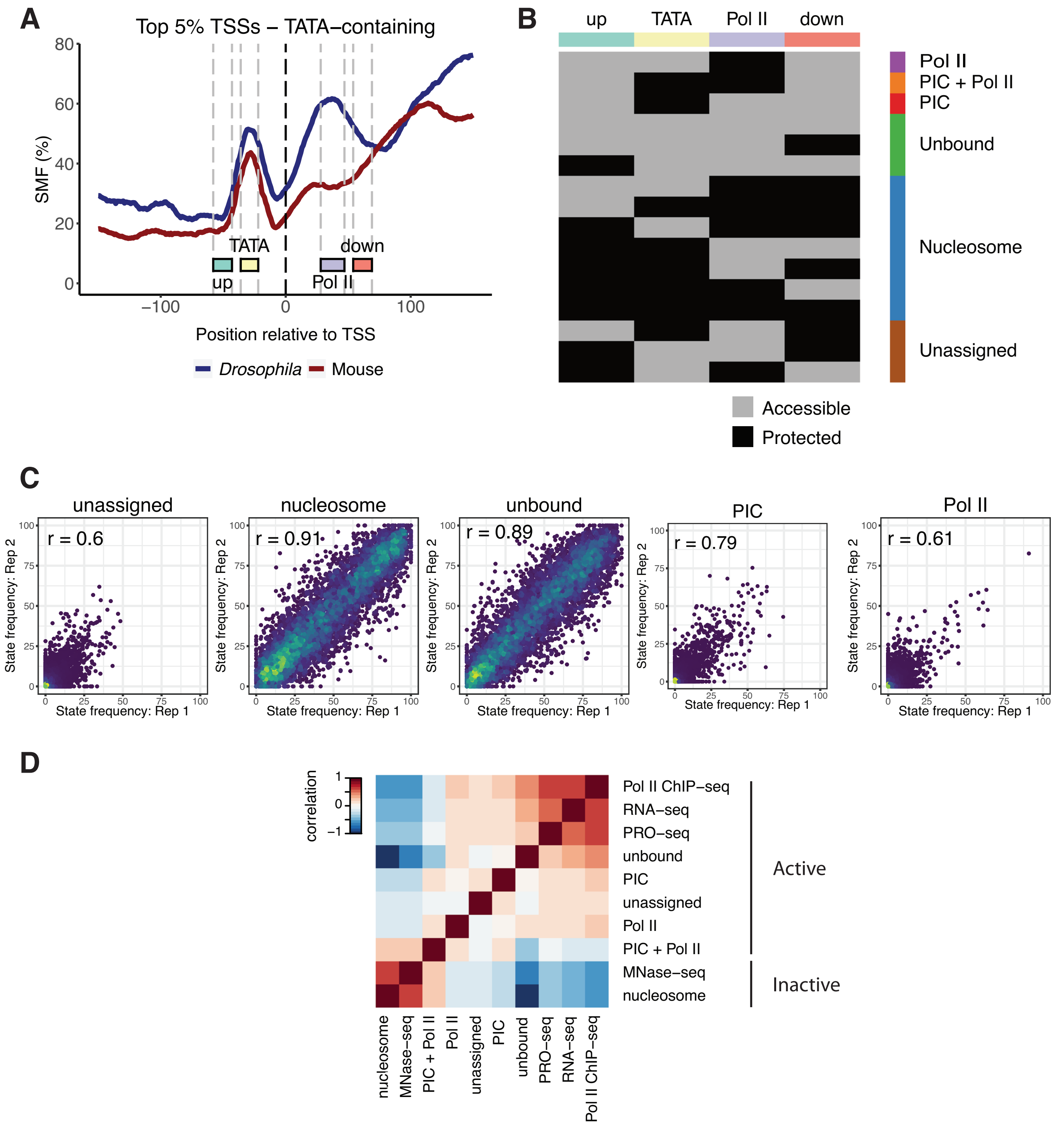
A
Top 5% TSSs – TATA–containing
SMF (%)
TATA
down
up
Pol II
Position relative to TSS
Drosophila
Mouse
B
up
TATA
Pol II
down
Pol II
PIC + Pol II
PIC
Unbound
Nucleosome
Unassigned
Accessible
Protected
C
unassigned
r = 0.6
nucleosome
r = 0.91
unbound
r = 0.89
PIC
r = 0.79
Pol II
r = 0.61
State frequency: Rep 2
State frequency: Rep 1
D
correlation
Pol II ChIP–seq
RNA–seq
PRO–seq
unbound
PIC
unassigned
Pol II
PIC + Pol II
MNase–seq
nucleosome
Active
Inactive

Figure EV2. Single-molecule promoter state decomposition in mouse promoters.

(**A**) A four-bin strategy to sort single DNA molecule into distinct promoter states. This bin strategy is adapted from Krebs et al, 2017 to include the downstream bin to better distinguish the Pol II footprint from the nucleosome footprint. Methylation status is binarized within each bin, creating a 4-bit vector that leads to 16 possible combinations, each describing the state of each molecule (see Methods for details). (**B**) Schematic representation of the methylation patterns used to define each promoter state (methylated, accessible Cs—light grey; unmethylated, protected Cs—black). The top horizontal annotation bar represents the four bins (upstream, TATA, Pol II, downstream) used for promoter state decomposition. The vertical side bar displays the promoter state corresponding to the occupancy type according to the bins. (**C**) Scatter plots show the correlation between state frequencies determined from each bait-capture SMF replicate ($n = 6122$ promoters). The states PIC + Pol II and Pol II are combined into a single Pol II state. Pearson correlation coefficients are displayed. (**D**) The global relationship between promoter state frequencies and independent bulk measurements of Pol II and nucleosomes. The heatmap shows the Spearman correlation between each dataset, ordered by hierarchical clustering. The states segregate into two groups correlating either with transcription/transcription machinery (RNA-seq, PRO-seq, and Pol II ChIP-seq) or nucleosomes (MNase-seq).

 © The Author(s)

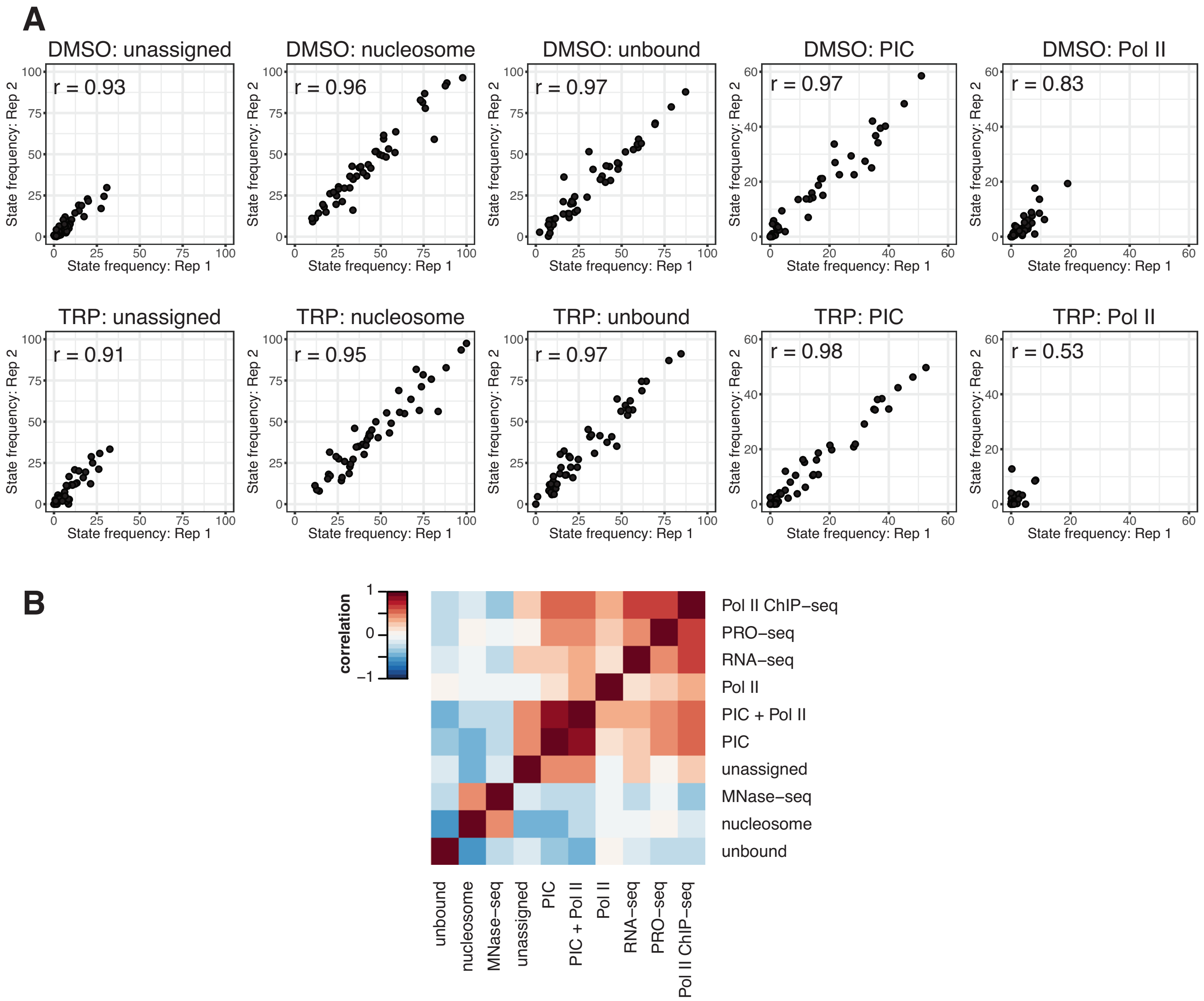

Figure EV3. Quality control of targeted amplicon SMF sequencing.

(**A**) Scatter plots illustrating the correlation between state frequencies determined from each amplicon SMF replicate. The states PIC + Pol II and Pol II are combined into a single Pol II state. Pearson correlation coefficients are displayed. (**B**) The global relationship between promoter state frequencies at selected promoters that were included in amplicon SMF ($n = 47$ promoters) and independent bulk measurements of Pol II and nucleosomes. The heatmap shows Spearman correlation between each dataset and is ordered by hierarchical clustering. States separate into two groups which either correlate with transcription/transcription machinery (RNA-seq, PRO-seq, and Pol II ChIP-seq) or nucleosomes (MNase-seq).

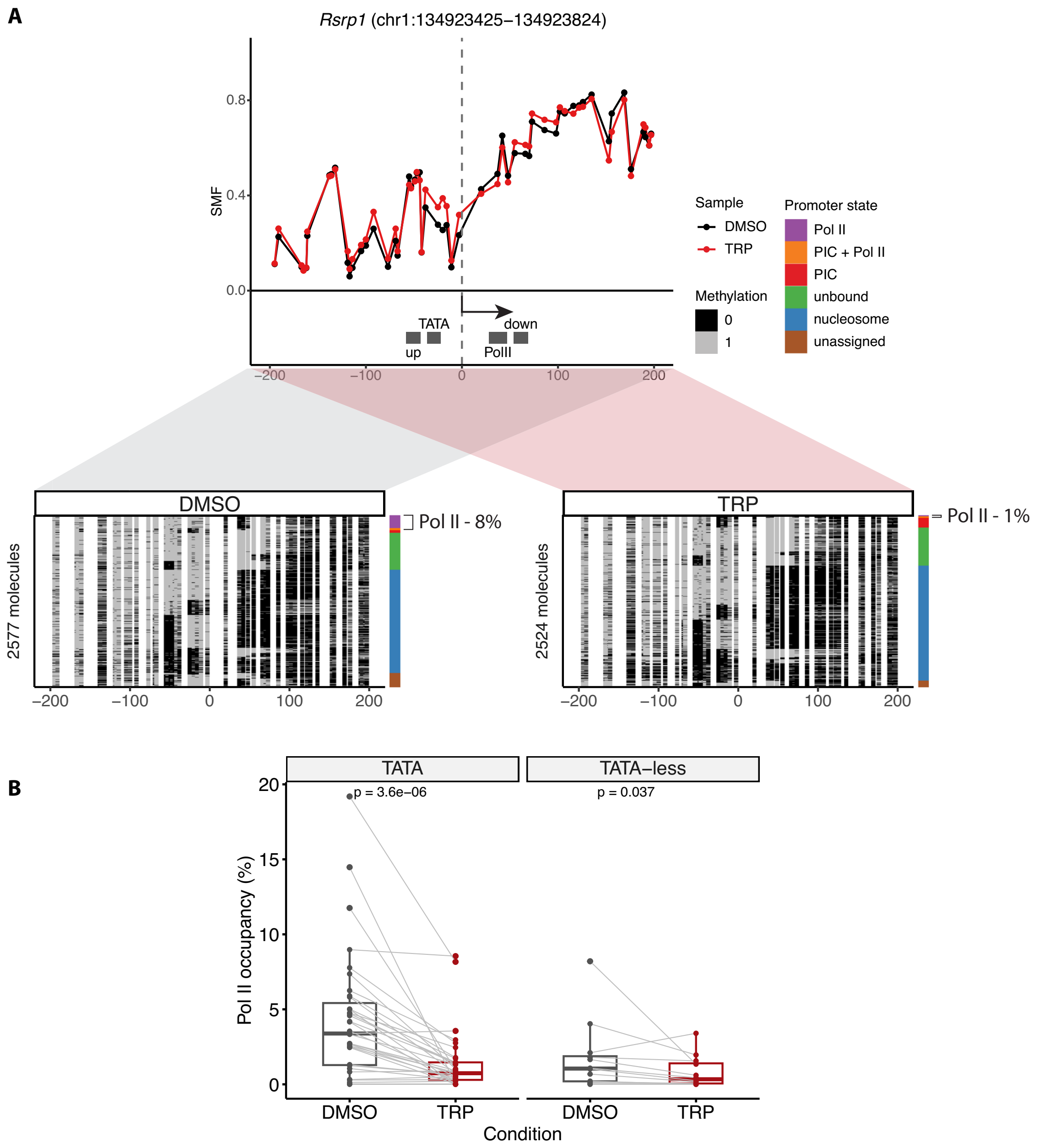

Figure EV4. Reduction in Pol II footprint upon transcription initiation inhibition at TATA-less mouse promoters.

(**A**) Single-site example of a reduction in Pol II footprint upon TRP treatment at the *Rsrp1* promoter. The upper panel shows the average SMF plot (DMSO—black, TRP—red). The positions of the four bins (upstream, TATA, Pol II, downstream) used for promoter state decomposition are shown (see Methods for details). The *x* axis represents the position relative to the TSS, while the y-axis shows the SMF signal (1 - methylation). The lower panel displays single-molecule stack plots for DMSO and TRP conditions. Each row denotes a single DNA molecule and the methylation status of each cytosine in that molecule (methylated, accessible—light grey; unmethylated, protected—black). The vertical sidebars display the frequency of each promoter state. The percentages of molecules harbouring footprints for the engaged Pol II are indicated on the right side of the plot. (**B**) Loss of Pol II occupancy upon inhibition of transcription initiation occurs in both TATA ($n = 35$) and TATA-less ($n = 12$) mouse promoters. Boxplots represent the distribution of the frequency of Pol II-bound molecules (Pol II and PIC + Pol II states). The middle line of the box represents the median. The box displays interquartile range (IQR), 25th to 75th percentile. Whiskers represent a distance of 1.5 × IQR. Statistical comparisons between groups were performed using the Wilcoxon signed-rank test.

 © The Author(s)

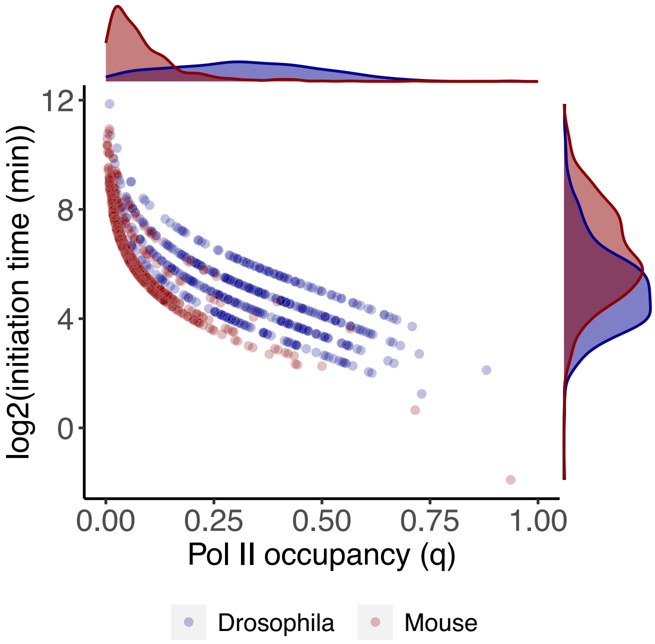

Figure EV5. Distribution of Pol II occupancy (q) and initiation time at highly active *Drosophila* and mouse promoters.

The x-axis displays Pol II occupancy (q), while y-axis represents log2(initiation time (min)). Each dot represents a promoter. Dot colours correspond to species (blue—*Drosophila*, red—mouse). The density plots on the top and on the right show the distribution of Pol II occupancy (q) and initiation time, respectively.
