# Supplementary material for: Mouse promoters are characterised by low occupancy and high turnover of RNA polymerase II
Source: Mol Syst Biol. 2025 Mar 31;21(5):447–71. doi: 10.1038/s44320-025-00094-5 (PMC12048509; doi:10.1038/s44320-025-00094-5)
Supplement: Supplementary file 4 — Peer Review File [file 44320_2025_94_MOESM4_ESM.pdf]

molecular systems biology

Mouse promoters are characterised by low occupancy and high turnover of RNA polymerase II

Kasit Chatsirisupachai, Christina Moene, Rozemarijn Kleinendorst, Elisa Kreibich, Nacho Molina, and Arnaud Krebs

Corresponding author(s): Arnaud Krebs (arnaud.krebs@embl.de) , Nacho Molina (nacho.molina@igbmc.fr)

Review Timeline:

Submission Date:	18th Sep 24
Editorial Decision:	5th Nov 24
Revision Received:	20th Jan 25
Editorial Decision:	14th Feb 25
Revision Received:	28th Feb 25
Accepted:	13th Mar 25

Editor: Poonam Bheda

Transaction Report:

(Note: With the exception of the correction of typographical or spelling errors that could be a source of ambiguity, letters and reports are not edited. Depending on transfer agreements, referee reports obtained elsewhere may or may not be included in this compilation. Referee reports are anonymous unless the Referee chooses to sign their reports.)

5th Nov 2024

Manuscript Number: MSB-2024-12625
Title: Mammalian promoters are characterised by low occupancy and high turnover of RNA polymerase II

Dear Dr. Krebs,

Thank you for the submission of your manuscript to Molecular Systems Biology. We have now received feedback from the three reviewers who agreed to evaluate your manuscript. As you will see from the reports below, the referees acknowledge the interest of the study and are overall supporting publication of your work pending appropriate revisions.

I think that the recommendations of the reviewers are rather clear and I therefore do not see the need to repeat the comments listed below. All issues raised would need to be satisfactorily addressed. Please let me know in case you would like to discuss in further detail, I would be happy to schedule a meeting or a call.

When submitting your revised manuscript, please carefully review the instructions that follow below. We perform an initial quality control of all revised manuscripts before re-review; failure to include requested items will delay the evaluation of your revision.

We require:

1) A .docx formatted version of the manuscript text (including legends for main figures, EV figures and tables). Please make sure that the changes are highlighted to be clearly visible. Alternatively you may choose to submit your manuscript as a LaTeX file.

2) Individual production quality figure files as .eps, .tif, .jpg (one file per figure). For guidance, download the 'Figure Guide PDF' (https://www.embopress.org/page/journal/17574684/authorguide#figureformat).

3) At EMBO Press we ask authors to provide source data for the main figures. Our source data coordinator will contact you to discuss which figure panels we would need source data for and will also provide you with helpful tips on how to upload and organize the files.

4) A .docx formatted letter INCLUDING the reviewers' reports and your detailed point-by-point responses to their comments. As part of the EMBO Press transparent editorial process, the point-by-point response is part of the Peer Review File (PRF), which will be published alongside your paper.

5) A complete author checklist, which you can download from our author guidelines (https://www.embopress.org/page/journal/17574684/authorguide#submissionofrevisions). Please insert information in the checklist that is also reflected in the manuscript. The completed author checklist will also be part of the PRF.

6) Please note that all corresponding authors are required to supply an ORCID ID for their name upon submission of a revised manuscript.

7) It is mandatory to include a 'Data Availability' section after the Materials and Methods. Before submitting your revision, primary datasets produced in this study need to be deposited in an appropriate public database, and the accession numbers and database listed under 'Data Availability'. Please remember to provide a reviewer password if the datasets are not yet public (see https://www.embopress.org/page/journal/17574684/authorguide#dataavailability).

In case you have no data that requires deposition in a public database, please state so in this section. Note that the Data Availability Section is restricted to new primary data that are part of this study.
This study includes no data deposited in external repositories.

8) All Materials and Methods need to be described in the main text using our 'Structured Methods' format, which is required for all research articles. According to this format, the Methods section includes a Reagents and Tools Table (listing key reagents, experimental models, software and relevant equipment and including their sources and relevant identifiers) followed by a Methods and Protocols section describing the methods using a step-by-step protocol format. The aim is to facilitate adoption of the methodologies across labs. Please upload the Reagents and Tools table as a separate document when submitting your revised manuscript. More information on how to adhere to this format as well as a downloadable template (.docx) for the Reagents and Tools Table can be found in our author guidelines:
https://www.embopress.org/page/journal/17444292/authorguide#structuredmethods

An example of a Method paper with Structured Methods can be found here:
https://www.embopress.org/doi/10.15252/msb.20178071.

9) For data quantification: please specify the name of the statistical test used to generate error bars and P values, the number (n) of independent experiments (specify technical or biological replicates) underlying each data point and the test used to calculate p-values in each figure legend. The figure legends should contain a basic description of n, P and the test applied. Graphs must include a description of the bars and the error bars (s.d., s.e.m.). Please provide exact p values.

10) Our journal encourages inclusion of *data citations in the reference list* to directly cite datasets that were re-used and obtained from public databases. Data citations in the article text are distinct from normal bibliographical citations and should directly link to the database records from which the data can be accessed. In the main text, data citations are formatted as follows: "Data ref: Smith et al, 2001" or "Data ref: NCBI Sequence Read Archive PRJNA342805, 2017". In the Reference list, data citations must be labeled with "[DATASET]". A data reference must provide the database name, accession number/identifiers and a resolvable link to the landing page from which the data can be accessed at the end of the reference. Further instructions are available at .

11) We replaced Supplementary Information with Expanded View (EV) Figures and Tables that are collapsible/expandable online. A maximum of 5 EV Figures can be typeset. EV Figures should be cited as 'Figure EV1, Figure EV2" etc... in the text and their respective legends should be included in the main text after the legends of regular figures.
- For the figures that you do NOT wish to display as Expanded View figures, they should be bundled together with their legends in a single PDF file called *Appendix*, which should start with a short Table of Content. Appendix figures should be referred to in the main text as: "Appendix Figure S1, Appendix Figure S2" etc.
- Additional Tables/Datasets should be labeled and referred to as Table EV1, Dataset EV1, etc. Legends have to be provided in a separate tab in case of .xls files. Alternatively, the legend can be supplied as a separate text file (README) and zipped together with the Table/Dataset file.
See detailed instructions here:
https://www.embopress.org/page/journal/17574684/authorguide#expandedview

12) For more information: There is space at the end of each article to list relevant web links for further consultation by our readers. Could you identify some relevant ones and provide such information as well? Some examples are patient associations, relevant databases, OMIM/proteins/genes links, author's websites, etc...

13) Author contributions: CRediT has replaced the traditional author contributions section because it offers a systematic machine readable author contributions format that allows for more effective research assessment. Please remove the Authors Contributions from the manuscript and use the free text boxes beneath each contributing author's name in our system to add specific details on the author's contribution. More information is available in our guide to authors.

14) Disclosure statement and competing interests: We updated our journal's competing interests policy in January 2022 and request authors to consider both actual and perceived competing interests. Please review the policy https://www.embopress.org/competing-interests and update your competing interests if necessary.

15) Every published paper now includes a 'Synopsis' to further enhance discoverability. Synopses are displayed on the journal webpage and are freely accessible to all readers. They include a short stand first (maximum of 300 characters, including space) as well as 2-5 one-sentences bullet points that summarizes the paper. Please write the bullet points to summarize the key NEW findings. They should be designed to be complementary to the abstract - i.e. not repeat the same text. We encourage inclusion of key acronyms and quantitative information (maximum of 30 words / bullet point). Please use the passive voice. Please attach these in a separate file or send them by email, we will incorporate them accordingly.

Please also suggest a striking image or visual abstract to illustrate your article as a PNG file 550 px wide x 300-600 px high.
Share synopsis text and image, as well as eTOC:
Please note that these would be the final versions and changes during proofing are usually not allowed

16) As part of the EMBO Publications transparent editorial process initiative (see our policy here: https://www.embopress.org/transparent-process#Review_Process), Molecular Systems Biology will publish online a Peer Review File (PRF) to accompany accepted manuscripts.
In the event of acceptance, this file will be published in conjunction with your paper and will include the anonymous referee reports, your point-by-point response and all pertinent correspondence relating to the manuscript. Let us know whether you agree with the publication of the PRF and as here, if you want to remove or not any figures from it prior to publication.
Please note that the Authors checklist will be published at the end of the PRF.

Molecular Systems Biology has a "scooping protection" policy, whereby similar findings that are published by others during review or revision are not a criterion for rejection. Should you decide to submit a revised version, I do ask that you get in touch after three months if you have not completed it, to update us on the status.

I look forward to receiving your revised manuscript.

Yours sincerely,

Poonam Bheda, PhD
Scientific Editor
Molecular Systems Biology

Reviewer #1:

The study describes the promoter occupancy landscape in two model species, namely Mus musculus and Drosophila melanogaster. These species have been shown to have different transcriptional burst patterns. However, the molecular basis of these features is unknown. The work utilises single-molecule footprinting (SMF) at promoter regions as a proxy for the occupancy of transcriptional machinery in Drosophila S2 cells and mouse embryonic stem cells; both initial datasets were described and analysed in previous studies from the same group [1,2]. Their SMF data suggest that promoter-proximal RNA polymerase II (Pol II) pausing at mouse promoters is less frequent than at Drosophila promoters despite presenting equivalent nascent transcription (measured by PRO-seq) and chromatin accessibility levels (measured by MNase-seq). To identify the underlying basis for these differences, the authors built a mathematical model that can explain Pol II occupancy at promoters solely as the ratio of Pol II turnover and initiation rates. The authors also found that the Pol II turnover rate at highly active genes seems to be faster in mouse promoters than in Drosophila promoters based on transcription initiation inhibition experiments. However, the observed difference in turnover rate is insufficient to explain the interspecies difference, indicating that these cell lines also may have different transcription initiation rates.

The key conclusion of the work is that mouse embryonic cells have less promoter-proximal Pol II pausing than Drosophila S2 cells due to having a lower initiation rate and a higher turnover rate. However, the author failed to disentangle the two elements contributing to the promoter-proximal Pol II turnover rate: (i) early termination and (ii) Pol II productive elongation.

Overall, the data are clear and support the conclusions without major issues. Drosophila and mouse cells are commonly used as models in gene expression studies, and our mechanistic understanding of transcription is strongly based on them. Thus, this work may be of general interest to the transcription community. Their findings support previous observations and hypotheses in the field. It also serves as a comparative analysis of Pol II dynamics in both organisms, which can lead to interesting findings in the future. The study exposes certain interspecies differences in how transcription may work, although fails to provide any mechanistic information. In addition, I'd suggest the following points to be taken into consideration:

Major points:

1. The SMF is based on the use of M.CviPI methyltransferase. Is it possible that some of the observed results are caused by DNA sequence bias of the enzyme or other aspects, such as the genome size difference? I'd recommend evaluating the SMF results in dechromatinized DNA from both cell lines.

Minor points

2. The observed differences are presented as interspecies features. However, some might be independent of their organism of origin, and due to intrinsic features of the cell lines (mESC vs S2). The author should consider these differences when discussing the observed results.

3. In Figure 1, mouse and Drosophila promoters are compared based on ranked Pol II ChIP-seq signal as equivalent (Top 5% TSS). While this might be the case, other features can be confounding. What is the Pol II ChIP-seq signal and Pausing index in both groups of genes? It could also be informative to compare the promoter of genes with the same transcriptional output (using PRO-seq or Pol II ChIP-seq reads over gene bodies, for example). This might help to explain the differences in Pol II kinetics.

4. The large proportion of open promoters in mouse cells is surprising (Figure 2C). Around a third of mouse promoters have more than 50% of molecules free of nucleosomes. This observation agrees with a lower transcription initiation rate in murine cells and may suggest differences in PIC formation between both cell lines. Could the author speculate about these results?

5. In the same line, the promoter state frequencies in 2B and 2D don't match those in 2A and 2C. For example, in Figure 2B, around 37% of Svil gene molecules are categorised as PIC (in red). However, in Figure 2A, the PIC category (in red) looks lower than 10% in all shown genes. Could the authors please explain these discrepancies?

6. Determining which component of the Pol II turnover rate changes the most in mouse cells as compared to Drosophila cells could significantly impact the conclusions. Therefore, I suggest assessing the mouse promoter occupancy upon Integrator or p-TEFb perturbations.

1. Krebs AR, Imanci D, Hoerner L, Gaidatzis D, Burger L, Schübeler D. Genome-wide Single-Molecule Footprinting Reveals High RNA Polymerase II Turnover at Paused Promoters. Mol Cell. 2017 Aug 3;67(3):411-422.e4.
doi: 10.1016/j.molcel.2017.06.027.
2. Sönmezer C, Kleinendorst R, Imanci D, Barzaghi G, Villacorta L, Schübeler D, Benes V, Molina N, Krebs AR. Molecular Co-occupancy Identifies Transcription Factor Binding Cooperativity In Vivo. Mol Cell. 2021 Jan 21;81(2):255-267.e6.
doi: 10.1016/j.molcel.2020.11.015.
3. Tullius TW, Isaac RS, Dubocanin D, Ranchalis J, Churchman LS, Stergachis AB. RNA polymerases reshape chromatin architecture and couple transcription on individual fibers. Mol Cell. 2024 Sep 5;84(17):3209-3222.e5. doi: 10.1016/j.molcel.2024.08.013.

Reviewer #2:

Summary
Single Molecule Footprinting has revolutionized our understanding of transcriptional processes, as we now have the ability to detect protein-DNA interactions at single-molecule resolution, without experimental enrichment of sequences of interest, or assaying binding in bulk. In the paper titled, "Mammalian promoters are characterized by low occupancy and high turnover of RNA polymerase II", the authors use SMF to directly quantify Pol II-DNA contacts to identify exact binding frequencies rather than relative occupancy. This study applies SMF to measure Pol II occupancy in both fly and mouse cells, to answer a long-standing question and paradox in the field: Why do major differences exist in metazoan transcription kinetics, although a conserved set of transcription regulators are used with similar binding profiles? The timescales on which transcription occurs is drastically different; on fly promoters, the active and inactive state occur at similar frequencies, on the order of minutes. In contrast, on mouse promoters, very long inactive states (minutes to hours) are interspersed by short active states. By using SMF in Drosophila S2 cells and mouse ESCs, the authors identify that mouse promoters have reduced Pol II occupancy compared to fly promoters, which was previously not appreciated using bulk cell assays. By combining experimental data with mathematical modeling, they also show that this reduced Pol II occupancy in the mouse is consistent with 1) reduced initiation rates, and 2) higher Pol II turnover.

Strengths of this study include integrating experimental results with modeling, comparison of single-molecule approaches to extant bulk sequencing data, and establishing that the results are not a mouse ESC-specific feature, but reproducible across various mouse cell lines. Limitations include the oversimplification that "mammalian" promoters have the features described above, although no human cell lines were assayed. Overall, this study makes significant contributions to our understanding of transcriptional regulation, particularly in highlighting species-specific differences and challenging the existing model of preloaded polymerases waiting for release. Instead, the findings support a model of rapid cycles of initiation and early termination for RNA Pol II on mouse promoters, which remains to be experimentally validated. Overall, this is a well-written manuscript with compelling data, beautiful figures, and interesting possible experimental follow-ups. There are a few concerns for the authors to address, that would mostly require editing in the text and in the figures.

Major points
The authors convincingly show that mammalian promoters generally have less Pol II occupancy and faster turnover compared to Drosophila. However, it would be valuable to explore the distribution of promoter behaviors more thoroughly across both species. Specifically: Is there a continuum of promoter types in both species, characterized by varying initiation rates and turnover speeds? If so, do the distributions simply differ between species, with mammalian promoters skewed towards lower occupancy and faster turnover? Alternatively, are there distinct categories of promoter behavior that are unique to each species? Do any mammalian promoters exhibit "Drosophila-like" behavior (high occupancy, slower turnover) or vice versa? This analysis would help clarify whether the differences between species represent a fundamental change in promoter regulation or a more subtle shift in the prevalence of certain promoter types.

The conclusion that "This suggests that a low amplitude Pol II footprint is a general feature of mammalian promoters" is a bit too strong (page 5), when 5 different mouse cell lines were used, but no human cell lines were used to determine the generalizability of the findings across mammals. In general, this conclusion will have to be softened throughout the text. In the discussion the authors mention that their data are in agreement with previous studies conducted in human cells. It could be nice to elaborate on this a bit more.

Minor points:
When introducing their SMF approach or in the results sections, it would be valuable to discuss a bit more the other SMF

methods and publications that have emerged since the pioneering Krebs et al. 2017 paper. A brief discussion of how these other approaches compare to the one used here, and whether their findings are consistent with the original Krebs data, would help substantiate the findings described here.

Italicize gene names, such as Svil, Skp1, and Rsrp1, when mentioning them in-text, and in the figures. Other gene names are italicized, but please check that this is consistent throughout the manuscript.
"We found that the states were separated in two clusters..." (page 5) is referencing Figure EV2D, but it is not clear how that panel indicates the "active" and "inactive" promoter states. Does this mean the "transcription" cluster is the active, and the "nucleosome" cluster is the inactive? Perhaps labeling that somehow would make the conclusion and the figure make more sense together.

Figure 2:
Panel A and C show Pol II ChIP-seq rank, but it's not intuitive how it's currently labeled, as there are no values on the x-axis. In the text, the authors indicate that there is "increased promoter activity", presumably as one moves to the right on the x-axis, but perhaps that can be indicated in some way for both panels. In the figure legend, it may be useful if the authors indicate that each vertical line in the plot represents a different promoter.

It's unclear why for Svil or Skp1, the state frequencies as shown on the right in B and D don't correspond to the breakdown in the vertical line they're coming from in A and C. For instance, for Svil it looks like it's coming from a region where the "nucleosome" state frequency is ~50%, but for Svil specifically, it's <25%, by eye. Some clarification in the figure and legend would be helpful.

Please include a legend between panel B and D to indicate what the gray and black lines represent (as done for Figure 3A).

It would be helpful if the colors of panels E and F are changed to blue and red for Drosophila and Mouse, respectively, as was done for Figure 3B, for the sake of consistency. Please include p-values between TATA-less and TATA promoters for both panels as well, as they are indicated to be statistically significantly different in the text.

In the legend, for F, the authors indicate that "the number of promoters... is similar". Are the same exact promoters used, or just the promoter number is the same? Please indicate the n, as is done in (E).

Figure EV2:
Please include a legend for panel B, to indicate what gray and black mean. For "unassigned", it would be helpful if it was a different color throughout the entire manuscript, since the same gray color is used to signify "inaccessible" for the methylation status.

The authors state that "A reduction in the Pol II footprint was also evident at the TATA-less Rsrp1 promoter" and give specific %, referencing
Figure EV4. It's not clear from the graph in Figure EV4 where the reduction is observed with TRP, since it's so slight in the "Pol II" bin. Is this where the 8% to 1% reduction comes from? Are there any other TATA-less promoters with a greater reduction that can be used as an alternate example?

Figure 6 could use a small change, to make the model clearer: Please move the legend (red/blue arrows) on the left or right of the model, since its current placement suggests that the left side of the model indicates the "mouse" model and the right side the "Drosophila" model.

Reviewer #3:

MSB-2024-12625
Mammalian promoters are characterised by low occupancy and high turnover of RNA polymerase II
Chatsirisupachai, Moene, Kleinendorst, Kreibich, Molina, Krebs

General comments

I enjoyed reading this paper. It is clearly written; addresses an important and topical problem. It draws important biological insights through an effective integration of quantitative experiments and theory.
The approach is particularly appealing in showing how a clear and simple mathematical model can lead to informative biological conclusions.

As always in exploiting a model, the underlying assumptions are hugely significant. Some of the assumptions that have been made appear rather arbitrary o me and one concern that came to mind is whether the conclusions are sensitive to the choices that have been made. One set of choices have to do with the selection of the 4 DNA sequence bins that define the UP, TATA, POL II and DOWN states

(Fig.EV2A). It does seem unlikely that small changes here would make a significant difference but it still would be helpful to have more justification for the choices made This is important also because these choices influence the interpretation of the resulting 16 regulatory patterns shown in Fig.EV2B. The assumptions made here are more puzzling. In particular, if we use binary notation to signify the regulatory patterns, then 0010 is interpreted as Pol II being present but 1010 is deemed to be unassigned, although it includes 0010 as a sub-pattern. I would have expected monotonicity of patterns, so that what is present in a sub-pattern does not disappear in a pattern that contains it, at least, not without a clear justification. For example, 0011 also contains the sub-pattern 0010 but, in this case, perhaps it could be argued that the 11 is more likely to signify a nucleosome. However, if that is the case, it might only be justified by a closer look at the underlying methylation pattern, rather than the very coarse 0/1 quantisation of the fraction of methylated sites that is used to define the state of each bin. I do not think the choices made in Fig.EV2B have been adequately justified. Since these choices are fundamental to the model that follows, I would encourage the authors to clarify their reasoning.

Aside from this point, the comments I have are minor. I believe this paper makes an important contribution to our understanding of gene regulation and it is pleasure to recommend it for publication.

Jeremy Gunawardena

Specific comments

1. Page 4, bottom para. I suggest annotating Figs.1E and 1F to show which peaks correspond to the PIC, Pol II pausing and +1 nucleosome. I found myself moving back and forth between the text and the figure to keep track of this and ended up scribbling a note on the figure.

2. Page 5, para 2. See the general comment above on the choices made in Fig.EV2.

3. Page 8, first line. The derivation of the sigmoid function could be simpler that what is given in the Methods (pages 17 & 18). The differential equations are not needed. From the model in Fig.4A, because there is no net flux through a pipeline, since it has nowhere to go at the ends, it must be that, in steady state, k_i . P_open = k_t . P_Pol II, so that P_open / P_Pol II = k_t / k_i = r. Hence, for the quantity being considered, P_Pol II / (P_open + P_Pol II) = 1 / (1 + r). This algebra is so simple that I would encourage the authors to put it in the main text, so that readers can gain an intuition for the approach.

4. page 8, para 3, line 1. "specie" --> species

5. page 21, Fig.4 caption. The Open pattern is defined as "the promoter is unbound by either nucleosome or Pol II" and later as "unbound and PIC states from SMF". The second is more accurate than the first and should be used exclusively. There are 3 plots in Fig.4B but the one on the right is not described. It would be clearer to say something like "the plot at the top shows the distribution of r on the data and the plot on the right shows the distribution of q on the data".

6. page 23, lines -3, -5. "log_2 fold change" with respect to what? Presumably the PRO-seq signal at time 0 but it would be better to say so.

Figure 4. The vertical arrow entering the green region annotated with k_i and the horizontal arrow leaving the purple region annotated with k_t are in the wrong place. The annotations should be placed instead on the pair of arrows on the boundary between the green and purple regions.

Figure EV5. The horizontal alignment of the "r = xxxx" in panel A is bad in several of the plots. Replace "Rep 1" and "Rep 2" in panels C and D with the corresponding units

Authors' response to the Reviewers' comments

Manuscript: MSB-2024-12625
Title: Mammalian promoters are characterised by low occupancy and high turnover of RNA polymerase II

Dear Dr Poonam Bheda,

Thank you for considering our manuscript for publication in *Molecular Systems Biology*. We were glad to read that all three reviewers were enthusiastic about our manuscript and commented about the importance of our finding for a broad audience. We carefully read the comments and suggestions from the reviewers, and we revised our manuscript accordingly. Based on the suggestions, we performed new analysis that led to 8 new panels in the revised version of the manuscript. We have also extensively edited the text and figures to clarify important issues raised by the reviewers. Please find below our point-by-point response to the comments from the reviewers. We hope that you will find the revised version of the manuscript appropriate for publication.

Best regards

Arnaud Krebs

Reviewer #1:

The study describes the promoter occupancy landscape in two model species, namely Mus musculus and Drosophila melanogaster. These species have been shown to have different transcriptional burst patterns. However, the molecular basis of these features is unknown. The work utilises single-molecule footprinting (SMF) at promoter regions as a proxy for the occupancy of transcriptional machinery in Drosophila S2 cells and mouse embryonic stem cells; both initial datasets were described and analysed in previous studies from the same group [1,2]. Their SMF data suggest that promoter-proximal RNA polymerase II (Pol II) pausing at mouse promoters is less frequent than at Drosophila promoters despite presenting equivalent nascent transcription (measured by PRO-seq) and chromatin accessibility levels (measured by MNase-seq). To identify the underlying basis for these differences, the authors built a mathematical model that can explain Pol II occupancy at promoters solely as the ratio of Pol II turnover and initiation rates. The authors also found that the Pol II turnover rate at highly active genes seems to be faster in mouse promoters than in Drosophila promoters based on transcription initiation inhibition experiments. However, the observed difference in turnover rate is insufficient to explain the interspecies difference, indicating that these cell lines also may have different transcription initiation rates.

The key conclusion of the work is that mouse embryonic cells have less promoter-proximal Pol II pausing than Drosophila S2 cells due to having a lower initiation rate and a higher turnover rate. However, the author failed to disentangle the two elements contributing to the promoter-proximal Pol II turnover rate: (i) early termination and (ii) Pol II productive elongation.

Overall, the data are clear and support the conclusions without major issues. Drosophila and mouse cells are commonly used as models in gene expression studies, and our mechanistic understanding of transcription is strongly based on them. Thus, this work may be of general interest to the transcription community. Their findings support previous observations and hypotheses in the field. It also serves as a comparative analysis of Pol II dynamics in both organisms, which can lead to interesting findings in the future. The study exposes certain interspecies differences in how transcription may work, although fails to provide any mechanistic information. In addition, I'd suggest the following points to be taken into consideration:

###We thank the reviewer for the constructive comments that help us substantially improve our manuscript. We were pleased to read that they think that the conclusions are of general interest and well supported by the data.

Major points:

1. The SMF is based on the use of M.CviPI methyltransferase. Is it possible that some of the observed results are caused by DNA sequence bias of the enzyme or other aspects, such as the genome size difference? I'd recommend evaluating the SMF results in dechromatinized DNA from both cell lines.

We agree that potential preference of the methyl-transferases is a potential source of bias in our SMF assay, and that this parameter should be carefully controlled for. We have previously evaluated the potential sequence preferences of M. CviPI and M.SssI enzymes. We performed a titration experiment on naked lambda DNA (Kleinendorst et al., 2021, Nature Protocols), and quantified methylation rates at different enzyme concentrations in a possible 4-mer contexts surrounding the methylated cytosine. We found that M. SssI has no preference for any 4-mer context, while M. CviPI shows a slight bias toward some nucleotide contexts (**Rebuttal Fig 1**, from DNA (Kleinendorst et al., 2021, Nature Protocols)). While this enzymatic bias is detected in the lower concentration range of the titration, that are no longer detectable upon saturation at higher enzyme concentrations. For instance, at 10 units/µg of DNA, the average methylation level is >95% methylation in all contexts.
To avoid confounding effects of this enzymatic preference, we perform SMF in saturation condition using large excess of enzyme (200 units/µg of DNA for M. CviPI and 60 units/µg of DNA for M. SssI). Moreover, to avoid potential differences between species with largely different genome sizes such as *Drosophila* and mouse, we adjusted the number of cells to use in our SMF protocol to keep constant amounts of substrate DNA in our reaction (0.25×10^6 mammalian cells or 2.5×10^6 *Drosophila* cells which corresponds to roughly 1 µg of DNA).

We now make explicit mention of the strategy to differences in methylation preference between species in the manuscript as follows:
"To mitigate potential bias in the assay resulting from difference in genome size between species, the number of cells used in the SMF protocol was adjusted to the substrate DNA constant in the reaction (Kleinendorst et al., 2021; Krebs et al., 2017; Sönmezer et al., 2021). Additionally, we performed the experiments under saturating enzyme conditions where sequence-specific preferences of the methyltransferase are no longer detectable (Kleinendorst et al., 2021)."

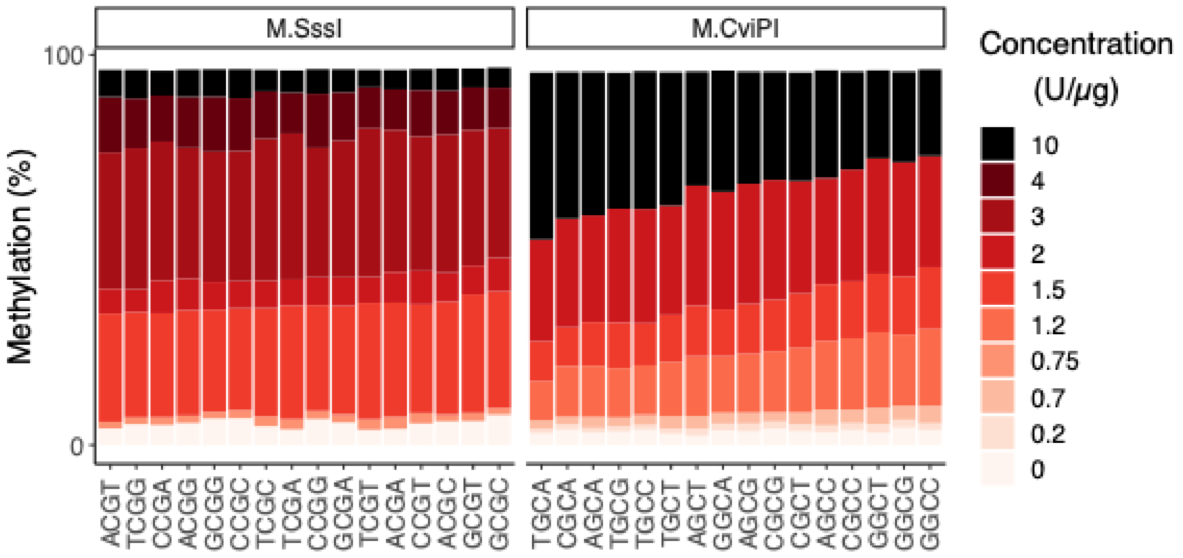

Rebuttal Fig. 1. In vitro methylation of naked lambda DNA demonstrates negligible sequence bias in methylation levels under saturated concentration (10 units/µg) of M. SssI and M. CviPI enzymes. The sequencing data is from (Kleinendorst et al., 2021).

Minor points

2. The observed differences are presented as interspecies features. However, some might be independent of their organism of origin, and due to intrinsic features of the cell lines (mESC vs S2). The author should consider these differences when discussing the observed results.

We agree with the reviewer that mESC and S2 cells are cell types of very different origin and that cell identity may provide an alternative explanation for the differences in Pol II occupancy observed. To tease apart between the interspecies or cell-type hypothesis, we compared the Pol II occupancy across multiple cell types in each species. We had initially compared Pol II footprints in mouse embryonic stem cells with those observed in mammalian somatic cell lines including neural progenitor, myoblasts, and erythrocytes (**Fig. EV1A,B**). We found that Pol II footprints are of comparable low frequency at highly active mouse promoters in these somatic cell lines. We now also compared Pol II footprints observed in S2 cells that are derived from the late-stage *Drosophila* embryo, with those of *Drosophila*

Ovarian somatic cells (OSC, **Fig. EV1C,D**). We found that *Drosophila* somatic cells also have high-amplitude Pol II footprints. Thus, we confirmed that both embryonic and somatic cell lines show remarkably similar Pol II occupancy levels in each species, suggesting that the observed differences are more likely to arise from interspecies differences rather than cell identity. We have incorporated the analysis of SMF data in OSC cells into the result section of the current version of the manuscript (**Fig. EV1C,D**) as follows:

"We next wondered if the observed differences may be a specific feature of mESCs that are in a pluripotent state. We compared the average profiles around the TSS of highly active genes in somatic cell lines representing various cell lineage (Kreibich et al., 2023). We observed very similar profiles in all the tested cell lines, with strong footprints for the PIC and low Pol II footprints downstream of the TSS (**Fig. EV1A,B**). In contrast, the prominent Pol II footprint is also found at the promoters of *Drosophila* ovarian somatic cells (OSC) (Krebs et al., 2017), in addition to the S2 cells (**Fig. EV1C,D**). These results suggest that a low amplitude Pol II footprint is a general feature of mouse promoters. Together, these observations suggest that the spatial patterns of promoter occupancy are globally conserved from *Drosophila* to mouse, but that Pol II occupancy levels may be reduced at mouse promoters."

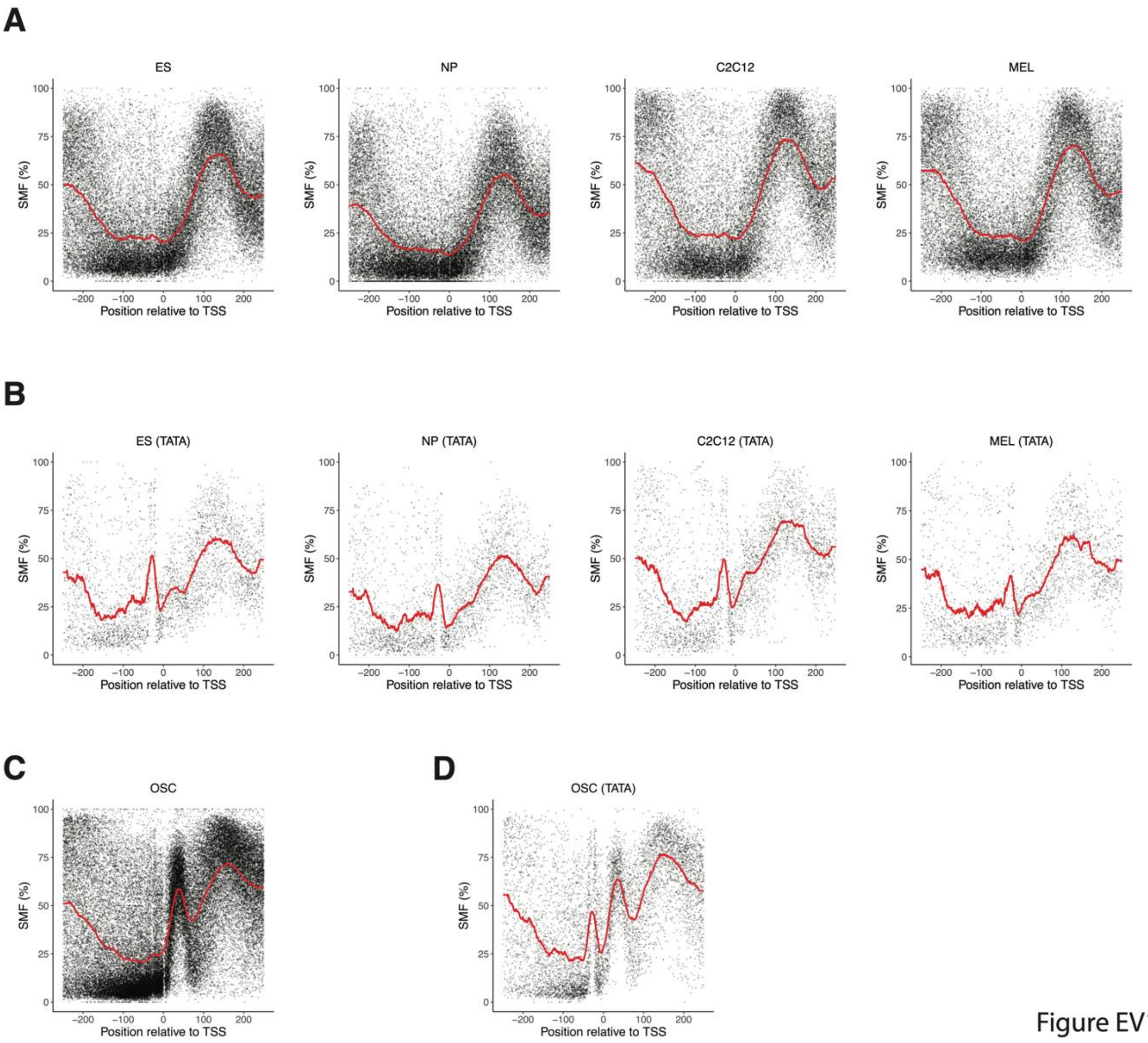

Figure EV1

Fig. EV1. Average promoter footprint across various cell lines. Absence of Pol II footprints at highly active mouse promoters is not only restricted to TKO mESCs but also a property of other mouse cell types. Composite profile of SMF signal at **(A)** the TSSs of active promoters (top 5% Pol II ChIP-seq) and **(B)** the TSSs of active, TATA-box containing promoters (top 5% Pol II ChIP-seq with a TATA-box) in mouse wild-type embryonic stem cells (ES), neural progenitor cells (NP), myoblasts (C2C12), and erythrocytes (MEL). Prominent Pol II footprints at highly active *Drosophila* promoters in the ovarian somatic cell (OSC) line. Composite profile of SMF signal at **(C)** the TSSs of active promoters (top 5% Pol II ChIP-seq) and **(D)** the TSSs of active, TATA-box containing promoters (top 5% Pol II ChIP-seq with a TATA-box) in *Drosophila* OSCs. Shown is the footprinting frequency (1 − methylation [%]) of individual cytosines (black dots). The red line indicates the smoothed average signal over 20 bp.

3. In Figure 1, mouse and Drosophila promoters are compared based on ranked Pol II ChIP-seq signal as equivalent (Top 5% TSS). While this might be the case, other features can be confounding. What is the Pol II ChIP-seq signal and Pausing index in both groups of genes? It could also be informative to compare the promoter of genes with the same transcriptional output (using PRO-seq or Pol II ChIP-seq reads over gene bodies, for example). This might help to explain the differences in Pol II kinetics.

We thank the reviewer for the suggestions to use orthogonal metrics to characterise the differences in promoter occupancy. Since mouse and *Drosophila* contain different genome size and gene length, it is challenging to perform a side-by-side comparison of gene body reads using bulk genomic assays like ChIP-seq or PRO-seq. However, we acknowledge that the pausing index – the ratio of Pol II levels at the promoter to those at the gene body – is a dimensionless metric and can be compared between species (Day et al., 2016). We found that, overall, mouse promoters (n = 13,459) have lower pausing index than *Drosophila* promoters (n = 8,466) (**Appendix Fig. S1A**). This trend can also be observed when we selected only top 5% promoters (**Appendix Fig. S1B**). However, analysing pausing index alone cannot tease apart whether the lower pausing index in mouse genes comes from decreased promoter-proximal Pol II, or increased accumulation of Pol II in the gene body. Thus, we focused our analysis on Pol II at the pausing site, where we can measure Pol II occupancy using SMF and found low Pol II occupancy at mouse promoters.
We have incorporated the pausing index analysis in the manuscript as follows:
"This low Pol II footprint at mouse promoters is however consistent with lower pausing index in mouse compared to *Drosophila* (defined as the ratio of active Pol II levels measured by PRO-seq at the promoter over the gene body) (**Appendix Fig. S1**)."

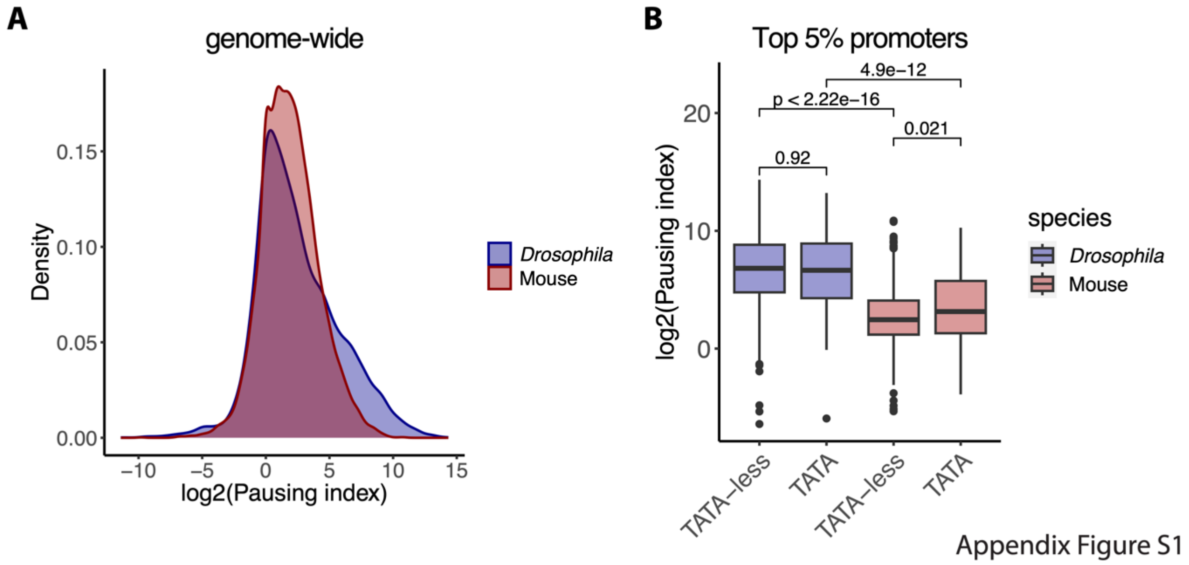

Appendix Fig. S1. Pausing index comparison between *Drosophila* and mouse promoters. (A) The genome-wide distribution of pausing index differs between species. Pausing index is calculated as the ratio of TSS reads [-150:150] to gene body reads [300:600]. The density plot is shown with the x-axis represents log2(Pausing index) calculated from 13,459 mouse promoters and 8,466 *Drosophila* promoters. **(B)** Comparison of pausing index from the top 5% highly active mouse (n = 1,126) and *Drosophila* (n = 786) promoters. Boxplots display the distribution of log2(Pausing index). The analysis is stratified by the presence of a TATA-box at the promoter.

4. The large proportion of open promoters in mouse cells is surprising (Figure 2C). Around a third of mouse promoters have more than 50% of molecules free of nucleosomes. This observation agrees with a lower transcription initiation rate in murine cells and may suggest differences in PIC formation between both cell lines. Could the author speculate about these results?

We agree with this observation that active mouse promoter have higher levels of accessibility. A possible explanation could come from the CpG-rich nature of mammalian promoters (Deaton and Bird, 2011; **Appendix Fig. S2A**) and the presence of CpG islands (CGIs) that are associated with nucleosome depletion (Fenouil et al., 2012). In support with this hypothesis, we observed a higher "unbound" state when classifying mouse promoters based on their CpG content (**Appendix Fig. S2B**), highlighting the association between CG density and promoter accessibility. Therefore, the high fraction of nucleosome-free molecules at mouse promoters could result from an inherent difference in species-specific promoter DNA sequence contexts. However, how the presence of CGIs mechanistically contributes to nucleosome depletion remains to be investigated. We, however, found a similar PIC occupancy between the two cell lines (**Fig. 2E**). This result thus suggest that PIC formation might be similar between the two species, regardless of the difference in promoter accessibility. Therefore, the difference in Pol II occupancy is more likely to stem from the processes downstream of PIC formation, namely transcription initiation and Pol II turnover.

We commented about these aspects in the new version of the manuscript:

"We additionally noted that upon activation mouse promoters are generally less occupied by nucleosome than *Drosophila* promoters (**Fig. 2A, C**). This difference correlates with the CpG density of the promoters (**Appendix Fig. S2A, B**), suggesting that differences in the CpG density at promoters between species (Deaton and Bird, 2011) may contribute to higher frequency of nucleosome-free molecules at mouse promoters."

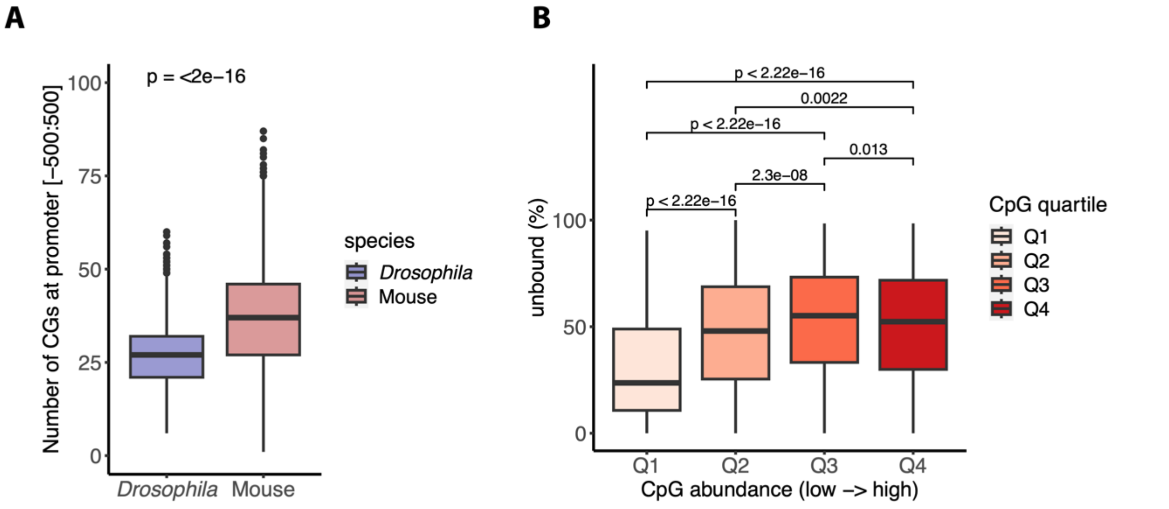

Appendix Figure S2

Appendix Fig. S2. Association between CpG density and promoter accessibility. (A) Comparison of promoter CGs between *Drosophila* and mouse. Number of CGs in the promoter region ([-500:500] bp around the TSS) was counted for each promoter used in the SMF analysis (Drosophila – n = 5,912 promoters; mouse – n = 6,122 promoters). Boxplots represent the distribution of the frequency of the number of CGs at each promoter. The statistical comparisons between groups were performed using Wilcoxon rank-sum test. **(B)** Mouse promoter accessibility as a function of CpG abundance. Mouse promoters were stratified into four quartiles by number of CGs located in the promoter region (Q1 – n = 1,579; Q2 – n = 1,565; Q3 – n = 1,466; Q4 – n = 1,512). Boxplots represent the distribution of the unbound state for promoters in different quartiles. Pairwise comparisons were performed using Wilcoxon rank-sum test. Multiple-hypothesis testing correction was done using Benjamini–Hochberg procedure.

5. In the same line, the promoter state frequencies in 2B and 2D don't match those in 2A and 2C. For example, in Figure 2B, around 37% of Svil gene molecules are categorised as PIC (in red). However, in Figure 2A, the PIC category (in red) looks lower than 10% in all shown genes. Could the authors please explain these discrepancies?

We thank the reviewer for pointing out at this discrepancy in our choice of data representation. The purpose of **Fig. 2A, B** is to represent the general distribution of SMF-derived state frequencies, as a function of Pol II ChIP-seq signal. For each species, promoters were binned based on their Pol II ChIP-seq signal and the average state distribution is plotted for each bin (each vertical bar in **Fig. 2A**, **B**). So, each bar in the barplot is a composite of

multiple promoters. In contrast, the lower panel represent individual example promoters from the highest Pol II ChIP-seq bins. It is thus not expected that they will have exactly the same frequency than the bin they come from. These specific example promoters (*Svil* and *Skp1)* are TATA-containing promoters that show high PIC occupancy. Yet, the majority of promoters are TATA-less, which typically exhibit lower PIC occupancy. As a result, the median PIC state frequency reflects this trend and shows lower overall PIC occupancy.
We realise that this confusion may have arisen from the pointers we had made between the barplots and the individual examples. We have now removed the connector and made explicit statements in the figure legend on how the average are obtained. We hope this clarifies the issue.

6. Determining which component of the Pol II turnover rate changes the most in mouse cells as compared to Drosophila cells could significantly impact the conclusions. Therefore, I suggest assessing the mouse promoter occupancy upon Integrator or p-TEFb perturbations.

We agree that identification of the mechanism underlying the differences in turnover rates between species would be interesting. This would require to perturb either elongation rate (p-TEFb) or premature termination rate (integrator complex) to distinguish their contribution on the difference in the turnover rate between species. These experiments are however not trivial as both complexes are essential. We would therefore need to generate inducible perturbations (i.e. auxin-inducible degron cell lines) in the two cell lines (mESCs and the polyploid S2 cells), and combine these with inhibition of initiation using Triptolide. Upon inhibition, we would need to perform a time-course experiment using either SMF or PRO-seq as a readout for Pol II occupancy. Thus, given the difficulty and the time and resources required, we feel these experiments fall beyond the scope of the current manuscript as they are not essential to support our conclusions. However, we discussed the possible relative contribution of the two processes based existing evidences in the literature. Several lines of evidence suggest that the largest contribution to the turnover of paused Pol II is early termination. These include our own work in S2 cells where we showed that loss of Pol II observed upon triptolide treatment primarily occurs independent of elongation, arguing for a strong contribution of premature termination in Pol II turnover (Krebs et al., 2017). Corroborating with this, rapid Pol II turnover at promoters of human HCT116 cells was unaffected when elongation is blocked (Erickson et al., 2018). More recently, Zimmer *et al.* developed a sequencing assay called Start-TimeLapse-seq (STL-seq) and combined this assay with flavopiridol treatment to measure the rates of Pol II release into elongation and premature termination. The authors found that most promoter-proximal Pol II molecules prematurely terminate (~80%) (Zimmer et al., 2021). Further evidence comes from a live-cell imaging experiment (Steurer et al., 2018), arguing that premature termination is the main contributor to promoter-proximal Pol II turnover. Therefore, although we found that pausing index is lower at mouse promoters, it is more likely that the higher Pol II turnover at mouse promoters could largely come from a higher rate of premature termination in mouse compared to *Drosophila* cells. We have discussed these aspects in the new version of the discussion section:

“This higher turnover rate in mouse cells could be the results of increased premature termination or faster entry into productive elongation. Our datasets do not distinguish between these two potential fates of Pol II and their relative contribution to the differences in Pol II occupancy. Several lines of existing evidence argue that a majority of the Pol II turnover at the paused site occurs through premature termination rather than productive elongation. For instance, chemical inhibition of elongation did not alter the amount and the rate at which Pol II upon inhibition of initiation in S2 cells (Krebs et al., 2017) or human HCT116 cells (Erickson et al., 2018). In agreement with this notion, recent estimates shows that that approximately 80% of promoter-proximal Pol II molecules will be lost by premature termination (Zimmer et al., 2021). Thus, given the prevalence of premature termination, it is likely that it would contribute to the faster Pol II turnover we observed in mouse cells. Yet, these two hypotheses are not mutually exclusive and higher elongation rates may also contribute to the observed differences.
”

Reviewer #2:

Summary
Single Molecule Footprinting has revolutionized our understanding of transcriptional processes, as we now have the ability to detect protein-DNA interactions at single-molecule resolution, without experimental enrichment of sequences of interest, or assaying binding in bulk. In the paper titled, "Mammalian promoters are characterized by low occupancy and high turnover of RNA polymerase II", the authors use SMF to directly quantify Pol II-DNA contacts to identify exact binding frequencies rather than relative occupancy. This study applies SMF to measure Pol II occupancy in both fly and mouse cells, to answer a long-standing question and paradox in the field: Why do major differences exist in metazoan transcription kinetics, although a conserved set of transcription regulators are used with similar binding profiles? The timescales on which transcription occurs is drastically different; on fly promoters, the active and inactive state occur at similar frequencies, on the order of minutes. In contrast, on mouse promoters, very long inactive states (minutes to hours) are interspersed by short active states. By using SMF in Drosophila S2 cells and mouse ESCs, the authors identify that mouse promoters have reduced Pol II occupancy compared to fly promoters, which was previously not appreciated using bulk cell assays. By combining experimental data with mathematical modeling, they also show that this reduced Pol II occupancy in the mouse is consistent with 1) reduced initiation rates, and 2) higher Pol II turnover.

Strengths of this study include integrating experimental results with modeling, comparison of single-molecule approaches to extant bulk sequencing data, and establishing that the results are not a mouse ESC-specific feature, but reproducible across various mouse cell lines. Limitations include the oversimplification that "mammalian" promoters have the features described above, although no human cell lines were assayed. Overall, this study makes significant contributions to our understanding of transcriptional regulation, particularly in

highlighting species-specific differences and challenging the existing model of preloaded polymerases waiting for release. Instead, the findings support a model of rapid cycles of initiation and early termination for RNA Pol II on mouse promoters, which remains to be experimentally validated. Overall, this is a well-written manuscript with compelling data, beautiful figures, and interesting possible experimental follow-ups. There are a few concerns for the authors to address, that would mostly require editing in the text and in the figures.

We appreciate the positive comments from the reviewer and are pleased to read that he finds the results compelling and significant. We would also like to thank the reviewer for the constructive suggestions, which have greatly helped us improve the manuscript.

Major points

The authors convincingly show that mammalian promoters generally have less Pol II occupancy and faster turnover compared to Drosophila. However, it would be valuable to explore the distribution of promoter behaviors more thoroughly across both species. Specifically: Is there a continuum of promoter types in both species, characterized by varying initiation rates and turnover speeds? If so, do the distributions simply differ between species, with mammalian promoters skewed towards lower occupancy and faster turnover? Alternatively, are there distinct categories of promoter behavior that are unique to each species? Do any mammalian promoters exhibit "Drosophila-like" behavior (high occupancy, slower turnover) or vice versa? This analysis would help clarify whether the differences between species represent a fundamental change in promoter regulation or a more subtle shift in the prevalence of certain promoter types.

Thank you for this suggestion to perform a more granular analysis of the distributions of Pol II occupancy and turnover rates at promoters between species. We visualised Pol II occupancy (q) and initiation time (**Fig. EV5**). We observed that the distributions of Pol II occupancy and initiation time are continuous and overlapping between species (**Fig. EV5**). We found that distribution of mouse promoters shifts toward lower Pol II occupancy and higher initiation time. We did not find a distinct group of mouse promoters that exhibit “Drosophila-like” high occupancy, slower turnover, or faster initiation. Of note, the turnover analysis was presented for groups of promoters (**Fig. 5D-E**), as we do not have sufficient data points (5 time points over 2 replicates) to infer the turnover rate for each promoter individually. We conclude that promoter behaviours in both species are continuum, with mouse promoters have a lower Pol II occupancy and higher turnover rate. The new analysis is presented in a supplementary figure **Fig. EV5** and discussed in the manuscript:

“Direct comparison of the distribution of these parameters between species shows that these are partially overlapping with a continuum of values between mouse and *Drosophila* (**Fig. EV5)**, consistent with the idea that these are represent different rates of the same molecular mechanism.”

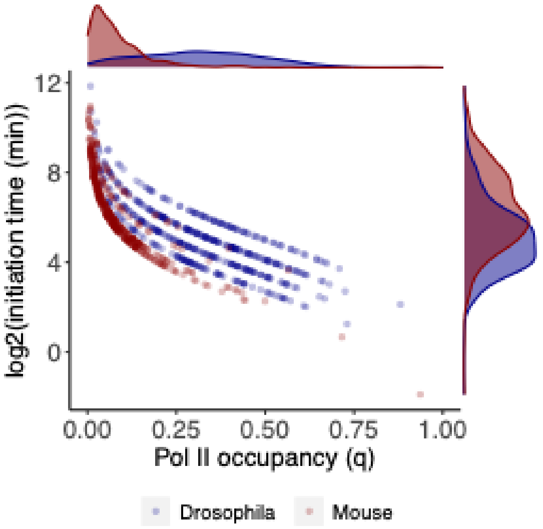

Figure EV5

Fig. EV5. Distribution of Pol II occupancy (q) and initiation time at highly active *Drosophila* and mouse promoters. The x-axis displays Pol II occupancy (q), while y-axis represents log2(initiation time (min)). Each dot represents a promoter. Dot colours correspond to species (blue – *Drosophila*, red – mouse). The density plots on the top and on the right show the distribution of Pol II occupancy (q) and initiation time, respectively.

The conclusion that "This suggests that a low amplitude Pol II footprint is a general feature of mammalian promoters" is a bit too strong (page 5), when 5 different mouse cell lines were used, but no human cell lines were used to determine the generalizability of the findings across mammals. In general, this conclusion will have to be softened throughout the text. In the discussion the authors mention that their data are in agreement with previous studies conducted in human cells. It could be nice to elaborate on this a bit more.

We agree that we only profiled murine cell lines and do not have data in human cell lines. We thus revised the title of the manuscript and the text accordingly. We have also discussed more in regard to other studies as follow:

"Our data suggest that in mouse, Pol II is detected at the paused site in a small fraction of cells. Moreover, we found that Pol II turnover is faster at mouse than *Drosophila* promoters, with half-lives of less than 5 minutes in most highly active mouse promoters. These observed short Pol II half-lives in mouse cells are consistent with values observed human colon cancer cell line HCT116 (2-5 minutes).

This higher turnover rate in mouse cells could be the results of increased premature termination or faster entry into productive elongation. Our datasets do not distinguish between these two potential fates of Pol II and their relative contribution to the differences in Pol II occupancy. Several lines of existing evidence argue that a majority of the Pol II turnover at the paused site occurs through premature termination rather than productive elongation. For instance, chemical inhibition of elongation did not alter the amount and the rate at which Pol II upon inhibition of initiation in S2 cells (Krebs et al., 2017) or human HCT116 cells (Erickson et al., 2018). In agreement with this notion, recent estimates shows

that that approximately 80% of promoter-proximal Pol II molecules will be lost by premature termination (Zimmer et al., 2021). Thus, given the prevalence of premature termination, it is likely that it would contribute to the faster Pol II turnover we observed in mouse cells. Yet, these two hypotheses are not mutually exclusive and higher elongation rates may also contribute to the observed differences."

Minor points:
When introducing their SMF approach or in the results sections, it would be valuable to discuss a bit more the other SMF methods and publications that have emerged since the pioneering Krebs et al. 2017 paper. A brief discussion of how these other approaches compare to the one used here, and whether their findings are consistent with the original Krebs data, would help substantiate the findings described here.

We thank the reviewer for this suggestion. We have now introduced Fiber-seq, which has been successfully employed to study Pol II and nucleosomes at the promoters of *Drosophila* S2 cells (Tullius *et al.*, *Mol. Cell*, 2024), in the introduction section where we introduce SMF. We are also referencing other single molecule genomics techniques that could potentially be used for the same purpose.

"Single Molecule Footprinting (SMF) combines the use of cytosine methyltransferases and bisulfite sequencing to quantify protein-DNA contacts at single molecule resolution across the genome (Kleinendorst et al., 2021; Krebs et al., 2017). Approaches with similar principles such as Fiber-seq (Stergachis et al., 2020), SMAC-seq (Shipony et al., 2020), and SAMOSA (Abdulhay et al., 2020), have been developed to study chromatin accessibility, nucleosome, and protein occupancy across various cell types from yeast to mammals. The single molecule resolution of SMF and these approaches enables the direct quantification of the frequency of promoter occupancy by various DNA binding proteins including transcription factors (TFs), GTFs, and Pol II (**Figure 1A, right**) (Krebs et al., 2017; Sönmezer et al., 2021). We showed that Pol II binding frequency measured by SMF scales with enrichments determined using orthogonal measures such as ChIP-seq or PRO-seq (Krebs et al., 2017). More recently, Tullius et al. employed Fiber-seq, a single-molecule, long-read genomic footprinting using adenine methyltransferase, to study the interplay between Pol II and nucleosome footprints across long DNA molecules in *Drosophila* S2 cell line (Tullius et al., 2024)."

Italicize gene names, such as Svil, Skp1, and Rsrp1, when mentioning them in-text, and in the figures. Other gene names are italicized, but please check that this is consistent throughout the manuscript.

Thank you, we have now italicised all the gene names throughout the manuscript and figures.

"We found that the states were separated in two clusters..." (page 5) is referencing Figure EV2D, but it is not clear how that panel indicates the "active" and "inactive" promoter states. Does this mean the "transcription" cluster is the active, and the "nucleosome" cluster is the

inactive? Perhaps labeling that somehow would make the conclusion and the figure make more sense together.

Yes, the “transcription” cluster is the active, and the “nucleosome” cluster is the inactive. We have now labelled these for clarity.

Figure 2:
Panel A and C show Pol II ChIP-seq rank, but it's not intuitive how it's currently labeled, as there are no values on the x-axis. In the text, the authors indicate that there is "increased promoter activity", presumably as one moves to the right on the x-axis, but perhaps that can be indicated in some way for both panels. In the figure legend, it may be useful if the authors indicate that each vertical line in the plot represents a different promoter.

Thank you, we have clarified this in the figures and the figure legend.

It's unclear why for Svil or Skp1, the state frequencies as shown on the right in B and D don't correspond to the breakdown in the vertical line they're coming from in A and C. For instance, for Svil it looks like it's coming from a region where the "nucleosome" state frequency is ~50%, but for Svil specifically, it's <25%, by eye. Some clarification in the figure and legend would be helpful.

We thank the reviewer for pointing out at this discrepancy in our choice of data representation. The purpose of **Fig. 2A, B** is to represent the general distribution of SMF-derived state frequencies, as a function of Pol II ChIP-seq signal. For each species, promoters were binned based on their Pol II ChIP-seq signal and the average state distribution is plotted for each bin (each vertical bar in **Fig. 2A**, **B**). So, each bar in the barplot is a composite of multiple promoters. In contrast, the lower panel represent individual example promoters from the highest Pol II ChIP-seq bins. It is thus not expected that they will have exactly the same frequency than the bin they come from.
We realise that this confusion may have arisen from the pointers we had made between the barplots and the individual examples. We have now removed the connector and made explicit statements in the figure legend on how the average are obtained. We hope this clarifies the issue.

Please include a legend between panel B and D to indicate what the gray and black lines represent (as done for Figure 3A).
Thank you, fixed.

It would be helpful if the colors of panels E and F are changed to blue and red for Drosophila and Mouse, respectively, as was done for Figure 3B, for the sake of consistency. Please include p-values between TATA-less and TATA promoters for both panels as well, as they are indicated to be statistically significantly different in the text.

Thank you, all fixed.

In the legend, for F, the authors indicate that "the number of promoters... is similar". Are the same exact promoters used, or just the promoter number is the same? Please indicate the n, as is done in (E).

Thank you, the same promoters were used in the analyses in **Fig. 2E** and **Fig. 2F**. We have now indicated the n in the figure legend of **Fig. 2F** as in **Fig. 2E**.

Figure EV2:
Please include a legend for panel B, to indicate what gray and black mean. For "unassigned", it would be helpful if it was a different color throughout the entire manuscript, since the same gray color is used to signify "inaccessible" for the methylation status.

Thank you for the comment, we have changed the colour for “unassigned” to brown.

The authors state that "A reduction in the Pol II footprint was also evident at the TATA-less Rsrp1 promoter" and give specific %, referencing
Figure EV4. It's not clear from the graph in Figure EV4 where the reduction is observed with TRP, since it's so slight in the "Pol II" bin. Is this where the 8% to 1% reduction comes from? Are there any other TATA-less promoters with a greater reduction that can be used as an alternate example?

Thank you for the comment. The challenge when looking at Pol II occupancy at mouse promoters is that the starting occupancy is very low to start with (typically <10%) of the molecules. This problem is even harder for TATA-less promoter that have overall lower Pol II occupancy. Quantifying changes in that low frequency space requires to sequence thousands of molecules. We are therefore limited to a small subset of promoters that were covered in our targeted SMF (35 TATA-containing and 12 TATA-less promoters). We decided to display *Rsrp1* as it has the highest starting Pol II occupancy of our small subset. While the starting occupancy is low, it decreases by almost an order of magnitude (from 8% to 1%). So, while the change is not high in absolute terms, it is high in relative space. We hope that the reviewer agrees with our reasoning and choice of the *Rsrp1* as an example to illustrate a reduced Pol II footprint at the TATA-less promoters.

Figure 6 could use a small change, to make the model clearer: Please move the legend (red/blue arrows) on the left or right of the model, since its current placement suggests that the left side of the model indicates the "mouse" model and the right side the "Drosophila" model.

Thank you for pointing this, the figure is now adjusted accordingly.

Reviewer #3:

MSB-2024-12625
Mammalian promoters are characterised by low occupancy and high turnover of RNA polymerase II
Chatsirisupachai, Moene, Kleinendorst, Kreibich, Molina, Krebs

General comments

I enjoyed reading this paper. It is clearly written; addresses an important and topical problem. It draws important biological insights through an effective integration of quantitative experiments and theory.
The approach is particularly appealing in showing how a clear and simple mathematical model can lead to informative biological conclusions.

We are grateful that the reviewer finds our approach appealing and acknowledges that our paper provides important biological insights. We also greatly appreciate the thoughtful comments from the reviewer, which have substantially improved our manuscript.

As always in exploiting a model, the underlying assumptions are hugely significant. Some of the assumptions that have been made appear rather arbitrary o me and one concern that came to mind is whether the conclusions are sensitive to the choices that have been made. One set of choices have to do with the selection of the 4 DNA sequence bins that define the UP, TATA, POL II and DOWN states (Fig.EV2A).
It does seem unlikely that small changes here would make a significant difference but it still would be helpful to have more justification for the choices made This is important also because these choices influence the interpretation of the resulting 16 regulatory patterns shown in Fig.EV2B. The assumptions made here are more puzzling. In particular, if we use binary notation to signify the regulatory patterns, then 0010 is interpreted as Pol II being present but 1010 is deemed to be unassigned, although it includes 0010 as a sub-pattern. I would have expected monotonicity of patterns, so that what is present in a sub-pattern does not disappear in a pattern that contains it, at least, not without a clear justification. For example, 0011 also contains the sub-pattern 0010 but, in this case, perhaps it could be argued that the 11 is more likely to signify a nucleosome. However, if that is the case, it might only be justified by a closer look at the underlying methylation pattern, rather than the very coarse 0/1 quantisation of the fraction of methylated sites that is used to define the state of each bin. I do not think the choices made in Fig.EV2B have been adequately justified. Since these choices are fundamental to the model that follows, I would encourage the authors to clarify their reasoning.

We thank the reviewer for raising this important point. We agree with the reviewer that the supervised analysis of the data using bins is a pragmatic solution that rely on assumptions that can indeed influence the analysis outcome. We have now clarified in the manuscript the

reasoning behind the selection of the bins and their biological interpretation based on prior knowledge. In a nutshell, the selection of the four DNA sequence bins was based on the position of promoter elements where transcription machinery binds at the core promoter, namely the PIC at the TATA site and Pol II at the pausing site, based on electron microscopy and in vitro footprinting (Cianfrocco *et al.*, *Cell*, 2013; Lee *et al.*, *Mol. Cell Biol.*, 2008). The inclusion of upstream (UP) and downstream (DOWN) bins was intended to better separate PIC/Pol II footprints from a broader and more continuous nucleosome footprint, under the notion that promoters should be "accessible" or "nucleosome-free" during active transcription period. As illustrated in the composite plot from top 5% highly active TATA-containing promoters (**Fig. EV3A**), the position of the UP bin is absent of footprint. This observation is further supported by the single-site example, such as **Fig. 2B, D** and **Fig. 3A**, where we can observe the differences in single-molecule methylation patterns between DNA molecule that are accessible and those that are nucleosome-bound at the UP bin position. Thus, while it could be argued that the molecule containing the pattern "1010" may have Pol II bound at the pausing site, it could also be interpreted as a closed promoter.
Our decision to include the downstream bin to distinguish Pol II footprints from nucleosomes can be exemplified by the cases of "0010" and "0011," which we classified as "Pol II" and "nucleosome," respectively. In general, the +1 nucleosome shifts further downstream of the TSS in the presence of Pol II at the pausing site (Gilchrist et al., 2010). This is further confirmed by a recent work using Fiber-seq to quantify Pol II, PIC, and nucleosome footprints on a long DNA molecule (Tullius et al., 2024). The authors observed that the presence of Pol II footprint at the pausing site is associated with a ~15 bp downstream shift in the +1 nucleosome. When Pol II footprint is present, the region [55:75] downstream of the TSS is accessible, while the nucleosome footprint usually starts at >75 bp (Tullius et al., 2024). Thus, the protected downstream bin in our setting likely indicates the presence of nucleosome. Therefore, we interpreted the "0011" pattern as "nucleosome".

We have better clarified the reason of choosing these four bins in the Method section of the manuscript as follows:

"**SMF – Single molecule sorting**

For single molecule analysis, the molecular classifier from our previous study (Krebs et al., 2017) was adapted to adequately assess the presence of promoter-proximal footprints at mouse promoters. In brief, four bins of interest were defined relative to the TSS, based on the expected position of promoter elements where transcription machinery binds at the core promoter, including the PIC at the TATA site and Pol II at the Pol II pausing site (Cianfrocco et al., 2013; Lee et al., 2008). We next included the upstream and the downstream bins to better separate PIC and Pol II footprint from a broader and more continuous nucleosome footprints, under the assumption that promoters should be "accessible" or "nucleosome-free" during active transcription period. In total, the four bins include: upstream [-58:-43], TATA-box [-36:-22], Pol II [28:47], and downstream [54:69] (**Figure EV2A**)."

Aside from this point, the comments I have are minor. I believe this paper makes an important contribution to our understanding of gene regulation and it is pleasure to recommend it for publication.

Jeremy Gunawardena

Specific comments

1. Page 4, bottom para. I suggest annotating Figs.1E and 1F to show which peaks correspond to the PIC, Pol II pausing and +1 nucleosome. I found myself moving back and forth between the text and the figure to keep track of this and ended up scribbling a note on the figure.

Thank you for pointing this out. We have now edited the figures as suggested.

2. Page 5, para 2. See the general comment above on the choices made in Fig.EV2.

Thank you, we have now clarified this in the respond to comment above.

3. Page 8, first line. The derivation of the sigmoid function could be simpler that what is given in the Methods (pages 17 & 18). The differential equations are not needed. From the model in Fig.4A, because there is no net flux through a pipeline, since it has nowhere to go at the ends, it must be that, in steady state, k_i . P_open = k_t . P_Pol II, so that P_open / P_Pol II = k_t / k_i = r. Hence, for the quantity being considered, P_Pol II / (P_open + P_Pol II) = 1 / (1 + r). This algebra is so simple that I would encourage the authors to put it in the main text, so that readers can gain an intuition for the approach.

Thank you very much for this pertinent observation. We fully agree that using detailed balance leads to the same results in a simpler and more straightforward manner. We have indicated this in the main text and the Methods. We have kept the former derivation for completeness and have noted that for systems where transient dynamics may be important, for instance, gene activation or deactivation upon induction, obtaining the full time-dependent solution may be necessary.

4. page 8, para 3, line 1. "specie" --> species

Thanks for the comment, fixed.

5. page 21, Fig.4 caption. The Open pattern is defined as "the promoter is unbound by either nucleosome or Pol II" and later as "unbound and PIC states from SMF". The second is more accurate than the first and should be used exclusively. There are 3 plots in Fig.4B but the one on the right is not described. It would be clearer to say something like "the plot at the top shows the distribution of r on the data and the plot on the right shows the distribution of q on the data".

Thank you, we now exclusively used the definition of the Open pattern as “unbound and PIC states from SMF”. We also added the description for the plot on the right as pointed out by the reviewer.

6. page 23, lines -3, -5. "log_2 fold change" with respect to what? Presumably the PRO-seq signal at time 0 but it would be better to say so.

Yes, this is log2 fold change with respect to PRO-seq signal at time 0. We have now clarified this.

Figure 4. The vertical arrow entering the green region annotated with k_i and the horizontal arrow leaving the purple region annotated with k_t are in the wrong place. The annotations should be placed instead on the pair of arrows on the boundary between the green and purple regions.

Thank you, we have now corrected the figure as the reviewer pointed out.

Figure EV5. The horizontal alignment of the "r = xxxx" in panel A is bad in several of the plots. Replace "Rep 1" and "Rep 2" in panels C and D with the corresponding units

Thank you, these are now fixed. This figure is now Appendix Figure S3 in the revised manuscript.

References

Abdulhay, N.J., McNally, C.P., Hsieh, L.J., Kasinathan, S., Keith, A., Estes, L.S., Karimzadeh, M., Underwood, J.G., Goodarzi, H., Narlikar, G.J., Ramani, V., 2020. Massively multiplex single-molecule oligonucleosome footprinting. eLife 9, e59404. https://doi.org/10.7554/eLife.59404

Cianfrocco, M.A., Kassavetis, G.A., Grob, P., Fang, J., Juven-Gershon, T., Kadonaga, J.T., Nogales, E., 2013. Human TFIID Binds to Core Promoter DNA in a Reorganized Structural State. Cell 152, 120–131. https://doi.org/10.1016/j.cell.2012.12.005

Day, D.S., Zhang, B., Stevens, S.M., Ferrari, F., Larschan, E.N., Park, P.J., Pu, W.T., 2016. Comprehensive analysis of promoter-proximal RNA polymerase II pausing across mammalian cell types. Genome Biol 17, 120. https://doi.org/10.1186/s13059-016-0984-2

Deaton, A.M., Bird, A., 2011. CpG islands and the regulation of transcription. Genes Dev. 25, 1010–1022. https://doi.org/10.1101/gad.2037511

Erickson, B., Sheridan, R.M., Cortazar, M., Bentley, D.L., 2018. Dynamic turnover of paused Pol II complexes at human promoters. Genes Dev. 32, 1215–1225. https://doi.org/10.1101/gad.316810.118

Fenouil, R., Cauchy, P., Koch, F., Descostes, N., Cabeza, J.Z., Innocenti, C., Ferrier, P., Spicuglia, S., Gut, M., Gut, I., Andrau, J.-C., 2012. CpG islands and GC content dictate nucleosome depletion in a transcription-independent manner at mammalian promoters. Genome Res. 22, 2399–2408. https://doi.org/10.1101/gr.138776.112

Gilchrist, D.A., Dos Santos, G., Fargo, D.C., Xie, B., Gao, Y., Li, L., Adelman, K., 2010. Pausing of RNA Polymerase II Disrupts DNA-Specified Nucleosome Organization to

Enable Precise Gene Regulation. Cell 143, 540–551. https://doi.org/10.1016/j.cell.2010.10.004
Kleinendorst, R.W.D., Barzaghi, G., Smith, M.L., Zaugg, J.B., Krebs, A.R., 2021. Genome-wide quantification of transcription factor binding at single-DNA-molecule resolution using methyl-transferase footprinting. Nat Protoc. https://doi.org/10.1038/s41596-021-00630-1
Krebs, A.R., Imanci, D., Hoerner, L., Gaidatzis, D., Burger, L., Schübeler, D., 2017. Genome-wide Single-Molecule Footprinting Reveals High RNA Polymerase II Turnover at Paused Promoters. Molecular Cell 67, 411-422.e4. https://doi.org/10.1016/j.molcel.2017.06.027
Kreibich, E., Kleinendorst, R., Barzaghi, G., Kaspar, S., Krebs, A.R., 2023. Single-molecule footprinting identifies context-dependent regulation of enhancers by DNA methylation. Molecular Cell 83, 787-802.e9. https://doi.org/10.1016/j.molcel.2023.01.017
Lee, C., Li, X., Hechmer, A., Eisen, M., Biggin, M.D., Venters, B.J., Jiang, C., Li, J., Pugh, B.F., Gilmour, D.S., 2008. NELF and GAGA Factor Are Linked to Promoter-Proximal Pausing at Many Genes in *Drosophila*. Molecular and Cellular Biology 28, 3290–3300. https://doi.org/10.1128/MCB.02224-07
Shipony, Z., Marinov, G.K., Swaffer, M.P., Sinnott-Armstrong, N.A., Skotheim, J.M., Kundaje, A., Greenleaf, W.J., 2020. Long-range single-molecule mapping of chromatin accessibility in eukaryotes. Nat Methods 17, 319–327. https://doi.org/10.1038/s41592-019-0730-2
Sönmezer, C., Kleinendorst, R., Imanci, D., Barzaghi, G., Villacorta, L., Schübeler, D., Benes, V., Molina, N., Krebs, A.R., 2021. Molecular Co-occupancy Identifies Transcription Factor Binding Cooperativity In Vivo. Molecular Cell 81, 255-267.e6. https://doi.org/10.1016/j.molcel.2020.11.015
Stergachis, A.B., Debo, B.M., Haugen, E., Churchman, L.S., Stamatoyannopoulos, J.A., 2020. Single-molecule regulatory architectures captured by chromatin fiber sequencing. Science 368, 1449–1454. https://doi.org/10.1126/science.aaz1646
Steurer, B., Janssens, R.C., Geverts, B., Geijer, M.E., Wienholz, F., Theil, A.F., Chang, J., Dealy, S., Pothof, J., Van Cappellen, W.A., Houtsmuller, A.B., Marteijn, J.A., 2018. Live-cell analysis of endogenous GFP-RPB1 uncovers rapid turnover of initiating and promoter-paused RNA Polymerase II. Proc. Natl. Acad. Sci. U.S.A. 115. https://doi.org/10.1073/pnas.1717920115
Tullius, T.W., Isaac, R.S., Dubocanin, D., Ranchalis, J., Churchman, L.S., Stergachis, A.B., 2024. RNA polymerases reshape chromatin architecture and couple transcription on individual fibers. Molecular Cell 0. https://doi.org/10.1016/j.molcel.2024.08.013
Zimmer, J.T., Rosa-Mercado, N.A., Canzio, D., Steitz, J.A., Simon, M.D., 2021. STL-seq reveals pause-release and termination kinetics for promoter-proximal paused RNA polymerase II transcripts. Molecular Cell 81, 4398-4412.e7. https://doi.org/10.1016/j.molcel.2021.08.019

14th Feb 2025

Manuscript Number: MSB-2024-12625R
Title: Mouse promoters are characterised by low occupancy and high turnover of RNA polymerase II

Dear Dr. Krebs,

Thank you for the submission of your revised manuscript to Molecular Systems Biology. We have now received the enclosed reports from the referees that were asked to re-assess it. As you will see the reviewers are now globally supportive and I am pleased to inform you that we will be able to accept your manuscript pending the following final amendments:

1) Please update the README file on Github for the model/code with practical use instructions for potential future users.
2) Please format the Data availability section according to the example below, ensuring that direct links to the datasets are included:
"The datasets and computer code produced in this study are available in the following databases:
- Chip-Seq data: Gene Expression Omnibus GSE46748 (https://www.ncbi.nlm.nih.gov/geo/query/acc.cgi?acc=GSE46748)
- Modeling computer scripts: GitHub (https://github.com/SysBioChalmers/GECKO/releases/tag/v1.0)
- [data type]: [full name of the resource] [accession number/identifier] ([doi or URL or identifiers.org/DATABASE:ACCESSION])"
3) Code: Please include a README file on Github with practical use instructions for potential future users of your code.
4) Please rename "Conflict of Interest" to "Disclosure and competing interests statement". We updated our journal's competing interests policy in January 2022 and request authors to consider both actual and perceived competing interests. Please review the policy https://www.embopress.org/competing-interests and update your competing interests if necessary.
5) Author contributions: Please remove it from the manuscript and specify author contributions in our submission system. CRediT has replaced the traditional author contributions section because it offers a systematic machine-readable author contributions format that allows for more effective research assessment. You are encouraged to use the free text boxes beneath each contributing author's name to add specific details on the author's contribution. More information is available in our guide to authors:
https://www.embopress.org/page/journal/17574684/authorguide#authorshipguidelines
6) References: Please remove the DOIs from the references. Please check "Author Guidelines" for more information.
https://www.embopress.org/page/journal/17574684/authorguide#referencesformat
7) Our journal encourages inclusion of *data citations in the reference list* to directly cite datasets that were re-used and obtained from public databases. Data citations in the article text are distinct from normal bibliographical citations and should directly link to the database records from which the data can be accessed. In the main text, data citations are formatted as follows: "Data ref: Smith et al, 2001" or "Data ref: NCBI Sequence Read Archive PRJNA342805, 2017". In the Reference list, data citations must be labeled with "[DATASET]". A data reference must provide the database name, accession number/identifiers and a resolvable link to the landing page from which the data can be accessed at the end of the reference. Further instructions are available at .
8) In the Methods, please take care of the following:
- Cell lines: Please be sure to include a sentence in the Methods as to whether or not the cell lines were recently authenticated and tested for mycoplasma contamination and to update the Author Checklist with this information.
- Please ensure that a statement on whether or not blinding was done is included in the Methods even if no blinding was done. Please also be sure to update the Author Checklist with this information and where it can be found in the manuscript.
9) Please remove the Reagents and Tools table from the main manuscript file and upload it as a separate file choosing the file type "Reagent Table". You can download and fill our Reagents and Tools Table template (.docx) from our author guidelines: https://www.embopress.org/page/journal/14693178/authorguide#structuredmethods.
10) Please place individual sections of the manuscript in the following order: Title page - Abstract & Keywords - Introduction - Results - Discussion - Methods - Data Availability - Acknowledgements - Disclosure and Competing Interests Statement - References - Figure Legends - Expanded View Figure Legends.
11) For the figures and figure legends, please take care of the following:
- Please note that the exact p values are not provided in the legends of figures 2E, F; supplementary figures 1B, 2A, B.
- Please note that the box plots need to be defined in terms of minima, maxima, centre, bounds of box and whiskers, and percentile in the legends of figures 2E, F; 3B, 5A, EV4 B; supplementary figures 1B, 2A, B.
12) Datasets: Datasets EV1-8 need to be uploaded as individual files, not in one Excel file; their legends should be removed from the main manuscript file and included in a separate tab/sheet in the respective Excel file. Please also double check the callouts for the datasets - there are callouts for Datasets EV9-EV10 but only 8 datasets uploaded.
13) Tables: Tables EV1-EV2 should be removed from the manuscript file and unzipped, and then uploaded as individual Expanded View Content (was Supplementary Information) files.
14) Funding: Please add the information on funders currently in the comments box in our manuscript submission system to the "More Funders" option.
15) Synopsis:
- Synopsis image: Please provide a graphic that summarises the main findings of the manuscript on a glance and upload it as a high-resolution jpeg file 550 pixels wide x (300-600) pixels high.

- Synopsis text: Please provide a short standfirst (maximum of 300 characters, including space), limit the bullet points to max. 5 and upload it as a separate .doc file. Please write the bullet points to summarise the key NEW findings. They should be designed to be complementary to the abstract - i.e. not repeat the same text. We encourage inclusion of key acronyms and quantitative information (maximum of 30 words / bullet point). Please use the passive voice.
- Please check your synopsis text and image before submission with your revised manuscript. Please be aware that in the proof stage minor corrections only are allowed (e.g., typos).
16) As part of the EMBO Publications transparent editorial process initiative (see our policy here: https://www.embopress.org/transparent-process#Review_Process), Molecular Systems Biology will publish online a Peer Review File (PRF) to accompany accepted manuscripts. This file will be published in conjunction with your paper and will include the anonymous referee reports, your point-by-point response and all pertinent correspondence relating to the manuscript. Let us know whether you agree with the publication of the PRF and as here, if you want to remove or not any figures from it prior to publication. Please note that the Authors checklist will be published at the end of the PRF.
17) After your paper is published, we will promote it on social media. If you have any handles or hashtags for Bluesky or X you would like included, please let us know.
18) Please provide a point-by-point letter INCLUDING my comments and your detailed responses (as Word file).

I look forward to reading a new revised version of your manuscript as soon as possible.

Yours sincerely,

Poonam Bheda, PhD
Scientific Editor
Molecular Systems Biology

Reviewer #1:

The authors addressed all the points raised by the reviewers with clear results and clarifications, which have now been incorporated in the manuscript. So, the manuscript is suitable for publication without further modifications.

Reviewer #2:

The authors addressed all concerns. Overall, this is a well-written manuscript with compelling data that will be of general interest, and provides evidence of different transcriptional kinetics across species, which has been a longstanding area of interest in the transcription field.

Reviewer #3:

The revised manuscript has carefully addressed the questions that were raised. It is clear from the response that assigning biological meaning to the 4-bit bin patterns depends on substantial prior knowledge of the literature and may therefore be somewhat tentative and subject to reinterpretation in the future. The important point is to make these underlying assumptions clear and transparent, which the authors have now done.

Authors' response to the Editor's comments

Manuscript: MSB-2024-12625R
Title: Mouse promoters are characterised by low occupancy and high turnover of RNA polymerase II

Dear Dr Poonam Bheda,

We were glad to read that we have addressed all concerns from the reviewers and that our manuscript has been accepted for publication in *Molecular Systems Biology*. Please find below our point-by-point response to the comments from you. In addition, we also made minor changes as follows:

1. We noticed that we mislabelled the y-axis of the Appendix Fig. S2A as "Number of CGs at promoter [-500:500]", which has now been corrected to "Number of CGs at promoter [-250:250]".
2. We recently came across a relevant, newly published article that comprehensively characterises the role of Pol II early termination in gene expression regulation (Lysakovskaia *et al.*, 2025). Accordingly, we have modified our Discussion to include the following statement:
   "Comprehensive measure of Pol II dynamics recently showed that promoter-proximal termination plays a key role in controlling dynamic transcriptional changes during transdifferentiation of human B-cells (Lysakovskaia *et al*, 2025), revealing that early termination is a prevalent mechanism in gene expression control."

We hope that you will find the revised version of the manuscript appropriate for publication.

Best regards,

Arnaud Krebs

Editor:
1) Please update the README file on Github for the model/code with practical use instructions for potential future users.

The README file on our GitHub has been updated.

2) Please format the Data availability section according to the example below, ensuring that direct links to the datasets are included:
"The datasets and computer code produced in this study are available in the following databases:
- Chip-Seq data: Gene Expression Omnibus GSE46748 (https://www.ncbi.nlm.nih.gov/geo/query/acc.cgi?acc=GSE46748)
- Modeling computer scripts: GitHub (https://github.com/SysBioChalmers/GECKO/releases/tag/v1.0)

- [data type]: [full name of the resource] [accession number/identifier] ([doi or URL or identifiers.org/DATABASE:ACCESSION])"

We have now provided the direct link to the datasets in the Data availability section.

3) Code: Please include a README file on Github with practical use instructions for potential future users of your code.

The README file on our GitHub has been updated.

4) Please rename "Conflict of Interest" to "Disclosure and competing interests statement". We updated our journal's competing interests policy in January 2022 and request authors to consider both actual and perceived competing interests. Please review the policy https://www.embopress.org/competing-interests and update your competing interests if necessary.

We have renamed the "Conflict of Interest" to "Disclosure and competing interests statement".

5) Author contributions: Please remove it from the manuscript and specify author contributions in our submission system. CRediT has replaced the traditional author contributions section because it offers a systematic machine-readable author contributions format that allows for more effective research assessment. You are encouraged to use the free text boxes beneath each contributing author's name to add specific details on the author's contribution. More information is available in our guide to authors: https://www.embopress.org/page/journal/17574684/authorguide#authorshipguidelines

The Author contributions section has now been removed.

6) References: Please remove the DOIs from the references. Please check "Author Guidelines" for more information. https://www.embopress.org/page/journal/17574684/authorguide#referencesformat

We have now formated the References according to the journal style.

7) Our journal encourages inclusion of *data citations in the reference list* to directly cite datasets that were re-used and obtained from public databases. Data citations in the article text are distinct from normal bibliographical citations and should directly link to the database records from which the data can be accessed. In the main text, data citations are formatted as follows: "Data ref: Smith et al, 2001" or "Data ref: NCBI Sequence Read Archive PRJNA342805, 2017". In the Reference list, data citations must be labeled with "[DATASET]". A data reference must provide the database name, accession number/identifiers and a resolvable link to the landing page from which the data can be accessed at the end of the reference. Further instructions are available at https://www.embopress.org/page/journal/17574684/authorguide#referencesformat.

Data citations were added in the revised manuscript.

8) In the Methods, please take care of the following:
- Cell lines: Please be sure to include a sentence in the Methods as to whether or not the cell

lines were recently authenticated and tested for mycoplasma contamination and to update the Author Checklist with this information.
- Please ensure that a statement on whether or not blinding was done is included in the Methods even if no blinding was done. Please also be sure to update the Author Checklist with this information and where it can be found in the manuscript.

The cell lines were not recently authenticated and tested for mycoplasma. We have added the following statement in the Methods section: “The cells used in this study were not recently authenticated or tested for mycoplasma contamination.”. We have also updated the Author Checklist.

The blinding statement was added in the Methods section as follows: “No blinding was performed in this study.”

9) Please remove the Reagents and Tools table from the main manuscript file and upload it as a separate file choosing the file type "Reagent Table". You can download and fill our Reagents and Tools Table template (.docx) from our author guidelines: https://www.embopress.org/page/journal/14693178/authorguide#structuredmethods.

The Reagent Table has been uploaded as a separate file.

10) Please place individual sections of the manuscript in the following order: Title page - Abstract & Keywords - Introduction - Results - Discussion - Methods - Data Availability - Acknowledgements - Disclosure and Competing Interests Statement - References - Figure Legends - Expanded View Figure Legends.

The manuscript has now been structured according to the suggested order.

11) For the figures and figure legends, please take care of the following:
- Please note that the exact p values are not provided in the legends of figures 2E, F; supplementary figures 1B, 2A, B.
- Please note that the box plots need to be defined in terms of minima, maxima, centre, bounds of box and whiskers, and percentile in the legends of figures 2E, F; 3B, 5A, EV4 B; supplementary figures 1B, 2A, B.

We have now provided exact p-values instead of N.S. (not significant) in Figure 2E. We note that $p < 2.22e-16$ is the smallest p-value one would get from R statistical software due to its numerical precision limit.
The figure legends of all figures showing box plots were updated with the following statement: “The middle line of the box represents the median. The box displays interquartile range (IQR), 25th to 75th percentile. Whiskers represent a distance of 1.5 × IQR.”

12) Datasets: Datasets EV1-8 need to be uploaded as individual files, not in one Excel file; their legends should be removed from the main manuscript file and included in a separate tab/sheet in the respective Excel file. Please also double check the callouts for the datasets - there are callouts for Datasets EV9-EV10 but only 8 datasets uploaded.

We have now uploaded Datasets EV1-8 as individual files. Many thanks for spotting the callout mistake in the manuscript, which has now been corrected to Datasets EV7-EV8.

13) Tables: Tables EV1-EV2 should be removed from the manuscript file and unzipped, and then uploaded as individual Expanded View Content (was Supplementary Information) files.

Tables EV1-EV2 have now been uploaded as individual files.

14) Funding: Please add the information on funders currently in the comments box in our manuscript submission system to the "More Funders" option.

We have added the funding information on the “More Funders” option in the submission system.

15) Synopsis:
- Synopsis image: Please provide a graphic that summarises the main findings of the manuscript on a glance and upload it as a high-resolution jpeg file 550 pixels wide x (300-600) pixels high.
- Synopsis text: Please provide a short standfirst (maximum of 300 characters, including space), limit the bullet points to max. 5 and upload it as a separate .doc file. Please write the bullet points to summarise the key NEW findings. They should be designed to be complementary to the abstract - i.e. not repeat the same text. We encourage inclusion of key acronyms and quantitative information (maximum of 30 words / bullet point). Please use the passive voice.
- Please check your synopsis text and image before submission with your revised manuscript. Please be aware that in the proof stage minor corrections only are allowed (e.g., typos).

Please find uploaded Synopsis text and image.

16) As part of the EMBO Publications transparent editorial process initiative (see our policy here: https://www.embopress.org/transparent-process#Review_Process), Molecular Systems Biology will publish online a Peer Review File (PRF) to accompany accepted manuscripts. This file will be published in conjunction with your paper and will include the anonymous referee reports, your point-by-point response and all pertinent correspondence relating to the manuscript. Let us know whether you agree with the publication of the PRF and as here, if you want to remove or not any figures from it prior to publication. Please note that the Authors checklist will be published at the end of the PRF.

We agree to publish the PRF together with our manuscript.

17) After your paper is published, we will promote it on social media. If you have any handles or hashtags for Bluesky or X you would like included, please let us know.

Please include the following handles when promote the manuscript on social media.
- Bluesky: @kasitc.bsky.social, @molinalab.bsky.social, @arnaudkr.bsky.social
- X: @KasitC10, @ChristineMoene, @molina_lab

18) Please provide a point-by-point letter INCLUDING my comments and your detailed responses (as Word file).

13th Mar 2025
Manuscript number: MSB-2024-12625RR
Title: Mouse promoters are characterised by low occupancy and high turnover of RNA polymerase II

Dear Dr. Krebs,

Congratulations on an excellent manuscript, I am pleased to inform you that your manuscript has been accepted for publication in Molecular Systems Biology. Thank you for your comprehensive response to referee concerns. It has been a pleasure to work with you to get this to the acceptance stage.

Your manuscript will be processed for publication by EMBO Press. It will be copy edited and you will receive page proofs prior to publication. Please note that you will be contacted by Springer Nature Author Services to complete licensing and payment information.

You may qualify for financial assistance for your publication charges - either via a Springer Nature fully open access agreement or an EMBO initiative. Check your eligibility: https://www.embopress.org/page/journal/17444292/authorguide#chargesguide

Should you be planning a Press Release on your article, please get in contact with embo_production@springernature.com as early as possible in order to coordinate publication and release dates.

If you have any questions, please do not hesitate to contact the Editorial Office. Thank you for your contribution to Molecular Systems Biology.

Yours sincerely,

Sincerely,

Poonam Bheda, PhD
Scientific Editor
Molecular Systems Biology

>>> Please note that it is Molecular Systems Biology policy for the transcript of the editorial process (containing referee reports and your response letter) to be published as an online supplement to each paper. If you do NOT want this, you will need to inform the Editorial Office via email immediately. More information is available here: https://www.embopress.org/transparent-process#Review_Process

EMBO Press Author Checklist

Corresponding Author Name: Arnaud Krebs
Journal Submitted to: Molecular Systems Biology
Manuscript Number: MSB-2024-12625

USEFUL LINKS FOR COMPLETING THIS FORM
The EMBO Journal - Author Guidelines
EMBO Reports - Author Guidelines
Molecular Systems Biology - Author Guidelines
EMBO Molecular Medicine - Author Guidelines

Reporting Checklist for Life Science Articles (updated January
This checklist is adapted from Materials Design Analysis Reporting (MDAR) Checklist for Authors. MDAR establishes a minimum set of requirements in transparent reporting in the life sciences (see Statement of Task: 10.31222/osf.io/9sm4x). Please follow the journal's guidelines in preparing your
Please note that a copy of this checklist will be published alongside your article.

Abridged guidelines for figures
1. Data
The data shown in figures should satisfy the following conditions:
- ➞ the data were obtained and processed according to the field's best practice and are presented to reflect the results of the experiments in an accurate and unbiased manner.
- ➞ ideally, figure panels should include only measurements that are directly comparable to each other and obtained with the same assay.
- ➞ plots include clearly labeled error bars for independent experiments and sample sizes. Unless justified, error bars should not be shown for technical
- ➞ if n<5, the individual data points from each experiment should be plotted. Any statistical test employed should be justified.
- ➞ Source Data should be included to report the data underlying figures according to the guidelines set out in the authorship guidelines on Data

2. Captions
Each figure caption should contain the following information, for each panel where they are relevant:
- ➞ a specification of the experimental system investigated (eg cell line, species name).
- ➞ the assay(s) and method(s) used to carry out the reported observations and measurements.
- ➞ an explicit mention of the biological and chemical entity(ies) that are being measured.
- ➞ an explicit mention of the biological and chemical entity(ies) that are altered/varied/perturbed in a controlled manner.
- ➞ the exact sample size (n) for each experimental group/condition, given as a number, not a range;
- ➞ a description of the sample collection allowing the reader to understand whether the samples represent technical or biological replicates (including how many animals, litters, cultures, etc.).
- ➞ a statement of how many times the experiment shown was independently replicated in the laboratory.
- ➞ definitions of statistical methods and measures:
  - common tests, such as t-test (please specify whether paired vs. unpaired), simple χ2 tests, Wilcoxon and Mann-Whitney tests, can be unambiguously identified by name only, but more complex techniques should be described in the methods section;
  - are tests one-sided or two-sided?
  - are there adjustments for multiple comparisons?
  - exact statistical test results, e.g., P values = x but not P values < x;
  - definition of 'center values' as median or average;
  - definition of error bars as s.d. or s.e.m.

Please complete ALL of the questions below.
Select "Not Applicable" only when the requested information is not relevant for your study.

Materials

Newly Created Materials	Information included in the manuscript?	In which section is the information available? (Reagents and Tools Table, Materials and Methods, Figures, Data Availability Section)
New materials and reagents need to be available; do any restrictions apply?	Not Applicable	

Antibodies	Information included in the manuscript?	In which section is the information available? (Reagents and Tools Table, Materials and Methods, Figures, Data Availability Section)
For **antibodies** provide the following information:<br>- Commercial antibodies: RRID (if possible) or supplier name, catalogue number and or/clone number<br>- Non-commercial: RRID or citation	Not Applicable	

DNA and RNA sequences	Information included in the manuscript?	In which section is the information available? (Reagents and Tools Table, Materials and Methods, Figures, Data Availability Section)
**Short novel DNA or RNA including primers, probes**: provide the sequences.	Yes	Reagents and Tools

Cell materials	Information included in the manuscript?	In which section is the information available? (Reagents and Tools Table, Materials and Methods, Figures, Data Availability Section)
**Cell lines:** Provide species information, strain. Provide accession number in repository **OR** supplier name, catalog number, clone number, and/**OR** RRID.	Yes	Reagents and Tools
**Primary cultures:** Provide species, strain, sex of origin, genetic modification status.	Not Applicable	
Report if the cell lines were recently **authenticated** (e.g., by STR profiling) and tested for mycoplasma contamination.	Yes	Materials and Methods

Experimental animals	Information included in the manuscript?	In which section is the information available? (Reagents and Tools Table, Materials and Methods, Figures, Data Availability Section)
**Laboratory animals or Model organisms:** Provide species, strain, sex, age, genetic modification status. Provide accession number in repository **OR** supplier name, catalog number, clone number, **OR** RRID.	Not Applicable	
**Animal observed in or captured from the field:** Provide species, sex, and age where possible.	Not Applicable	
Please detail **housing and husbandry conditions**.	Not Applicable	

Plants and microbes	Information included in the manuscript?	In which section is the information available? (Reagents and Tools Table, Materials and Methods, Figures, Data Availability Section)
**Plants:** provide species and strain, ecotype and cultivar where relevant, unique accession number if available, and source (including location for collected wild specimens).	Not Applicable	
**Microbes:** provide species and strain, unique accession number if available, and source.	Not Applicable	

Human research participants	Information included in the manuscript?	In which section is the information available? (Reagents and Tools Table, Materials and Methods, Figures, Data Availability Section)
If collected and within the bounds of privacy constraints report on age, sex and gender or ethnicity for all study participants.	Not Applicable	

Core facilities	Information included in the manuscript?	In which section is the information available? (Reagents and Tools Table, Materials and Methods, Figures, Data Availability Section)
If your work benefited from core facilities, was their service mentioned in the acknowledgments section?	Yes	Acknowledgements

Design

Study protocol	Information included in the manuscript?	In which section is the information available? (Reagents and Tools Table, Materials and Methods, Figures, Data Availability Section)
If study protocol has been **pre-registered, provide DOI in the manuscript**. For clinical trials, provide the trial registration number **OR** cite DOI.	Not Applicable	
Report the **clinical trial registration number** (at ClinicalTrials.gov or equivalent), where applicable.	Not Applicable	

Laboratory protocol	Information included in the manuscript?	In which section is the information available? (Reagents and Tools Table, Materials and Methods, Figures, Data Availability Section)
Provide DOI OR other citation details if **external detailed step-by-step protocols** are available.	Yes	Material and Methods, References

Experimental study design and statistics	Information included in the manuscript?	In which section is the information available? (Reagents and Tools Table, Materials and Methods, Figures, Data Availability Section)
Include a statement about **sample size** estimate even if no statistical methods were used.	Not Applicable	
Were any steps taken to minimize the effects of subjective bias when allocating animals/samples to treatment (e.g. **randomization procedure**)? If yes, have they been described?	Not Applicable	
Include a statement about **blinding** even if no blinding was done.	Yes	Material and Methods
Describe **inclusion/exclusion criteria** if samples or animals were excluded from the analysis. Were the criteria pre-established?<br><br>If sample or data points were omitted from analysis, report if this was due to attrition or intentional exclusion and provide justification.	Not Applicable	
For every figure, are **statistical tests** justified as appropriate? Do the data meet the assumptions of the tests (e.g., normal distribution)? Describe any methods used to assess it. Is there an estimate of variation within each group of data? Is the variance similar between the groups that are being statistically compared?	Yes	Figure legends

Sample definition and in-laboratory replication	Information included in the manuscript?	In which section is the information available? (Reagents and Tools Table, Materials and Methods, Figures, Data Availability Section)
In the figure legends: state number of times the experiment was **replicated** in laboratory.	Yes	Figure legends
In the figure legends: define whether data describe **technical or biological replicates**.	Yes	Figure legends

Ethics

Ethics	Information included in the manuscript?	In which section is the information available? (Reagents and Tools Table, Materials and Methods, Figures, Data Availability Section)
Studies involving **human participants**: State details of **authority granting ethics approval** (IRB or equivalent committee(s), provide reference number for approval.	Not Applicable	
Studies involving **human participants**: Include a statement confirming that **informed consent** was obtained from all subjects and that the experiments conformed to the principles set out in the WMA Declaration of Helsinki and the Department of Health and Human Services Belmont Report.	Not Applicable	
Studies involving **human participants:** For publication of **patient photos**, include a statement confirming that consent to publish was obtained.	Not Applicable	
Studies involving experimental **animals**: State details of **authority granting ethics approval** (IRB or equivalent committee(s), provide reference number for approval. Include a statement of compliance with ethical regulations.	Not Applicable	
Studies involving **specimen and field samples:** State if relevant **permits** obtained, provide details of authority approving study; if none were required, explain why.	Not Applicable	

Dual Use Research of Concern (DURC)	Information included in the manuscript?	In which section is the information available? (Reagents and Tools Table, Materials and Methods, Figures, Data Availability Section)
Could your study fall under dual use research restrictions? Please check biosecurity documents and list of **select agents and toxins** (CDC): https://www.selectagents.gov/sat/list.htm	Not Applicable	
If you used a select agent, is the security level of the lab appropriate and reported in the manuscript?	Not Applicable	
If a study is subject to dual use research of concern regulations, is the name of the **authority granting approval and reference number** for the regulatory approval provided in the manuscript?	Not Applicable	

Reporting

The MDAR framework recommends adoption of discipline-specific guidelines, established and endorsed through community initiatives. Journals have their own policy about requiring specific guidelines and recommendations to complement MDAR.

Adherence to community standards	Information included in the manuscript?	In which section is the information available? (Reagents and Tools Table, Materials and Methods, Figures, Data Availability Section)
State if relevant guidelines or checklists (e.g., **ICMJE, MIBBI, ARRIVE, PRISMA**) have been followed or provided.	Not Applicable	
For **tumor marker prognostic studies,** we recommend that you follow the **REMARK** reporting guidelines (see link list at top right). See author guidelines, under 'Reporting Guidelines'. Please confirm you have followed these guidelines.	Not Applicable	
For **phase II and III randomized controlled trials**, please refer to the **CONSORT** flow diagram (see link list at top right) and submit the CONSORT checklist (see link list at top right) with your submission. See author guidelines, under 'Reporting Guidelines'. Please confirm you have submitted this list.	Not Applicable	

Data Availability

Data availability	Information included in the manuscript?	In which section is the information available? (Reagents and Tools Table, Materials and Methods, Figures, Data Availability Section)
Have **primary datasets** been deposited according to the journal's guidelines (see 'Data Deposition' section) and the respective accession numbers provided in the Data Availability Section?	Yes	Data Availability
Were **human clinical and genomic datasets** deposited in a public access-controlled repository in accordance to ethical obligations to the patients and to the applicable consent agreement?	Not Applicable	
Are **computational models** that are central and integral to a study available without restrictions in a machine-readable form? Were the relevant accession numbers or links provided?	Yes	Data Availability
If publicly available data were reused, provide the respective **data citations in the reference list**.	Yes	Materials and Methods
